# Biochemical characterisation of four rhamnosidases from thermophilic bacteria of the genera *Thermotoga*, *Caldicellulosiruptor* and *Thermoclostridium*

**DOI:** 10.1038/s41598-019-52251-0

**Published:** 2019-11-04

**Authors:** Melanie Baudrexl, Wolfgang H. Schwarz, Vladimir V. Zverlov, Wolfgang Liebl

**Affiliations:** 1 Technical University of Munich, Department of Microbiology, Emil-Ramann-Str. 4, 85354 Freising, Germany; 2 Aspratis GmbH, Hübnerstr. 11, 80637 München, Germany; 3 Institute of Molecular Genetics, Russian Academy of Science, Kurchatov Sq. 2, 123182 Moscow, Russia

**Keywords:** Enzymes, Proteins

## Abstract

Carbohydrate active enzymes are classified in databases based on sequence and structural similarity. However, their function can vary considerably within a similarity-based enzyme family, which makes biochemical characterisation indispensable to unravel their physiological role and to arrive at a meaningful annotation of the corresponding genes. In this study, we biochemically characterised the four related enzymes Tm_Ram106B, Tn_Ram106B, Cb_Ram106B and Ts_Ram106B from the thermophilic bacteria *Thermotoga maritima* MSB8, *Thermotoga neapolitana* Z2706-MC24, *Caldicellulosiruptor bescii* DSM 6725 and *Thermoclostridium stercorarium* DSM 8532, respectively, as α-l-rhamnosidases. Cobalt, nickel, manganese and magnesium ions stimulated while EDTA and EGTA inhibited all four enzymes. The kinetic parameters such as K_m_, V_max_ and k_cat_ were about average compared to other rhamnosidases. The enzymes were inhibited by rhamnose, with half-maximal inhibitory concentrations (IC_50_) between 5 mM and 8 mM. The α-l-rhamnosidases removed the terminal rhamnose moiety from the rutinoside in naringin, a natural flavonone glycoside. The *Thermotoga* sp. enzymes displayed the highest optimum temperatures and thermostabilities of all rhamnosidases reported to date. The four thermophilic and divalent ion-dependent rhamnosidases are the first biochemically characterised orthologous enzymes recently assigned to glycoside hydrolase family 106.

## Introduction

The classification system of glycoside hydrolases (GH) in the Carbohydrate Active enZymes database CAZy^1^ (www.cazy.org) is based on sequence similarity and secondary structure. Catalytic mechanism and key catalytic residues are greatly conserved in the GH families allowing prediction for newly detected enzymes^2^. However, in GH family 2 (GH2), activities on 23 different substrates have been observed so far, which shows exemplarily that enzymes of the same family, having high sequence and structural identity, can catalyse the hydrolysis of different substrates. This demonstrates the limits of bioinformatics when it comes to predicting the true enzymatic function of a new protein, which actually requires experimental proof. Another reason for the need of biochemical characterisation is obviously the generation of new evidence based database entries to expand the basis for comparison and classification of new data.

α-l-Rhamnosidases (EC 3.2.1.40) are enzymes that catalyse the hydrolysis of α-L-rhamnosyl-linkages in compounds containing terminal α-l-rhamnose. Rhamnosidases are considered to be useful for various applications, of which many involve flavonones with terminal rhamnosyl moieties such as naringin, hesperidin, rutin, toxerutin or diosmin^3,4^. Rhamnosidases are useful for debittering, due to the less bitter taste of the de-rhamnosylated flavanones^5^, for rhamnose production^6^, and for the determination of the anomeric configuration in polysaccharides, glycosides and glycolipids^7^. These enzymes may enhance wine aroma^8^ and flavonoid bioavailability^9^, or assist in the synthesis of pharmaceuticals^10^. Biochemical analysis of their activity pattern e.g. towards flavonone glycosides can reveal whether new rhamnosidases are of industrial interest.

Most of the hitherto characterised rhamnosidases are members of family GH78. They are able to degrade *para*-nitrophenyl-α-l-rhamnopyranoside (*p*NPR), used as a model substrate for rhamnohydrolase activity, or to cleave the terminal α-l-rhamnose from natural products^11^. 3D structures of four bacterial enzymes of this family show an (α/α)_6_-barrel as the catalytic module^12–15^. Only one enzyme of GH28 (Rham28 from *Aspergillus niger*) has been shown to have rhamnosidase activity and was able to degrade *p*NPR^16^. However, it did not show activity towards the glycosylated flavonones naringin or hesperidin.

Rha106M from *Sphingomonas paucimobilis* FP2001, was the first enzyme assigned to GH106 displaying activity on *p*NPR. This enzyme’s sequence identity to the previously described rhamnosidases of GH78 was too low and a new GH family was created^17^. The only 3D structure for a GH106 enzyme, α-l-rhamnosidase BT_0986 from *Bacteroides thetaiotaomicron* VPI-5482, revealed an N-terminal (α/β)_8_-barrel architecture as catalytic module^18^. Hence, to date rhamnosidase activity has been demonstrated for bacterial and eukaryotic enzymes of three GH families, namely GH28, GH78 and GH106.

Thermostable enzymes, usually isolated from thermophilic organisms, have an obvious advantage as catalysts in industrial processes, as higher temperatures promote better substrate solubility, increase enzyme turnover numbers and improve the stability against microbial contamination. With regard to naringin, for instance, the bitterness causing substance in grapefruit, only about 7 g/l are soluble at 55 °C, whereas at 75 °C more than 100 g/l can be dissolved in water^19^.

*Thermoclostridium stercorarium* (formerly *Clostridium stercorarium*) was isolated in 1983 from a compost heap and described as an anaerobic, spore forming, cellulolytic thermophile, with an optimal growth temperature of 65 °C^20,21^. *Thermotoga maritima* and *Thermotoga neapolitana* are rod-shaped, strictly anaerobic, monotrichously flagellated hyperthermophilic bacteria, growing up to 90 °C with an optimum around 80 °C^22,23^. *Caldicellulosiruptor bescii* (formerly *Anaerocellum thermophilum*) was isolated from a thermal spring in Kamchatka, Russia^24^, and characterised as an extremely thermophilic, anaerobic, asporogenous, cellulolytic bacterium with optimal growth between 78 °C and 80 °C^25^.

One of our earlier studies on the hemicellulolytic potential of *Thermoclostridium stercorarium* subsp. *stercorarium* revealed an enzyme (AGC67072.1), from here on referred to as Ts_Ram106B, with minor α-l-rhamnosidase activity that was previously listed in the GH non-classified family, due to the lack of characterisation of any homologs^26^. More recently, it has been assigned to the GH family 106, since α-l-rhamnosidase activity has also been shown for two related enzymes of *Niabella aurantiaca* (WP_018627535.1) and *Paenibacillus* sp. JDR-2 (ACT02314.1), respectively, by Helbert *et al*.^27^.

Here we report the characterisation of Ts_Ram106B from *Tc. stercorarium* and the orthologous proteins Tm_Ram106B, Tn_Ram106B and Cb_Ram106B from *T. maritima*, *T. neapolitana* and *C. bescii*, respectively.

## Results

### Selection of candidate enzymes and homology analysis

The first 72 hits of a BLASTp search with the sequence of Ts_Ram106B as query sequence and all proteins listed in the non-classified CAZy GH family (as of February 2019) with either over 70% query coverage (QC) or sequence identities (ID) above 20% are shown in Table S1 of the supplementary data. Three proteins from thermophilic and hyperthermophilic bacteria were selected for further characterisation.

Table 1 gives an overview of the similarity of these proteins from *Thermoclostridium stercorarium* (Ts_Ram106B), *Thermotoga maritima* (Tm_Ram106B), *Thermotoga neapolitana* (Tn_Ram106B) and *Caldicellulosiruptor bescii* (Cb_Ram106B). Sequence identity values ranged between 28% (Ts_Ram106B *vs* Tm_Ram106B) and 79% (between the two *Thermotoga* enzymes) and sequence coverages of 70 and 99% to each other. Two GH106_dist proteins of GH family 106 from *Niabella aurantiaca* (ACT02314.1) and *Paenibacillus* sp. (WP_018627535.1) exhibited rhamnosidase activity in a recent study^27^. They were the reason for the four rhamnosidases of this study (Ram106Bs) to be assigned to GH106 and were therefore included in the similarity analysis in Table 1. The enzyme of *N. aurantiaca* with 75–98% QC and 27–29% ID to the Ram106Bs would have been within the initially defined limits, which were used to constrain the list of candidate proteins, and is the nearest homologue shown. The next homologous enzymes are the other GH106_dist rhamnosidase from *Paenibacillus* sp. and the rhamnosidases of GH106 (Rha106s), with IDs of at least 19%. Almost no similarity was found to rhamnosidases of family GH78 where the best hits to Tm_Ram106B have 25–28% identity covering only 5–10% of the sequence (QC) (Table 1).

**Table 1:** Homologies of the studied rhamnosidases in comparison to already characterized rhamnosidases of GH106 and GH78.

Protein name (Organism)	Ref.^d^	Accession	Ts_ Ram106B		Tm_ Ram106B		Tn_ Ram106B		Cb_ Ram106B	
			QC^c^	ID^c^	QC	ID	QC	ID	QC	ID
**GH106 from this study**										
Ts_Ram106B (*Thermoclostridium stercorarium*)	This study	AGC67072.1	100	100	95	28	70	36	98	28
Tm_Ram106B (*Thermotoga maritima*)	This study	AGL50002.1	95	28	100	100	99	79	99	60
Tn_Ram106B (*Thermotoga neapolitana* Z2706-MC24)	This study	ACM23671.1^e^	70	36	99	79	100	100	99	61
Cb_Ram106B (*Caldicellulosiruptor bescii)*	This study	ACM61646.1	98	28	99	60	99	61	100	100
**GH106_dist**										
B160DRAFT_04058 (*Niabella aurantiaca* DSM 17617)	Helbert *et al*.^27^	WP_018627535.1	75	29	98	27	98	27	98	27
Pjdr2_3683 (*Paenibacillus* sp. JDR-2)	Helbert *et al*.^27^	ACT02314.1	63	20	59	20	61	19	53	21
**Characterized GH106 rhamnosidases** ^**a,b**^										
BT_0986 (*Bacteroides thetaiotaomicron* VPI-5482)	Luis *et al. et al*.^56^	AAO79250.1	37	19	40	34	41	25	42	34
RhaM (*Sphingomonas paucimobilis* FP2001/JCM 10661)	Miyata *et al*.^17^	BAD12237.1	18	24	53	23	35	21	39	24
RHA-P (*Novosphingobium* sp. PP1Y)	Mensitieri *et al*.^34^	CCA90848.1	30	23	33	27	30	21	35	27
**GH78 (best hits with Tm_Ram106B)** ^**b**^										
BT_1019 (*Bacteroides thetaiotaomicron* VPI-5482)	Ndeh *et al*.^18^	AAO76093.1	4	35	17	25	5	38	3	33
Rha78A (*Streptomyces avermitilis* MA-4680 = NBRC 14893)	Fujimoto *et al*.^14^	BAC68538.1	2	43	18	25	8	28	6	32
Ram2 (*Pediococcus acidilactici* DSM 20284)	Michlmayr *et al*.^57^	ZP_07366943.1	—	—	10	27	10	33	9	23
RamA (*Lactobacillus acidophilus* NCFM)	Beekwilder *et al*.^9^	AAV43293.1	6	36	17	28	1	42	—	—

^a^α-l-rhamnosidase activity [EC. 3.2.1.40], excluding BT_4145 (accession No. AAO79250.1, *Bacteroides thetaiotaomicron* VPI-5482) with rhamnogalacturonan α-l-rhamnohydrolase activitiy [E.C. 3.2.1.175].

^b^Just experimentally evidenced activity on *p*NPR, excludes BN863_22040 and BN863_22090 (Formosa agariphila KMM 3901), which are listed in GH106 and GH78 as characterized.

^c^Query coverage (QC) and identity (ID) in percent (%) determined by NCBI tool BLASTp.

^d^Reference for experimental proof of rhamnosidase activity.

^e^Accession number for the sequence of *Thermotoga neapolitana* DSM 4359.

**Figure 1:**
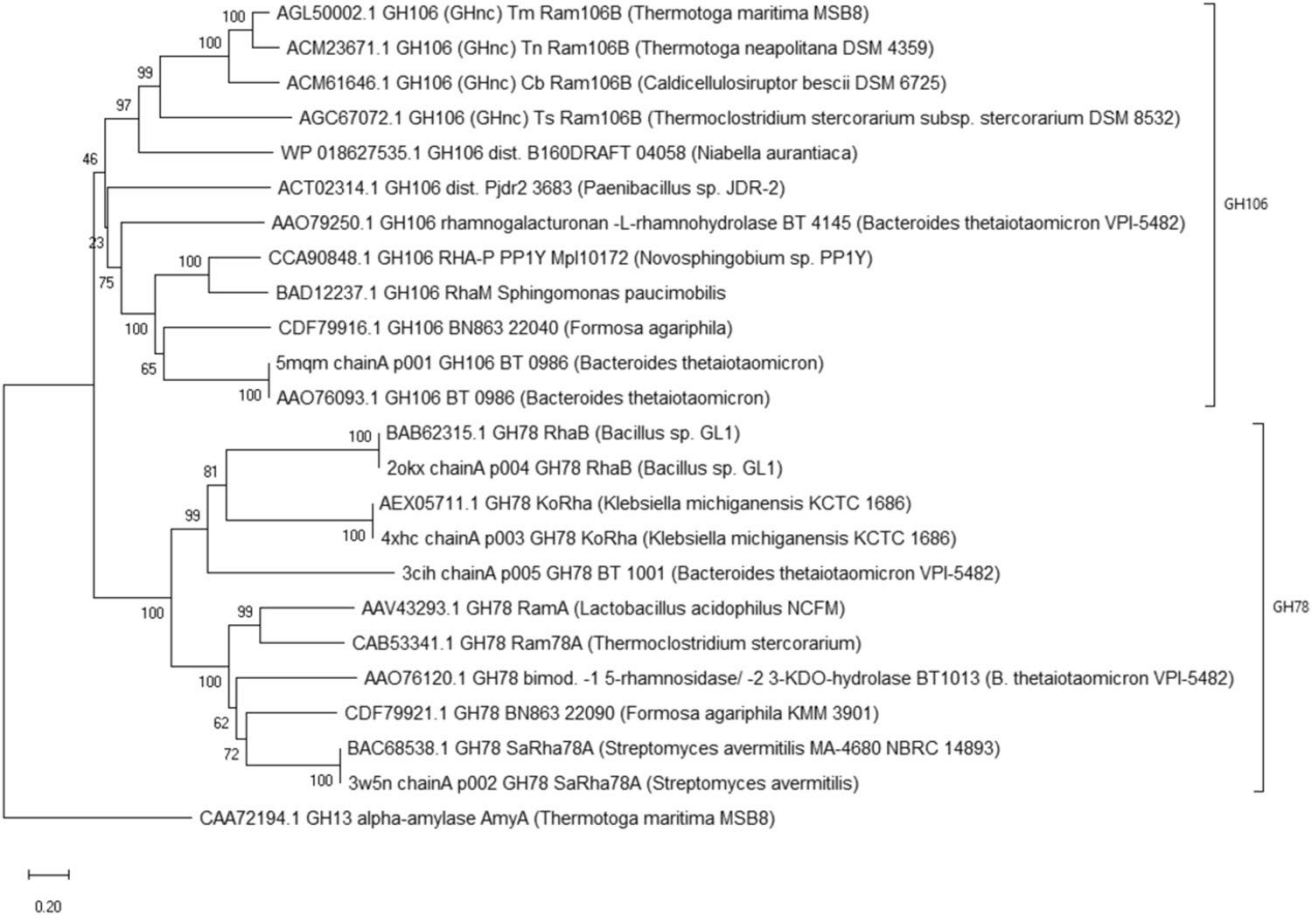
Neighbour-joining tree based on a structural alignment (generated by PromalS3D) with amino acid sequence of rhamnosidases and one GH13 enzyme as an outgroup^53^. Protein accession numbers are followed by CAZy family affiliation, protein name(s) and organism. Sum of branch length = 14.02, percentages of replicate trees in the bootstrap test (1000 replicates) are shown next to the branches^54^, scale is evolutionary distance computed using Poisson correction method^55^ in numbers of amino acid substitutions per site (pairwise deletion of ambiguous position), analysis was conducted in MEGA X^46^.

To visualise differences in sequences and clustering of rhamnosidases of the two main GH families containing EC.3.2.1.40 enzymes, a multiple sequence alignment (Fig. S1) including information about secondary structures derived from crystal structures was generated and used to calculate a neighbor-joining tree (Fig. 1). Information on the proteins used can be found in Table S3. The four Ram106Bs were included in this comparison, as well as the two other GH106_dist proteins, five characterised rhamnosidases of GH106, seven rhamnosidases of GH78 and one GH13 alpha-amylase from *T. maritima* that was chosen as outgroup for the phylogenetic tree analysis (Fig. 1).

All rhamnosidases of GH78 are clearly separated from the other rhamnosidases in the neighbour-joining tree. All enzymes now belonging to GH106 form a common clade, although in some cases they have only very low sequence identities (e.g. 19% between Ts_Ram106B and BT_0986), as shown in Table 1. This branch can additionally be subdivided into the already established characterised GH106 rhamnosidases (Rha106s) and the Ram106Bs investigated in this study. While the branch with the Rha106s also contains the GH106_dist enzyme of *Paenibacillus* sp., the other GH106_dist rhamnosidase of *Niabella aurantica* clusters together with the Rha106Bs.

An additional sequence alignment (Fig. S2) was made to compare the GH106 enzymes separately and to verify the presence of catalytic amino acids known from the crystal structure of BT_0986 (PDB: 5MQM, Fig. S2). The sequence alignment highlights the possible general base and the possible general acid which contribute to hydrolysis of α-l-rhamnosidic bonds as inferred from published crystal structure, as well as residues proposed to interact with the calcium ion in the active center^18^. The general base of BT_0986 (E593) aligned well with each of the investigated sequences. The corresponding amino acid positions of the four Ram106Bs are E335 for Tm_Ram106B (AGL50002.1) and Tn_Ram106B (ACM23671.1), E336 of Cb_Ram106B (ACM61646.1), and E386 of Ts_Ram106B (AGC67072.1). Ram106B sequences share just one out of four metal ion binding residues with BT_0986 (D458 of BT_0986), whereas for RHA-P (CCA90848.1), RhaM (BAD12237.1) and a rhamnosidase from *Formosa agariphila* (WP_084817526.1) corresponding amino acids to the catalytic acid (E461) and two additional calcium binding residues (S459, E538) to BT_0986 could also be found.

It is also noteworthy that the *Niabella* enzyme matches the amino acid residues of Ram106Bs in almost all positions where the Ram106Bs amino acids do not match those of BT_0986. For example, aspartic acid (D458) followed by serine in BT_0986 becomes aspartate and glutamate in Ram106Bs and the enzyme of *Niabella*, and E538 and E561 become histidines and aspartic acids respectively. These differences, indicating changes in the architecture of the catalytic center, may warrant the definition of a new subfamily within GH106.

### Cloning, expression and purification

The newly constructed plasmids pET24c-Tma_*ramB*, pET24c-Tne_*ramB* and pET24c-Cbe_*ramB*, containing the full length sequences were used for expression in addition to pET24c-Tst_*ramB*. No signal peptide was predicted by SignalP 4.1. The C-terminally His_6_-tagged proteins Tm_Ram106B, Tn_Ram106B, Cb_Ram106B and Ts_Ram106B were purified by IMAC and heat treatment at 55 °C. Protein bands corresponding to the respective theoretical molecular masses of 116.6 kDa, 115.8 kDa, 115.9 kDa and 121.3 kDa were detected on SDS-PAGE. Additional bands in the higher molecular weight range, above the 180 kDa band of the protein marker, could be detected for all preparations but Ts_Ram106B (Fig. S3). Increasing the SDS concentration or the denaturation time of the samples prior to SDS-PAGE as well as addition of 5 or 10 mM EDTA, did not resolve the additional bands (Fig. S4). It is presumed that the bands represent incompletely denatured proteins, since conspicuously these bands more frequently occur for more thermostable orthologs.

The oligomerisation state was studied by gel filtration, using cell extract of an *E. coli* culture producing Tm_Ram106B, heat-treated at 75 °C and concentrated threefold, as sample. In two elution fractions *p*NPR-rhamnosidase activity could be detected. The retention times of these active fractions pointed to the presence of Tm_Ram106B-monomers (116.6 kDa) and -dimers (233.2 kDa).

### Activity on *p*NP-α-l-rhamnoside

Of all *para*-nitrophenyl-glycosides tested (see Table 4), exclusively *p*NP-α-l-rhamnoside (*p*NPR) was cleaved by the purified enzymes. It was therefore used as substrate for characterisation. A summary of the enzymatic properties, determined in this study, can be found in Table 2.

**Table 2:** Properties of the rhamnosidases analyzed in this study.

Protein	strain	MW (kDa)	theor. PI	fold change in activity with CoCl_2_^a^	specific acitivty^a,b^ (U mg^−1^)	Temp.Opt. (°C)	pH 60% activity range	K_m_ or K_prime_, h^b,c^ (mM)	k_cat_^b^ (s^−1^)	K_i_^b^, α^b^ (mM rhamnose)	IC_50_ (at K_m_ or K_prime_)
Tm_Ram106B	*Thermotoga maritima* MSB8	116.6	5.95	25.1	40.5 ± 0.33	86.5	5.5–7	1.94 ± 0.20	217.8 ± 10.1	2.37 ± 0.13	4.9
Tn_Ram106B	*Thermotoga neapolitana* Z2706-MC24	115.8	5.89	60.8	32.23 ± 0.19	84	5–6.6	1.47 ± 0.14	146.8 ± 6.1	5.50 ± 1.01, 1.40 ± 0.44	6.5
Cb_Ram106B	*Caldicellulosiruptor bescii* DSM 6725	115.9	6.31	52.9	7.38 ± 0.21	70	5–6.6	1.29 ± 0.23, 1.64 ± 0.20	51.9 ± 6.0	—	5.5
Ts_Ram106B	*Thermoclostridium stercorarium* subsp. stercorarium DSM 8532	121.3	5.63	6.9	2.13 ± 0.01	50	5–5.6	1.90 ± 0.18	14.3 ± 0.5	4.94 ± 0.75, 2.56 ± 0.63	8.3

^a^On 1 mM *p*NPR with 1 mM CoCl_2_.

^b^±standard deviation.

^c^K_prime_/hill-coefficient h for Cb_Ram106B.

### Stimulation of activity by divalent metal ions

**Figure 2:**
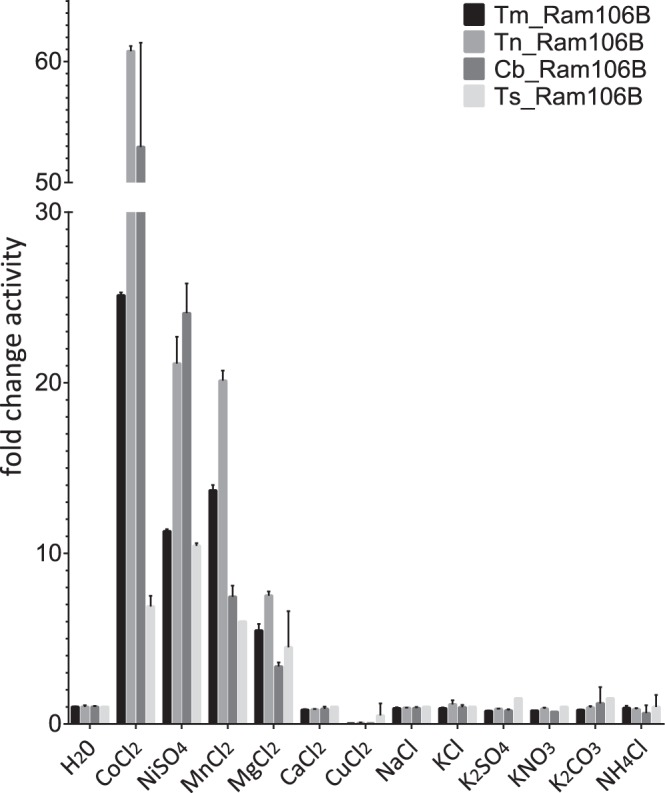
Influence of metal ion addition on pNP-rhamnosidase activity. Standard reactions (1 mM pNPR, 100 mM MOPS pH 7, 10 min) were performed as described in materials and methods, with 50 nM of Tm_Ram106B (80 °C), Tn_Ram106B (80 °C), Cb_Ram106B (70 °C) and Ts_Ram106B (55 °C) with 1 mM of different salt solutions. Activities are expressed relative to reactions without additional ions (H_2_O). Error bars show standard deviation of triplicates.

Some divalent metal ions had a significant stimulating effect on the activity of all studied enzymes. The highest activity increase for Tm_Ram106B, Tn_Ram106B and Cb_Ram106B was observed with cobalt, followed by manganese, nickel and magnesium (Fig. 2). For the enzyme of *Tc. stercorarium* nickel ions had the greatest activating effect, followed by cobalt, manganese and magnesium. None of the other ions tested increased or reduced the activity for any of the enzymes in comparison to reactions without additional ions (Fig. 2), apart from CuCl_2_, which entirely inhibited all of the Ram106Bs. Furthermore, the four rhamnosidases could be almost completely inhibited by the addition of 5 mM EDTA (residual activities between 0.2 and 0.5%) or 5 mM EGTA (residual activities between 0.4 and 1.2%). Based on this observation, 1 mM CoCl_2_ was added to all reactions with the four enzymes.

### Temperature and pH optima

Using a 10 min assay, the apparent temperature optima at optimum pH for Tm_Ram106B, Tn_Ram106B, Cb_Ram106B and Ts_Ram106B were 86.5 °C, 84 °C, 70 °C and 50 °C, and more than 80% activity were observed in the ranges of 76.5–93 °C, 74–91 °C, 62–76 °C and 36–63 °C, respectively (Fig. S5). The highest temperature optimum for previously published bacterial rhamnosidases was 70 °C for the enzyme from *Thermomicrobium* sp.^28^. This is surpassed by Tm_Ram106B and Tn_Ram106B by at least 14 °C. These two hyperthermophilic enzymes exhibited considerable resistance against thermoinactivation at 80 °C (Table 3). Nearly no loss in activity was observed at this temperature, with 73% (Tm_Ram106B) and 68.5% (Tn_Ram106B) residual activity after 12 h of incubation. The half-life of more than 12 h at this temperature is comparable to other heterologously produced enzymes of this thermophilic genus^29^. At 90 °C heat inactivation was more severe, in particular for Tn_Ram106B which was almost completely inactivated after 12 h. During the first hour of incubation, at either 80 °C or 90 °C, an increase in relative activities of Tm_Ram106B was observed, before the enzyme began to inactivate (Fig. S6).

**Table 3:** Thermostability of Tm_Ram106B and Tn_Ram106B after 6 and 12 h at 80 and 90 °C.

	Tm_Ram106B		Tn_Ram106B	
Time (h)	80 °C	90 °C	80 °C	90 °C
6	98.1%	47.1%	91.0%	3.0%
12	73.0%	14.3%	68.5%	0.1%

Enzymes were incubated for 6 and 12 h at either 80 °C or 90 °C. Residual activities in percent related to the activity at 0 h were calculated from *p*NP standard reactions (1 mM *p*NPR, 1 mM CoCl_2_, 100 mM MOPS pH 7, 10 min, 80 °C) with enzyme endconcentrations of 50 nM.

The optimal pH-ranges of the four enzymes tested were similar. Relative activities of over 90% were determined between pH 5.5 and 6.6 in the tested buffers for the *T. maritima* enzyme, while the pH range for highest activity was slightly narrower for Tn_Ram106B (pH 5.6–6.3) and Cb_Ram106B (pH 5.0–6.3). For Ts_Ram106B the highest activity was measured in malate buffer at pH 5 (Fig. S7).

### Kinetic analysis

Enzyme concentrations between 2.5 nM for Tm_Ram106B and 50 nM for Ts_Ram106B were suitable to reach approximate substrate saturation at 4 mM *p*NPR (16 mM for Ts_Ram106B), the substrate used for determination of the kinetic parameters. For all but Cb_Ram106B, for which a sigmoidal substrate concentration-velocity curve fitted the best, Michaelis Menten equation was fitted to the data, which are visualized in Fig. S8. Details on model type, calculated parameters, fitting quality and standard errors can be found in Table S4. The apparent dissociation constants K_m_, alternatively K_prime_, range from 1.3 mM (Cb_Ram106B) to 1.9 mM (Tm_Ram106B and Ts_Ram106B). These are approximately within the same range of K_m_ values reported for other bacterial rhamnosidases on *p*NPR, which are between 0.05 and 1.8 mM^30^. Specific activities on 1 mM *p*NPR (standard reaction) were 40.5 U/mg, 32.2 U/mg, 7.4 U/mg and 2.1 U/mg for Tm_Ram106B, Tn_Ram106B, Cb_Ram106B and Ts_Ram106B, respectively (Fig. S8). This is about average when compared to the specific activities of, for example, rhamnosidase from *Fusobacterium* K-6 (2.8 U/mg), Ram78A of *Tc. stercorarium* (82 U/mg), or Rha78A (54 U/mg) and Rha78B (54,4 U/mg) of *Bacillus* sp. GL1^11,31,32^. Catalytic efficiencies ranged from about 14 s^−1^ for Ts_Ram106B to 218 s^−1^ for Tm_Ram106B (Table 2). All enzymes were inhibited by the reaction product rhamnose at relatively low rhamnose concentrations. The inhibitor dissociation constant (k_i_) of Tm_Ram106B with 2.4 mM was the only constant to be calculated from a classical product inhibition model, which should be competitive inhibition, because the product binds to the active center. For Tn_Ram106B and Ts_Ram106B, with k_i_ values of 5.5 mM and 4.9 mM respectively, additional, not precisely identifiable factors seem to play a role in inhibitor binding and dissociation, which is also illustrated mathematically by the factor α (summarized in Table 2; details in Table S4). Half-maximal inhibitory concentrations (IC_50_) at the determined K_m_ of each of the enzymes were between 4.9 mM (Tm_Ram106B) and 8.3 mM (Ts_Ram106B). The fact that, for the most commonly known inhibition types, the k_i_ values can vary between IC_50_/2 and IC_50_ (with [S] = K_m_)^33^ implies that for Cb_Ram106B with an IC_50_ value of 5.5, the k_i_ values can only be in the range of 2.25 and 5.5 mM. Kinetic and inhibition parameters together with pH- and temperature optima and some other enzymatic properties are summarized in Table 2.

### Naringin degradation

**Figure 3:**
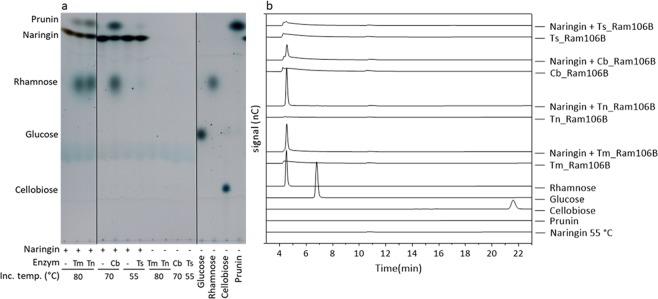
Analysis of naringin hydrolysates produced by rhamnosidases from *T*. *maritima*, *T*. *neapolitana*, *C*. *bescii* and *Tc*. *stercorarium*. Reactions contained 6 mM naringin, 1 mM CoCl_2,_ 100 mM MOPS pH 7 (RT) and 50 nM of Tm_Ram106B or Tn_Ram106B, or 100 nM Cb_Ram106B or Ts_Ram106B. Reactions were analysed after 4 h of incubation at 80 °C for the two first mentioned enzymes, 70 °C for Cb-Ram106B and 55 °C for Ts_Ram106B. (**a**) TLC was performed as described in materials and methods with 6 µl of each reaction and a total amount of 2 µg of the reference-sugars, and 4 µg of prunin. Presence of naringin and enzymes is indicated by + or the first two letters of the enzyme names. Not relevant lanes were cropped from the image (black lines). (**b**) HPAEC-PAD chromatograms of naringin hydrolysates and several standards. Analysis by HPAEC-PAD was performed as described in material and methods. 50 mg/l of rhamnose, glucose, cellobiose and prunin were used as external standards. Rhamnose was detected in all reactions containing naringin and a rhamnosidase.

Naringin, a flavonone glycoside found in citrus peels, was chosen to test the ability of the rhamnosidases to degrade a natural substrate with a terminal rhamnosidic moiety that is available in large amounts. Both TLC and HPAEC-PAD analysis demonstrated the liberation of rhamnose from naringin by all tested rhamnosidases (Fig. 3). For Tm_Ram106B, Tn_Ram106B and Cb_Ram106B the cleavage products of naringin, i.e. prunin and rhamnose, were easily detected with both analytical techniques, whereas for Ts_Ram106B the cleavage product spots detected by TLC were faint and barely detectable with HPAEC-PAD, indicating low activity.

The specific activity of Tm_Ram106B on naringin was examined exemplarily, as it is the most active and thermostable rhamnosidase of this study. Using a saturated naringin suspension (10% w/v) with 5 nM Tm_Ram106B at 80 °C and a 40 min assay period, the specific rhamnose liberation activity was found to be 2.7 U/mg (Table S2).

## Discussion

In a previous study on the hemicellulolytic potential of *Thermoclostridium stercorarium*, an enzyme, now named Ts_Ram106B, exhibited rhamnosidase activity^26^. The aim of this study was to characterise a number of putative glycoside hydrolases related to Ts_Ram106B that were identified in the GHnc (Glycoside Hydrolase Family “Non Classified”) section of the CAZy database (as of February 2019) by BLASTp analysis in order to contribute to their classification and to compare their enzymatic properties. The BLASTp results were constrained to hits with more than 70% query coverage (QC) and more than 20% sequence identity (ID) (Table S1). From this list, three enzymes were characterised, namely Tm_Ram106B, Tn_Ram106B and Cb_Ram106B, from the hyperthermophilic bacteria *Thermotoga maritima* and *Thermotoga neapolitana*, and the thermophilic species *Caldicellulosiruptor bescii*, respectively. The selection turned out to be efficient in terms of their shared rhamnosidase activity and the clustering in the same branch of a neighbour-joining tree (Fig. 1).

Amino acid sequence comparison showed only little similarity of the investigated enzymes to already characterised rhamnosidases of GH106 and almost no similarity to enzymes from the family GH78 which harbors most of the hitherto characterised rhamnosidases (Table 1). Two enzymes, distantly related to GH106 (therefore referred to as GH106_dist), were included in the amino acid sequence and similarity analysis due to their rhamnosidase activity in a recently published study^27^. The analysis showed that the rhamnosidase from *Niabella aurantiaca* (ACT02314.1) is nearly as homologous to the Ram106Bs as the four Ram106Bs amongst each other. A multiple structural sequence alignment and the inferred neighbour-joining tree with sequences of Ram106Bs of this study, enzymes of GH106_dist^27^, and some characterized members of GH106 and GH78 supports the grouping of the enzymes of this study into GH family 106. Mensitieri *et al*.^34^ discussed the family affiliation and possible catalytic mechanism of RHA-P from *Novosphingobium* sp. PP1Y by matching amino acids of RHA-P to the residues of the catalytic center of BT_0986 from *Bacteroides thetaiotaomicron* (as derived from PDB crystal structure 5MWK) in a pairwise alignment. The catalytic base (E593 in BT_0986) was conserved in RHA-P and just one (E561 in BT_0986) out of five calcium binding residues (E593, E538, D458, S459, E561) was missing in the pairwise alignment. As E561 was not binding the calcium ion in all crystallised versions of BT_0986 and is thought to assist in binding of an arabinose residue of the specific substrate used by BT_0986, the authors suggested a similar catalytic mechanism for RHA-P, but activity on a different substrate.

The structural alignment in this study, using PromalS3D as the alignment tool and the PDB crystal structure 5MQM as reference, shows all of the matchings mentioned already by Mensitieri *et al*.^34^. In addition, the catalytic acid E461 of BT_0986 suggested by Ndeh *et al*.^18^ was also found in RHA-P.

For the four rhamnosidases studied here, in contrast to RHA-P, only the catalytic base and two of the six amino acids contributing to calcium binding in the PDB structure 5MQM of BT_0986, align well in Fig. S2. Both, the amino acid serving as general acid in catalysis and the majority of metal binding residues of the rhamnosidases of this study do not match the BT_0986 residues, but appear to be conserved among each other. This is also reflected in the neighbour-joining tree and indicates that the investigated rhamnosidases Tm_Ram106B, Tn_Ram106B, Cb_Ram106B and Ts_Ram106B have a different architecture of their active center and could possibly define a new subfamily of GH106. For further support of this hypothesis, experimental data on the protein structures would be of value. Its collective grouping within the neighbor joining tree (Fig. 1), the results from BLASTp analysis mentioned before, and the alignment of GH106 enzymes indicate that the possible new subfamily of GH family 106 with the four rhamnosidases of this study should also include the enzyme of *N. aurantiaca*. The *Paenibacillus* GH106_dist protein on the other hand at present is not considered to be a member of this new GH106 subfamily. The bootstrap value of less than fifty percent at the corresponding branch, that divides the *N. aurantiaca* from the *Paenibacillus* protein, however, indicates that the demarcation is not clear at this point. It should also be noted that we have only included already characterized rhamnosidases in the analysis to ensure that the enzymes have the same function, and this might not reflect the overall sequence variability in the classical GH106 family.

Among all *para*-nitrophenyl-substrates tested, the recombinantly produced enzymes showed activity solely on the α-l-rhamnoside (*p*NPR). Metal ion dependency was observed for the studied enzymes, as has also been shown for BT_0986 and RHA-P. However, the highest activity was observed with Co^2+^ or Ni^2+^ ions, and addition of Mn^2+^ or Mg^2+^ also resulted in higher specific activities, but additional Ca^2+^ ions did not positively effect rhamnosidase activity. The importance of divalent ions was confirmed by the complete loss of activity by addition of EDTA. Dependence on metal ions in general could be due to stabilising effects, which have often been found in thermostable enzymes^35,36^, or their direct involvement in catalysis. Another purpose that the ions might serve could be, for example, to interact with the substrate to facilitate transition state, and/or positioning of a water molecule for the nucleophilic attack on the C1 of the substrate^18,37,38^.

The additional bands on the SDS-PAGE (Figs S3, S4) for the three most thermophilic Ram106Bs of this study were originally assumed to represent dimer bands, because for Tm_Ram106B active dimers were indicated by gelfiltration under native conditions. However, non-covalently bound dimers would have separated under denaturing SDS conditions, which is why the aforementioned bands are more likely not fully denatured proteins, as it is conspicuous that these bands more frequently occur for the more thermostable orthologs.

The four rhamnosidases studied here were most active at neutral to slightly acidic pH, which is a common feature for bacterial rhamnosidases^39^. This corresponds to the lifestyles of their respective hosts. As expected, the measured temperature optima differed between the enzymes, but were in a good agreement with the growth optima of their original hosts, whereby the two *Thermotoga* enzymes were found to be the most thermophilic bacterial rhamnosidases described to date, with extraordinarily high stability against thermoinactivation. The dimerization, which was shown for Tm_Ram106B, could be considered to also contribute to its thermostability. The kinetic parameters of the Ram106Bs with *p*NPR as a substrate are similar to those of the rhamnosidases described in the literature.

The processing of natural flavonone glycosides, such as naringin, is a potential application for rhamnosidases^40^. All of the four studied rhamnosidases were able to cleave the 1,2-linked terminal rhamnose from naringin and released prunin. A total conversion of 6 mM naringin after just one hour, as described by De Lise *et al*., could not be observed for any of the four studied rhamnosidases^41^. This could be explained by the marked product inhibition, with single-digit mM-range k_i_ and IC_50_ values.

Tm_Ram106B from *T. maritima* was chosen to further investigate activity against naringin, because it had the highest specific activity towards *p*NPR and the highest resistance against thermoinactivation. The enzyme had relatively low specific activity of about 2.7 U/mg on a saturated naringin suspension, measured after 40 min at 80 °C, indicating that the natural substrate for the rhamnosidase is still to be found. The most recent example how complex the natural substrate for rhamnosidases may be, is an already characterised GH106 enzyme (BT_0986). The suggested substrate for BT_0986 is a defined heptasaccharide derived from chain B of rhamnogalacturonan II in pectin^18^ For *T. maritima* MSB8 many carbohydrate utilization loci have been investigated which are composed of different sugar catabolic enzymes, transcription factors and ABC-family transporters. Some of the latter are suggested to have an oligosaccharide import function in glucose, arabinose and xylose containing oligosaccharides^42^. The fact that Tm_Ram106B (gene locus: Tm1074) and various ABC transporters are encoded together in a co-regulated gene cluster in the *T. maritima* MSB8 genome (GenBank: CP004077.1), may indicate the utilization of rhamnose-containing oligosaccharides. In addition, due to the lack of signal peptides an intracellular localization is proposed for all of the Ram106Bs, which reinforces the assumption of an oligosaccharide or glycoconjugate, rather than a polysaccharide, as the most likely substrate.

In conclusion, this study provides the characterisation of four enzymes as metal ion dependent, thermophilic rhamnosidases (Table 2), including the most thermoactive and thermostable representatives of this enzyme type described to date, and supports the recent classification of Ts_Ram106B and its homologs listed in Table S1 into GH family 106.

## Material and Methods

### Plasmids, strains and chemicals

Strains, DNA and substrates used in this study are listed in Table 4. Recombinant *Escherichia coli* strains were grown in LB-Lennox medium with 50 µg/ml kanamycin at 37 °C. The source of chemicals is listed; other chemicals were obtained from Merck (Darmstadt, Germany).

**Table 4:** Strains, DNA and substrates used in this study.

Strain/DNA/substrates	Description or sequenence (5′-3′)	Source
**Strains**		
*Escherichia coli* TOP10	F- mcrA Δ(mrr-hsdRMS-mcrBC) φ80lacZΔM15 ΔlacX74 nupG recA1 araD139 Δ(ara-leu)7697 galE15 galK16 rpsL(Str^R^) endA1 λ^−^	Invitrogen, Carlsbad, USA
*Escherichia coli* BL21 Star^TM^	*E. coli* str. B F^−^ *ompT gal dcm lon hsdS*_*B*_(*r*_*B*_^−^*m*_*B*_^−^) *rne131* λ(DE3 [*lacI lacUV5*-*T7p07 ind1 sam7 nin5*]) [*malB*^+^]_K-12_(λ^S^)	Invitrogen, Carlsbad, USA
**Genomic DNA**		
*Thermotoga maritima* MSB8		Liebl *et al*. 1997
*Thermotoga neapolitana* Z2706-MC24		Dakhova *et al*. 1993
*Caldicellulosiruptor bescii* DSM 6725	former *Anaerocellum thermophilum*	Zverlov *et al*. 1998
**Primer**		
Tma-*ramB-*fw	CTTTAAGAAGGAGATATACAGTGAACCTGAAGGATCTGGAAAA	This study
Tma-*ramB-*rev	CTCAGTGGTGGTGGTGGTGGTGCCGTGAAGAAGAGCTGAGAAC	This study
Tne-*ramB-*fw	CTTTAAGAAGGAGATATACAGTGAACCTGAAGGATCTGGAAAA	This study
Tne-*ramB-*rev	CTCAGTGGTGGTGGTGGTGGTGCGGTGAAAAAGAACTGAGCACAG	This study
Cbe-*ramB-*fw	CTTTAAGAAGGAGATATACAATGCTTGATTTAGAAAAACT	This study
Cbe-*ramB-*rev	CTCAGTGGTGGTGGTGGTGGTGCACATTGGATGTTAATATAC	This study
**Plasmids**		
pET24c (+)	Kan^R^, C-terminal His•Tag®	Merck, Darmstadt, Germany
pET24c-Tst_*ramB*	coding for Ts_Ram106B (protein ID, accession number: AGC67072.1, WP_015357769.1) from *Thermoclostridium stercorarium* subsp*. stercorarium* DSM 8532	Bröker *et al*. 2018
pET24c-Tma_*ramB*	coding for Tm_Ram106B (protein ID, accession number: AGL50002.1, WP_004080411.1) from *Thermotoga maritima* MSB8	This study
pET24c-Tne_*ramB*	coding for Tn_Ram106B (protein ID, accession number for homolog of *T. neapolitana* DSM 4359: ACM23671.1, WP_015919960.1) from *T. neapolitana Z2706-MC24*	This study
pET24c-Cbe_*ramB*	coding for Cb_Ram106B (protein ID, accession number: ACM61646.1, WP_015908894.1) from *Caldicellulosiruptor bescii DSM 6725*	This study
**Substrates**		
*para*-Nitrophenyl-glycosides	α-l-rhamnopyranoside, α-l-arabinofuranoside, α-l-fucopyranoside, β-l-fucopyranoside, β-d-glucopyranoside, β-d-galactopyranoside, α-d-mannopyranoside, α-d-xylopyranoside	Megazyme (Wicklow, Ireland)
Naringin	no.: XN167836	Carbosynth, Berkshire, UK
Prunin	no.: FN65941	Carbosynth, Berkshire, UK

Fw, forward primer; rev, revers primer; start-codons are underlined.

### Selection of enzymes and homology analysis

The Carbohydrate Active Enzymes database^1^ (CAZy; http://www.cazy.org/) was scoured for homologous proteins using BLASTp (NCBI tool: https://blast.ncbi.nlm.nih.gov/Blast.cgi). Signal peptides were predicted with the SignalP 4.1 server^43^ (http://www.cbs.dtu.dk/services/SignalP-4.1/). Multiple sequences were aligned with PROfile Multiple Alignment with predicted Local Structures and 3D constraints^44^ (http://prodata.swmed.edu/promals3d/promals3d.php) using structural information from the RCSB PDB Protein Data Bank^45^ (http://www.rcsb.org/) and default settings. Neighbor-joining trees from aligned sequences were generated using *MEGA X*: Molecular Evolutionary Genetics Analysis across computing platforms^46^.

### Cloning, expression, and purification of recombinant enzymes

Vector pET24c(+) was linearised with NdeI and XhoI in accordance with supplier’s protocol. The genes coding for Ram106B from *T. maritima* MSB8 (Tm_Ram106B), *T. neapolitana* Z2706-MC24 (Tn_Ram106B) and *C. bescii* DSM 6725 (Cb_Ram106B) have been amplified by PCR using Phusion High-Fidelity DNA Polymerase (Thermo Fisher Scientific, MA, USA), genomic DNA of the respective strains and primer pairs as listed in Table 4. Cloning of pET24c(+) constructs was accomplished by Gibson Assembly^47^ and subsequent transformation of strain *E. coli* DH10b.

Enzyme production in *Escherichia coli* BL21 Star™ was achieved by autoinduction with ZYP-5052 medium^48^. Sonication and subsequent centrifugation (20 min, 38 000 g, 4 °C) were performed to obtain cell extracts. C-terminally His_6_-tagged proteins were purified by immobilised metal ion affinity chromatography (IMAC)^49^ using His-Trap FF columns (GE, Healthcare, Little Chalfont, GB). Remaining *E. coli* proteins were removed by heat treatment of the eluates (15 min at 55 °C; 75 °C for *Thermotoga* enzymes) and centrifugation (15 min, 20 000 g, 4 °C). Vivaspin 500 columns (30 kDa cutoff) were used to concentrate proteins if necessary.

Size and purity of the proteins were confirmed by SDS-PAGE^50^. Protein concentrations were determined spectrophotometrically at 280 nm under denaturing conditions (5 M urea) and using the calculated molar extinction coefficients from the ExPaSy ProtParam tool from the Swiss Institute of Bioinformatics^51^ (https://web.expasy.org/protparam/).

Gel filtration chromatography was conducted using an ÄKTApure 25L1 FPLC system, equipped with a Superdex 200 Increase 10/300 GL column (GE Healthcare Bio-Sciences AB, Uppsala, Sweden). The column was calibrated in accordance with the recommendations of the manufacturer and using the following marker proteins: ferritin (450 kDa), aldolase (158 kDa), albumin (68 kDa), chymotrypsinogen (25 kDa), catalase (24 kDa) and cytochrome C (12,5 kDa). After equilibration with filtered and degassed buffer (0.1 M MOPS pH 6.5, 50 mM NaCl, 10 mM CaCl_2_), a 100 μl sample was loaded onto the column. The recorded retention times were calculated back to elution volumes, which are proportional to the decadic logarithm of the molecular weight.

### Activity assays on *para*-nitrophenyl glycosides

Standard reactions made in triplicates were carried out in a total volume of 50 µl with 1 mM *p*NPR (0.1 M stock solution in dimethyl formamide), 100 mM standard buffer (MOPS; pH 7; RT), 1 mM CoCl_2_, enzyme concentrations ranging from 2.5–100 nM, and for 10 min in a thermocycler. Unless stated otherwise, the assay temperatures, derived from the optimal growth temperature of the natural hosts, were 55 °C for Ts_Ram106B, 80 °C for Tm_Ram106B and Tn_Ram106B, and 70 °C for Cb_Ram106B. After adding two volumes of 1 M Na_2_CO_3_ to stop the reaction, released *p*NP was quantified by measuring the increase in absorption at 405 nm. One unit (U) of enzymatic activity was defined as the amount of enzyme releasing 1 µmol of *p*NP equivalent per minute and was calculated using *p*NP as standard. Substrate specificity was tested on additional *p*NP-glycosides (Table 4), to discover potential secondary activities.

For examining the influence of metal Ions, CuCl_2_, NaCl, CaCl_2_, NiSO_4_, KCl, MnCl_2_, MgCl_2_, ZnCl_2_, CoCl_2_, NH_4_Cl, K_2_SO_4_, KNO_3_, K_2_CO_3_ at final concentrations of either 1 or 10 mM, or 5 mM of the complexing agents EDTA or EGTA, where added to the standard buffer. To determine the optimal pH range, hydrolysis reactions were conducted in 25 mM succinate (4.3–6.2), 50 mM malate (5–6.3), 50 mM MES (5.5–6.1), 100 mM MOPS (5.6–6.1), 25 mM PIPES (6.3–6.6) or 100 mM HEPES buffer (5.5–7), with pH values in brackets measured at 80 °C.

The temperature dependence of enzyme activity was determined by measuring 12 different points over a temperature gradient spanning 40 °C. Thermal stability was assayed by preincubating 78.5 nM enzyme in 175 mM MOPS pH 7 and 1,75 mM CoCl_2_ (1.75 fold more concentrated as in the final standard reaction) at 80 and 90 °C. After incubation for various periods of time, residual activity was determined using the standard reaction conditions.

### Kinetic analysis

Kinetic parameters were calculated with Graphpad using non-linear-regression methods after incubating suitable enzyme amounts with substrate concentrations ranging from 0.25–16 mM *p*NPR. Either Michaelis Menten (1) or the Hill equation, a model of allosteric action (2), which is identical to the first mentioned, if the Hill slope equals 1.0, was used to describe the [substrate] vs. velocity curves. Turnover frequencies (k_cat_) have been determined by fitting E_t_ × k_cat_ instead of V_max_ (Eq. (3)). To assess product inhibition and compute k_i_ values either a model of competitive binding (4) or a mixed-inhibition model (5) were fitted to curves that have been obtained as described above using different rhamnose concentrations (0.5–100 mM). IC_50_, which despite its substrate dependence is still often used to estimate the inhibition strength of an inhibitor, was determined, using Eq. (6). Data points were interpolated at substrate concentrations that equal the respective K_m_ using Graphpad and were related to reactions with [I] = 0, to get *y*.

1 $${{\rm{V}}}_{s}=\frac{{V}_{max}\times [{\rm{S}}]}{({K}_{m}+[{\rm{S}}])}$$

2 $${{\rm{V}}}_{s}=\frac{{V}_{max}\times {[{\rm{S}}]}^{h}}{({{K}_{half}}^{h}+{[{\rm{S}}]}^{h})}$$

3 $${\rm{V}}=\frac{{E}_{t}\times {k}_{cat}\times [{\rm{S}}]}{({K}_{m}+[{\rm{S}}])}$$

4 $${{\rm{V}}}_{s}=\frac{{V}_{max}\times [{\rm{S}}]}{({K}_{m}\times \frac{1+[I]}{{K}_{i}}+[{\rm{S}}])}$$

5 $${{\rm{V}}}_{s}=\frac{{V}_{max}/(1+\frac{[I]}{\alpha \times {K}_{i}})\times [{\rm{S}}]}{({K}_{m}\times \frac{1+[I]}{{K}_{i}})/(1+\frac{[I]}{\alpha \times {K}_{i}})+[{\rm{S}}]}$$

6 $$y=\frac{100}{(\frac{1+[I]}{I{C}_{50}})}$$

with V_s_ = specific activity (U/mg), V_max_ = maximal velocity (U/mg), [S] = substrate concentration (mM), K_m_ = Michaelis Menten constant (mM), h = Hill slope, K_prime_ is related to K_m_, is computed as K_half_^h^ and expressed in mM, V = reaction velocity (µmol/sec), k_cat_ = turnover frequency (1/sec), [I] = concentration of inhibitor (mM rhamnose), k_i_ = inhibitory constant (mM rhamnose), y = relative activities (% of activity without additional rhamnose), IC_50_ = half maximal inhibitory concentration (same units as [I]).

### Chromatographic analysis of the naringin hydrolysis products

To test the ability of the recombinantly produced enzymes to release the terminal rhamnose moiety from naringin (4′,5,7-trihydroxyflavanone-7-rhamnoglucoside), hydrolysis products were analysed by TLC and HPAEC-PAD. Reactions containing naringin (5–200 mM, in H_2_O at 80 °C), enzyme (concentrations ranging from 5–200 nM), 100 mM MOPS pH 6.5 and 1 mM CoCl_2_, were incubated between 5 min and 4 h, at suitable temperatures (80 °C for Tm_Ram106B and Tn_Ram106B, 70 °C for Cb_Ram106B, 55 °C for Ts_Ram106B). The reaction products were separated by thin layer chromatography on TLC silica gel 60 plates (Merck, Darmstadt, Germany) with acetonitrile:H_2_O (80:20) as eluent and detection by spraying with 1% anilin (v/v) and 1% diphenylamin (w/v) in acetone mixed with 0.1 volume 85% H_3_PO_4_.

HPAEC-PAD (High Performance Anion Exchange Chromatography with Pulsed Amperometric Detection) was used to analyze the reaction products after naringin degradation using a Dionex ICS 3000 SP system as described by Mechelke *et al*.^52^. To summarise briefly, reaction supernatants and external standards were diluted 1:10 with ddH_2_O in a sample vial to a total volume of 500 µl. Analytes were separated and detected via HPAEC-PAD using an increasing sodium acetate gradient from 7.5 to 100 mM within a period of 67.5 min. Quantification of rhamnose was achieved by calibration with rhamnose (0.5–50 mg/l end concentration after dilution). While analysis was done with Chromeleon 7.2 software, graphical representation and evaluation was performed via GraphPad Prism 7. One unit (U) of enzymatic activity was defined as the amount of enzyme releasing 1 µmol of rhamnose equivalent per minute.

## Supplementary information

Supplementary information

## Data Availability

All data generated during this study are included in this article or its supplementary information files. Raw datasets are available from the corresponding authors on reasonable request.

## Supplementary information

is available for 10.1038/s41598-019-52251-0.

## References

[1] Lombard V, Golaconda Ramulu H, Drula E, Coutinho PM, Henrissat B (2014). The carbohydrate-active enzymes database (CAZy) in 2013. Nucleic acids research.

[2] Henrissat B (1995). Conserved catalytic machinery and the prediction of a common fold for several families of glycosyl hydrolases. Proceedings of the National Academy of Sciences.

[3] Li B, Ji Y, Li Y, Ding G (2018). Characterization of a glycoside hydrolase family 78 α-l-rhamnosidase from *Bacteroides thetaiotaomicron* VPI-5482 and identification of functional residues. 3 Biotech.

[4] Yadav P, CHAUHAN AK, Singh SP (2017). α-L-rhamnosidase. Sources, production, purification and characterization of the debittering enzyme. International Journal of Biotechnology and Research.

[5] Busto MD, Meza V, Ortega N, Perez-Mateos M (2007). Immobilization of naringinase from *Aspergillus niger* CECT 2088 in poly (vinyl alcohol) cryogels for the debittering of juices. Food chemistry.

[6] Puri M, Banerjee UC (2000). Production, purification, and characterization of the debittering enzyme naringinase. Biotechnology Advances.

[7] Kamiya S, Esaki S, Tanaka R (1985). Synthesis of Some Disaccharides Containing an L-rhamnopyranosylor l-Mannopyranosyl Residue, and the Substrate-specificity of α-l-Rhamnosidase from *Aspergillus niger*. Agricultural and biological chemistry.

[8] Gallego MV, Pinaga F, Ramón D, Vallés S (2001). Purification and Characterization of an α-L-rhamnosidase from *Aspergillus terreus* of Interest in Winemaking. Journal of Food Science.

[9] Beekwilder J (2009). Characterization of rhamnosidases from *Lactobacillus plantarum* and Lactobacillus acidophilus. Applied and environmental microbiology.

[10] Bouriche H, Arnhold J (2010). Effect of Cleome arabica leaf extract treated by naringinase on human neutrophil chemotaxis. Natural product communications.

[11] Zverlov VV (2000). The thermostable α‐L‐rhamnosidase RamA of *Clostridium stercorarium*. Biochemical characterization and primary structure of a bacterial α‐L‐rhamnoside hydrolase, a new type of inverting glycoside hydrolase. Molecular microbiology.

[12] Bonanno JB (2005). New York-Structural GenomiX Research Consortium (NYSGXRC). A large scale center for the protein structure initiative. Journal of Structural and Functional Genomics.

[13] Cui Z, Maruyama Y, Mikami B, Hashimoto W, Murata K (2007). Crystal structure of glycoside hydrolase family 78 α-L-rhamnosidase from *Bacillus* sp. GL1. Journal of molecular biology.

[14] Fujimoto Z (2013). The structure of a *Streptomyces avermitilis* α-L-rhamnosidase reveals a novel carbohydrate-binding module CBM67 within the six-domain arrangement. Journal of Biological Chemistry, jbc..

[15] O’Neill EC (2015). Crystal structure of a novel two domain GH 78 family α‐rhamnosidase from *Klebsiella oxytoca* with rhamnose bound. Proteins: Structure, Function, and Bioinformatics.

[16] Romero C, Manjón A, Bastida J, Iborra J (1985). A method for assaying the rhamnosidase activity of naringinase. Analytical biochemistry.

[17] Miyata T (2005). Cloning, sequence analysis, and expression of the gene encoding *Sphingomonas paucimobilis* FP2001 α-L-rhamnosidase. Current microbiology.

[18] Ndeh D (2017). Complex pectin metabolism by gut bacteria reveals novel catalytic functions. nature.

[19] Pulley GN (1936). Solubility of naringin in water. Industrial & Engineering Chemistry Analytical Edition.

[20] Madden RH (1983). Isolation and characterization of *Clostridium stercorarium* sp. nov., cellulolytic thermophile. International journal of systematic and evolutionary microbiology.

[21] Zhang, X. *et al*. *Petroclostridium xylanilyticum* gen. nov., sp. nov., a xylan-degrading bacterium isolated from an oilfield, and reclassification of clostridial cluster III members into four novel genera in a new Hungateiclostridiaceae fam. *nov. International journal of systematic and evolutionary microbiology* (2018). 10.1099/ijsem.0.002966 30124399

[22] Huber R (1986). *Thermotoga maritima* sp. nov. represents a new genus of unique extremely thermophilic eubacteria growing up to 90 C. Archives of Microbiology.

[23] Jannasch HW, Huber R, Belkin S, Stetter KO (1988). *Thermotoga neapolitana* sp. nov. of the extremely thermophilic, eubacterial genus Thermotoga. Archives of Microbiology.

[24] Svetlichnyi VA (1990). *Anaerocellum thermophilum* gen. nov. sp. An extremely thermophilic cellulolytic eubacterium isolated from hot springs in the valley of geysers. Microbiology.

[25] Yang S-J (2010). Classification of ‘*Anaerocellum thermophilum*’ strain DSM 6725 as Caldicellulosiruptor bescii sp. nov. International journal of systematic and evolutionary microbiology.

[26] Broeker J (2018). The hemicellulose-degrading enzyme system of the thermophilic bacterium *Clostridium stercorarium*. Comparative characterisation and addition of new hemicellulolytic glycoside hydrolases. Biotechnology for Biofuels.

[27] Helbert, W. *et al*. Discovery of novel carbohydrate-active enzymes through the rational exploration of the protein sequences space. *Proceedings of the National Academy of Sciences*, 201815791 (2019). 10.1073/pnas.1815791116 PMC6442616 30850540

[28] Birgisson H (2004). Two new thermostable α-L-rhamnosidases from a novel thermophilic bacterium. Enzyme and Microbial Technology.

[29] Duffaud GD, McCutchen CM, Leduc P, Parker KN, Kelly RM (1997). Purification and characterization of extremely thermostable beta-mannanase, beta-mannosidase, and alpha-galactosidase from the hyperthermophilic eubacterium *Thermotoga neapolitana* 5068. Applied and environmental microbiology.

[30] Yadav V, Yadav PK, Yadav S, Yadav KDS (2010). α-L-Rhamnosidase. A review. Process Biochemistry.

[31] Park S-Y, Kim J-H, Kim D-H (2005). Purification and Characterization of Quercitrin-Hydrolyzing α-L-Rhamnosidase from Fusobacterium K-60, a Human Intestinal Bacterium. Journal of microbiology and biotechnology.

[32] Hashimoto W, Miyake O, Nankai H, Murata K (2003). Molecular identification of an α-L-rhamnosidase from *Bacillus* sp. strain GL1 as an enzyme involved in complete metabolism of gellan. Archives of biochemistry and biophysics.

[33] Haupt LJ (2015). The reliability of estimating Ki values for direct, reversible inhibition of cytochrome P450 enzymes from corresponding IC50 values. A retrospective analysis of 343 experiments. Drug Metabolism and Disposition.

[34] Mensitieri F (2018). Structural and functional insights into RHA-P, a bacterial GH106 α-L-rhamnosidase from *Novosphingobium* sp. PP1Y. Archives of biochemistry and biophysics.

[35] Coolbear T, Whittaker JM, Daniel RM (1992). The effect of metal ions on the activity and thermostability of the extracellular proteinase from a thermophilic *Bacillus*, strain EA. 1. Biochemical Journal.

[36] Lee D-W (2005). A thermodynamic study of mesophilic, thermophilic, and hyperthermophilic l-arabinose isomerases. The effects of divalent metal ions on protein stability at elevated temperatures. FEBS letters.

[37] Speciale G, Thompson AJ, Davies GJ, Williams SJ (2014). Dissecting conformational contributions to glycosidase catalysis and inhibition. Current opinion in structural biology.

[38] Zhu Y (2010). Mechanistic insights into a Ca2+-dependent family of α-mannosidases in a human gut symbiont. Nature chemical biology.

[39] Puri M (2012). Updates on naringinase. Structural and biotechnological aspects. Applied microbiology and biotechnology.

[40] Kaur A, Singh S, Singh RS, Schwarz WH, Puri M (2010). Hydrolysis of citrus peel naringin by recombinant α-L-rhamnosidase from *Clostridium stercorarium*. Journal of Chemical Technology & Biotechnology.

[41] De Lise F (2016). RHA-P. Isolation, expression and characterization of a bacterial α-L-rhamnosidase from *Novosphingobium* sp. PP1Y. Journal of Molecular Catalysis B: Enzymatic.

[42] Rodionov DA (2013). Transcriptional regulation of the carbohydrate utilization network in Thermotoga maritima. Frontiers in microbiology.

[43] Petersen TN, Brunak S, Heijne Gvon, Nielsen H (2011). SignalP 4.0. Discriminating signal peptides from transmembrane regions. Nature methods.

[44] Pei J, Kim B-H, Grishin NV (2008). PROMALS3D. A tool for multiple protein sequence and structure alignments. Nucleic acids research.

[45] Berman HM (2000). The Protein Data Bank. Nucleic acids research.

[46] Kumar S, Stecher G, Li M, Knyaz C, Tamura K (2018). MEGA X. Molecular evolutionary genetics analysis across computing platforms. Molecular biology and evolution.

[47] Gibson DG (2009). Enzymatic assembly of DNA molecules up to several hundred kilobases. Nature methods.

[48] Studier FW (2005). Protein production by auto-induction in high-density shaking cultures. Protein expression and purification.

[49] Crowe J, Masone BS, Ribbe J (1995). One-step purification of recombinant proteins with the 6xHis tag and Ni-NTA resin. Molecular biotechnology.

[50] Laemmli UK (1970). Cleavage of structural proteins during the assembly of the head of bacteriophage T4. nature.

[51] Artimo P (2012). ExPASy. SIB bioinformatics resource portal. Nucleic acids research.

[52] Mechelke M (2017). HPAEC-PAD for oligosaccharide analysis—novel insights into analyte sensitivity and response stability. Analytical and bioanalytical chemistry.

[53] Saitou N, Nei M (1987). The neighbor-joining method. A new method for reconstructing phylogenetic trees. Molecular biology and evolution.

[54] Felsenstein J (1985). Confidence limits on phylogenies. An approach using the bootstrap. Evolution.

[55] Zuckerkandl, E. & Pauling, L. In *Evolving genes and proteins* (Elsevier1965), pp. 97–166.

[56] Luis AS (2018). Dietary pectic glycans are degraded by coordinated enzyme pathways in human colonic Bacteroides. Nature microbiology.

[57] Michlmayr H (2011). Characterization of two distinct glycosyl hydrolase family 78 α-L-rhamnosidases from Pediococcus acidilactici. Applied and environmental microbiology.

