# Supplementary material for: Biochemical characterisation of four rhamnosidases from thermophilic bacteria of the genera Thermotoga, Caldicellulosiruptor and Thermoclostridium
Source: Sci Rep. 2019 Nov 4;9:15924. doi: 10.1038/s41598-019-52251-0 (PMC6828813; doi:10.1038/s41598-019-52251-0)
Supplement: Supplementary file 1 — Supplementary information [file 41598_2019_52251_MOESM1_ESM.pdf]

Supplementary Information

Biochemical characterisation of four rhamnosidases from thermophilic bacteria of the genera *Thermotoga*, *Caldicellulosiruptor* and *Thermoclostridium*

Melanie Baudrexl[1], Wolfgang H. Schwarz[2], Vladimir V. Zverlov[1,3,*], Wolfgang Liebl[1,*]

[1] Technical University of Munich, Department of Microbiology, Emil-Ramann-Str. 4, 85354, Freising, Germany

[2] Aspratis GmbH, Hübnerstr. 11, 80637 München, Germany

[3] Institute of Molecular Genetics, Russian Academy of Science, Kurchatov Sq. 2, 123182 Moscow, Russia

* To whom correspondence should be addressed: vladimir.zverlov@tum.de, wliebl@wzw.tum.de

Supplementary Tables

Table S1. BLASTp hits with QC > 70 % and ID>20 % to Ts_Ram106B searched against GHnc (as of February 2019)

	Description	Max score	Total score	Query cover	E value	Ident	Accession
1	hypothetical protein Cst_c00400 [*Thermoclostridium stercorarium* subsp. *stercorarium* DSM 8532]	2156	2156	100%	0.0	100%	AGC67072.1
2	hypothetical protein CSTERTH_00185 [*Thermoclostridium stercorarium* subsp. *thermolacticum* DSM 2910]	2156	2156	100%	0.0	100%	ANW97562.1
3	hypothetical protein Clst_0037 *[Thermoclostridium stercorarium* subsp. *stercorarium* DSM 8532]	2156	2156	100%	0.0	100%	AGI38155.1
4	hypothetical protein CSTERLE_00185 [*Thermoclostridium stercorarium* subsp. *leptospartum* DSM 9219]	2155	2155	100%	0.0	99%	ANX00121.1
5	hypothetical protein CDO33_18065 [*Pseudoclostridium thermosuccinogenes*]	1021	1021	100%	0.0	49%	AUS98191.1
6	alpha-L-rhamnosidase [*Defluviitoga tunisiensis*]	1012	1012	100%	0.0	47%	CEP78513.1
7	hypothetical protein LF65_01629 [*Clostridium beijerinckii*]	1009	1009	100%	0.0	47%	AJG98234.1
8	hypothetical protein C5Q97_18075 [*Victivallales bacterium* CCUG 44730]	902	902	99%	0.0	44%	AVM46512.1
9	conserved hypothetical protein [*Treponema azotonutricium* ZAS-9]	897	897	99%	0.0	44%	AEF82861.1
10	hypothetical protein C5Q97_01805 [*Victivallales bacterium* CCUG 44730]	800	800	98%	0.0	40%	AVM43497.1
11	hypothetical protein C5Q97_19140 [*Victivallales bacterium* CCUG 44730]	537	537	95%	2,00E-176	33%	AVM46711.1
12	hypothetical protein PSMK_17850 [*Phycisphaera mikurensis* NBRC 102666]	525	525	98%	1,00E-171	32%	BAM03944.1
13	hypothetical protein VN24_06320 [*Paenibacillus beijingensis*]	518	518	98%	4,00E-169	31%	AJY74264.1
14	hypothetical protein C5Q97_09920 [*Victivallales bacterium* CCUG 44730]	487	487	99%	1,00E-156	33%	AVM44996.1
15	hypothetical protein Mahau_0169 [*Mahella australiensis* 50-1 BON]	481	481	96%	3,00E-155	31%	AEE95392.1
16	hypothetical protein R70331_07585 [*Paenibacillus* sp. FSL R7-0331]	471	471	98%	2,00E-151	31%	AIQ51388.1
17	conserved hypothetical protein [*Dictyoglomus thermophilum* H-6-12]	464	464	97%	6,00E-149	31%	ACI18379.1
18	putative alpha-L-rhamnosidase family 106 [*Monoglobus pectinilyticus*]	462	462	99%	6,00E-148	31%	AUO20156.1
19	conserved hypothetical protein [*Dictyoglomus turgidum* DSM 6724]	448	448	97%	8,00E-143	32%	ACK41496.1
20	hypothetical protein Back11_07780 [*Paenibacillus baekrokdamisoli*]	425	425	97%	7,00E-134	30%	BBH19433.1
21	conserved hypothetical protein [*Paenibacillus* sp. Y412MC10]	425	425	97%	9,00E-134	30%	ACX65274.1
22	glycoside hydrolase [*Thermotoga* sp. RQ7]	371	414	70%	4,00E-114	37%	AJG41568.1
23	**Glycoside hydrolase family 2, sugar binding [*Thermotoga neapolitana* DSM 4359]**	**368**	**412**	**70%**	**4,00E-113**	**36%**	**ACM23671.1**
24	hypothetical protein B2K_39690 [*Paenibacillus mucilaginosus* K02]	412	412	98%	1,00E-128	29%	AGN70731.1
25	hypothetical protein PM3016_4138 [*Paenibacillus mucilaginosus* 3016]	411	411	98%	4,00E-128	29%	AFC30916.1
26	hypothetical protein KNP414_04730 [*Paenibacillus mucilaginosus* KNP414]	409	409	98%	2,00E-127	29%	AEI43260.1
27	hypothetical protein CELL2_08695 [*Thermotoga* sp. Cell2]	393	393	95%	3,00E-122	29%	AIY88972.1
28	glycoside hydrolase family 2 sugar binding [*Thermotoga* sp. RQ2]	390	390	95%	3,00E-121	28%	ACB10076.1
29	glycoside hydrolase family 2, sugar binding [*Thermotoga petrophila* RKU-1]	389	389	95%	7,00E-121	28%	ABQ47675.1
30	hypothetical protein T2812B_08620 [*Thermotoga* sp. 2812B]	388	388	95%	2,00E-120	29%	AIY87247.1
31	**putative alpha-L-rhamnosidase [*Thermotoga maritima* MSB8]**	**388**	**388**	**95%**	**2,00E-120**	**28%**	**AGL50002.1**
32	hypothetical protein TM_1074 [*Thermotoga maritima* MSB8]	388	388	95%	2,00E-120	28%	AAD36151.1
33	glycoside hydrolase [*Thermotoga maritima* MSB8]	388	388	95%	2,00E-120	28%	AHD19018.1
34	glycoside hydrolase [*Thermotoga maritima* MSB8]	388	388	95%	2,00E-120	28%	AKE28850.1
35	glycoside hydrolase [*Thermotoga maritima*]	388	388	95%	2,00E-120	28%	AKE30723.1

	Description	Max score	Total score	Query cover	E value	Ident	Accession
36	glycoside hydrolase [*Thermotoga maritima*]	388	388	95%	2,00E-120	28%	AKE26985.1
37	hypothetical protein Mahau_1687 [*Mahella australiensis* 50-1 BON]	387	387	98%	1,00E-119	27%	AEE96868.1
38	glycoside hydrolase family 2, sugar binding protein [*Thermotoga naphthophila* RKU-10]	382	382	95%	4,00E-118	30%	ADA67761.1
39	hypothetical protein Calkr_0115 [*Caldicellulosiruptor kristjanssonii* I77R1B]	381	381	98%	8,00E-118	29%	ADQ39690.1
40	hypothetical protein Calkro_0280 [*Caldicellulosiruptor kronotskyensis* 2002]	380	380	98%	3,00E-117	30%	ADQ45191.1
41	hypothetical protein Calow_2157 [*Caldicellulosiruptor owensensis* OL]	380	380	97%	2,00E-117	29%	ADQ05665.1
42	hypothetical protein COB47_2196 [*Caldicellulosiruptor obsidiansis* OB47]	376	376	97%	6,00E-116	28%	ADL43440.1
43	**conserved hypothetical protein [*Caldicellulosiruptor bescii* DSM 6725]**	**373**	**373**	**98%**	**9,00E-115**	**28%**	**ACM61646.1**
44	conserved hypothetical protein [*Caldicellulosiruptor hydrothermalis* 108]	372	372	98%	1,00E-114	29%	ADQ06017.1
45	hypothetical protein Csac_0424 [*Caldicellulosiruptor saccharolyticus* DSM 8903]	369	369	98%	3,00E-113	28%	ABP66063.1
46	hypothetical protein PGRAT_09170 [*Paenibacillus graminis*]	298	363	84%	6,00E-87	31%	AIQ67784.1
47	hypothetical protein PRIO_1914 [*Paenibacillus riograndensis* SBR5]	290	359	84%	2,00E-84	30%	CQR54324.1
48	hypothetical protein Thexy_2082 [*Thermoanaerobacterium xylanolyticum* LX-11]	359	359	98%	9,00E-110	29%	AEF18092.1
49	hypothetical protein AR543_15890 [*Paenibacillus bovis*]	353	353	96%	2,00E-106	27%	ANF97336.1
50	putative protein {ECO:0000313|EMBL:EHB92522,1} [*Petrimonas mucosa*]	346	346	96%	1,00E-104	28%	SCM56672.1
51	hypothetical protein R70723_08710 [*Paenibacillus* sp. FSL R7-0273]	332	332	98%	1,00E-99	25%	AIQ45953.1
52	hypothetical protein GYMC10_2732 [*Paenibacillus* sp. Y412MC10]	330	330	96%	9,00E-99	25%	ACX65003.1
53	Glycosyl hydrolase family 2, sugar binding domain protein [*Petrimonas* sp. IBARAKI]	264	326	73%	1,00E-75	31%	BBA15990.1
54	Glycosyl hydrolase family 2, sugar binding domain protein [*Petrimonas* sp. IBARAKI]	264	326	73%	1,00E-75	31%	BBD46126.1
55	hypothetical protein Mahau_1791 [*Mahella australiensis* 50-1 BON]	325	325	95%	4,00E-97	26%	AEE96972.1
56	hypothetical protein H70357_09110 [*Paenibacillus* sp. FSL H7-0357]	322	322	98%	1,00E-95	25%	AIQ16803.1
57	**hypothetical protein [*Niabella aurantiaca*]**	238	315	75%	7,00E-67	29%	WP_018627535.1
58	hypothetical protein R50912_08660 [*Paenibacillus* sp. FSL R5-0912]	315	315	98%	1,00E-93	25%	AIQ40096.1
59	hypothetical protein PBOR_09520 [*Paenibacillus borealis*]	308	308	98%	4,00E-91	25%	AIQ57144.1
60	hypothetical protein DLD77_09830 [*Chitinophaga* sp. T22]	236	306	73%	3,00E-66	28%	AWO01974.1
61	hypothetical protein Phep_2555 [*Pedobacter heparinus* DSM 2366]	295	295	98%	4,00E-86	25%	ACU04759.1
62	putative protein {ECO:0000313|EMBL:EKN18280,1} [*Petrimonas mucosa*]	292	292	98%	2,00E-85	27%	SCM58509.1
63	hypothetical protein C5Q97_14085 [*Victivallales bacterium* CCUG 44730]	286	286	96%	3,00E-83	25%	AVM45769.1
64	beta-galactosidase [*Paenibacillus ihbetae*]	226	253	79%	1,00E-62	28%	ANY71138.1
65	hypothetical protein GYMC10_2965 [*Paenibacillus* sp. Y412MC10]	221	252	82%	6,00E-61	26%	ACX65235.1
66	beta-galactosidase [*Paenibacillus lautus*]	213	249	82%	1,00E-58	25%	AYB44481.1
67	glycoside hydrolase family 2 sugar binding [*Caldicellulosiruptor bescii* DSM 6725]	233	233	90%	5,00E-65	25%	ACM61153.1
68	glycoside hydrolase family 2 sugar binding protein [*Caldicellulosiruptor kronotskyensis* 2002]	232	232	92%	1,00E-64	24%	ADQ45454.1
69	hypothetical protein BS614_27620 [*Paenibacillus xylanexedens*]	197	231	80%	2,00E-53	27%	APO47442.1
70	glycoside hydrolase family 2 sugar binding [*Caldicellulosiruptor obsidiansis* OB47]	226	226	92%	1,00E-62	24%	ADL43128.1
71	hypothetical protein SAMN05660242_0943 [*Thermoanaerobacterium* sp. RBIITD]	194	221	71%	2,00E-52	25%	SNX53402.1
72	glycoside hydrolase family 2 sugar binding protein [*Caldicellulosiruptor owensensis* OL]	215	215	92%	2,00E-59	24%	ADQ05304.1

Table S2. Quantification of produced rhamnose by HPAEC-PAD. Samples were either H_2O as a blank, rhamnose standards for calibration or reactions containing 10 % naringin and 5 nM Tm_Ram106B and the corresponding controls incubated for the indicated times (inc. time). The enzyme-only control which does not change in its rhamnose content is shown after 160 min.

inj. no.	sample	inc. time min	Type	desired calibration conc. of rhamnose (mg/l)	Rel. Area %	Area nC*min	Height nC	Width (50%) min	rhamnose peak retention time min	rhamnose conc. calculated by chromeleon mg/l	rhamnose conc. in sample mM	rhamnose conc. in sample corrected by BLANK (inj.no. 8) mM	rhamnose conc. in sample corr. by enzyme-only control and substrate-only controls mM	reaction velocity µM/min	specific activity µmol/(min*mg)
1			Check Standard		n.a.	n.a.	n.a.	n.a.	n.a.	n.a.					
2			Check Standard		n.a.	n.a.	n.a.	n.a.	n.a.	n.a.					
3			Check Standard		0.03	0.03	0.09	0.33	4.40	n.a.					
4	500 mg/l rhamnose		Calibration Standard	50.00	47.66	103.08	718.86	0.13	4.43	49.99	3.045	3.042			
5	250 mg/l rhamnose		Calibration Standard	25.00	32.69	55.74	405.48	0.12	4.40	25.07	1.527	1.525			
6	100 mg/l rhamnose		Calibration Standard	10.00	16.71	22.93	169.89	0.12	4.38	9.87	0.601	0.599			
7	50 mg/l rhamnose		Calibration Standard	5.00	11.28	11.98	90.78	0.12	4.37	5.08	0.310	0.307			
8	H20		Blank		0.10	0.13	0.37	0.31	4.42	0.04	0.002	0.000			
9	10 mg/l rhamnose		Calibration Standard	1.00	3.29	2.56	18.90	0.12	4.37	1.06	0.065	0.062			
10	5 mg/l rhamnose		Calibration Standard	0.50	0.50	1.19	8.40	0.12	4.35	0.48	0.029	0.027			
11	30 mg/l rhamnose		Calibration Standard	3.00	7.08	7.02	52.56	0.12	4.35	2.96	0.180	0.178			
12	-enzyme +Naringin	20	reaction		1.10	1.39	9.62	0.13	4.30	0.57	0.035	0.032			
13	+Tm_Ram106B +Naringin	20	reaction		2.35	3.46	20.69	0.13	4.30	1.44	0.088	0.085	0.011	0.539	0.925
14	100 mg/l rhamnose		Calibration Standard	10.00	14.45	21.86	168.00	0.12	4.30	9.39	0.572	0.570			
15	-enzyme +Naringin	40	reaction		1.01	1.40	9.68	0.13	4.28	0.57	0.035	0.032			
16	+Tm_Ram106B +Naringin	40	reaction		4.52	5.51	35.27	0.13	4.23	2.31	0.141	0.138	0.063	1.579	2.708
17	-enzyme +Naringin	80	reaction		0.05	0.05	0.50	n.a.	4.47	0.01					
18	+Tm_Ram106B +Naringin	80	reaction		4.91	7.19	49.36	0.12	4.22	3.03	0.184	0.182	0.107	1.336	2.292
19	+Tm_Ram106B -Substrate	160	reaction		0.98	1.80	4.01	0.33	4.13	0.74	0.045	0.043			
20	-enzyme +Naringin	160	reaction		0.67	1.46	10.60	0.12	4.22	0.60	0.037	0.034			
21	+Tm_RamNB +Naringin	160	reaction		2.81	6.74	47.98	0.12	4.18	2.84	0.173	0.170	0.094	0.585	1.003
22	300 mg/l rhamnose		Calibration Standard	30.00	23.43	62.56	479.17	0.12	4.20	28.41	1.731	1.728			

Table S3. Information about Proteins used for the structure based alignment by Promal3DS (suppl. Fig. S1) in aligned order, which was then used to generate a neighbour joining tree (Fig. 1)

Accession number(s)	PDB	GH	protein name(s)	Organism	Reference
AAO76108.1	**3cih_chainA_p005**	GH78	BT_1001	*Bacteroides thetaiotaomicron* VPI-5482	
WP_008765775.1, AAO76093.1,	**5mqm_chainA_p001**	GH106	BT_0986	*Bacteroides thetaiotaomicron* VPI-5482	Ndeh et al. 2017
BAC68538.1	**3w5n_chainA_p002**	GH78	SaRha78A	Streptomyces avermitilis MA-4680 = NBRC 14893	Fujimoto et al. 2013
AAO76120.1		GH78	BT1013	*Bacteroides thetaiotaomicron* VPI-5482	
CCA90848.1		GH106	RHA-P, PP1Y_Mpl10172	*Novosphingobium* sp. PP1Y	
BAD12237.1		GH106	RhaM	*Sphingomonas paucimobilis* FP2001 / JCM 10661	
CDF79921.1		GH78	BN863_22090	*Formosa agariphila* KMM 3901	
CDF79916.1, WP_084817526.1		GH106	BN863_22040	*Formosa agariphila* KMM 3901	
BAB62315.1	**2okx_chainA_p004**	GH78	RhaB	*Bacillus* sp. GL1	Cui et al. 2007
WP_018627535.1		GH106	B160DRAFT_04058	*Niabella aurantiaca*	Helbert et al. 2019
ACT02314.1		GH106	Pjdr2_3683	*Paenibacillus* sp. JDR-2	Helbert et al. 2019
AEX05711.1	**4xhc_chainA_p003**	GH78	KoRha	*Klebsiella michiganensis* KCTC 1686	O'Neill et al. 2015
NP_813056.1		GH106	BT_4145	*Bacteroides thetaiotaomicron* VPI-5482	Luis et al. 2018
CAB53341.1		GH78	RamA	*Thermoclostridium stercorarium* NCIB 11754	
AAV43293.1		GH78	RamA	*Lactobacillus acidophilus* NCFM	
ACM23671.1		GH106	CTN_1495	*Thermotoga neapolitana* DSM 4359, (homolog to Tn_Ram106B of this study from *Tt. neapolitana* Z2706-MC24)	this study
ACM61646.1		GH106	Cb_Ram106B, Athe_2581	*Caldicellulosiruptor bescii* DSM 6725)	this study
AGL50002.1		GH106	Tm_Ram106B, Tmari_1078	*Thermotoga maritima* MSB8	this study
AGC67072.1		GH106	Ts_Ram106B, Cst_c00400	*Thermoclostridium stercorarium* subsp. *stercorarium* DSM 8532	this study
CAA72194.1		GH13_36	AmyA	*Thermotoga maritima* MSB8	

Table S4 Kinetics and inhibition parameters of Tm_Ram106B, Tn_Ram106B, Cb_Ram106B and Ts_Ram106B determined by analyzing [substrate] vs. velocity curves (Figure S8) created as described in material and methods using GraphPad Prism 7 and models as indicated. Computed standard error in parenthesis.

		**Tm_Ram106B**	**Tn_Ram106B**	**Cb_Ram106B**	**Ts_Ram106B**
Kinetic model, inhibition model fitted to [substrate] vs. velocity curves		Michaelis Menten, Competitive Inhibition	Michaelis Menten, Mixed Model Inhibition	Allosteric sigmoidal, -	Michaelis Menten, Mixed Model Inhibition
parameters determined by best fitting kinetic model	$\mathbf{K_{m}}$ in mM at [I]=0 mM	**1.94** (0.20)	**1.47** (0.14	-	**1.90** (0.18)
	$\mathbf{K_{prime}}$ in mM, $\mathbf{K_{half}}$ **Hill-slope**	-	-	**1.29** (0.23), **1.17** (0.14), **1.64** (0.20)	-
	$\mathbf{V_{max}}$ (U/mg) at [I]=0 mM	**112.40** (5.20)	**76.64** (3.18)	**18.40** (1.27)	**7.06** (0.23)
	R^2	0.99	0.99	0.99	0.99
parameters determined by Michaelis Menten-model	$\mathbf{k_{cat}}$ in 1/s	**217.8** (10.1)	**146.8** (6.1)	ca. **51.9** (6.0)	**14.3** (0.5)
	$\mathbf{K_{m}}$ in mM	1.94 (0.19)	1.47 (0.14)	2.46 (0.55)	1.90 (0.18)
	$\mathbf{V_{max}}$ in µmol/s	2.81E-05	7.66E-02	1.34E-02	3.53E-05
	R^2	0.99	0.99	0.98	0.99
parameters determined by inhibition model	$\mathbf{K_{m}}$ in mM	1.81 (0.12)	1.24 (0.09)	-	1.95 (0.13)
	$\mathbf{V_{max}}$ in U/mg	109.2 (3.2)	72.1 (2.2)	-	7.2 (0.2)
	$\boldsymbol{\alpha}$	-	**1.40** (0.44)	-	**2.56** (0.63)
	$\mathbf{K_{i}}$ in mM	**2.37** (0.13)	**5.50** (1.01)	-	**4.94** (0.75)
	R^2 (global shared)	0.99	0.99	-	0.98
determined by fitting [I] vs. relative activity using specific activities at K_m or K_{prime} interpolated from curves in Fig. S8	$\mathbf{IC_{50}}$ in mM (at K_m or K_{prime} from kinetic model)	**4.9** (at 1.9 mM *p*NPR)	**6.5** (at 1.5 mM *p*NPR)	**5.5** (at 1.3 mM *p*NPR)	**8.3** (at 1.9 mM *p*NPR)

Supplementary Figures

```
Conservation:                                                                                        5
3cih_chainA_p005                --------------------------------------------------------------------
WP_008765775.1_GH106_BT_0     1 MKSRL------KQQ-----------IFA--------ISLLACTAISP------ANA-LQTHLLREQFQNP   38
5mqm_chainA_p001              1 ---------------------------------------------------------LLREQFQNP    9
BAC68538.1_GH78_SaRha78A_     1 MSA-------------------------------LRVTSPSVEY--------------------VQRP   17
3w5n_chainA_p002              1 --A-------------------------------LRVTSPSVEY--------------------VQRP   15
AAO76120.1_GH78_putative_     1 MNKKILI--LSFLS----------VLFLAAGPIRAAIDVTNLRTEQ--------------------LKNP   38
CCA90848.1_GH106_RHA-P__N     1 MPRLSL-----RIV----------LCLAT------ALSTLPVHAESRDDAAEVAPSTRPEPSLEQAFKDP   49
BAD12237.1_GH106_alpha-L-     1 MIRKPL-----RLA----------ALLAC------------------AASF----AVPSWADE--LADGFHNP   34
CDF79921.1_GH78_alpha-L-r     1 MSCE-------------------NSSVL------QEAKHLTISEG---------------------FKNP   24
WP_084817526.1_GH106_hypo     1 MPRFL------KYI----------LGLFL-------ISISAFGQNL----------VPEVTELESGFNSP   37
BAB62315.1_GH78_rhamnosid     1 MAGRNWN---ASWI---------------------------------------------------WGGQ   15
2okx_chainA_p004              1 --GRNWN---ASWI---------------------------------------------------WGGQ   13
WP_018627535.1_GH106_dist     1 MIFKTLFPFFKKHKQRSSPSPGWVLLFPSF-----FISAA-VFAQSLK----KPPAPIPFSTISRNFYDP   60
ACT02314.1_GH106_dist._gl     1 MSNRL-------------------------------------------------------KEVLEGR   12
AEX05711.1_GH78_KoRha__Kl     1 MS--------------------------------------------------------------------    2
4xhc_chainA_p003                --------------------------------------------------------------------
NP_813056.1_GH106_hypothe     1 MNKKQL-----------------------------ILLCMLAAGG--------------SIQAQQWPDA   26
CAB53341.1_GH78_Ram78A_al     1 M---------------------------------MRVYNLKTNR--------------------IKNP   15
AAV43293.1_GH78_alfa-l-rh     1 ----------------------------------MKITNILVNQ--------------------MEHP   14
ACM23671.1*GH106Glycoside     1 MNL---------------------------------------------------------KDLENP    9
ACM61646.1*GH106conserved     1 MLDL--------------------------------------------------------EKLKNP   10
AGL50002.1*GH106putative_     1 MNL---------------------------------------------------------KDLENP    9
AGC67072.1*GH106hypotheti     1 MF----------------------------------------FKN---------SREEFD--ESLFRNP   18
CAA72194.1_GH13_alpha-amy     1 MKKPFLL---LAFV----------FLL------------FLTSCF--------QTSMSQS--LVSSNPH   34
Consensus_aa:                   ..................................................................h.s.
Consensus_ss:
```

```
Conservation:                                     5
3cih_chainA_p005              1 ------------------QTWIWYPG--------------------DYEIWL--G--NQMNNRR----   22
WP_008765775.1_GH106_BT_0    39 S------------DEAKPWTFWYWMFG---------AVSKE---GITADLEAMKRAGLGGTYLMPI--KG   82
5mqm_chainA_p001             10 S------------DEAKPWTFWYWMFG---------AVSKE---GITADLEAMKRAGLGGTYLMPI--KG   53
BAC68538.1_GH78_SaRha78A_    18 LG----------LDAAHPRLSWPMASA---------APGRR-----------------QSA---------   42
3w5n_chainA_p002             16 LG----------LDAAHPRLSWPMASA---------APGRR-----------------QSA---------   40
AAO76120.1_GH78_putative_    39 SG----------IDTRQPRLGWRIESD---------EQNVM-----------------QTA---------   63
CCA90848.1_GH106_RHA-P__N    50 P------------SSARPRVWWHWMNG---------NITKD---GIRKDLEWMKRVGIGGLQNFD----A   91
BAD12237.1_GH106_alpha-L-    35 P------------QSARPRVWWHWMNG---------NVTKD---GISKDLDWMQRVGIGGVQNFD----A   76
CDF79921.1_GH78_alpha-L-r    25 LG----------FYDAKPTFSWELPVV---------EGVIS-----------------QSA---------   49
WP_084817526.1_GH106_hypo    38 P------------NQAKARTWWHWISG---------NVSKS---GITKDLEAMKAVGIQEAQLFN----V   79
BAB62315.1_GH78_rhamnosid    16 E------------ESPRNEWRCFRGSF---------DAPAS---VEGPA--MLHITADSRYVLFVNGEQV   59
2okx_chainA_p004             14 E------------ESPRNEWRCFRGSF---------DAPAS---VEGPA--MLHITADSRYVLFVNGEQV   57
WP_018627535.1_GH106_dist    61 G------------KEFQTAPLWVWNT----------RVTTG---MIDSMLQEFKANAFGGVFIHPR---P  102
ACT02314.1_GH106_dist._gl    13 E------------HNYI--LPFLWQHG----------EDEH---RIREEMARVHEAGIGAVCVEAR----   51
AEX05711.1_GH78_KoRha__Kl     3 ------------------QAIQHNS-----------QVMMTRHPNFLRTAEALRP---------------   28
4xhc_chainA_p003              1 -------------------------------------VMMTRHPNFLRTAEALRP---------------   18
NP_813056.1_GH106_hypothe    27 P------------AEARPGTRWWWLGS---------AVDEK---NLTYNLEEYARAGMGAVEITPI---Y   69
CAB53341.1_GH78_Ram78A_al    16 MG----------FVINKPKLSWLVESD----------TAKH-----------------QVA---------   39
AAV43293.1_GH78_alfa-l-rh    15 LG----------FDLSNLRITFELTEM---------ENIIG-----------------NVY---------   39
ACM23671.1*GH106Glycoside    10 G------------VWYRPAPFWSWND----------KLCEE---ELLRQIDEMCEKGYGGFFMHSR---V   51
ACM61646.1*GH106conserved    11 D------------NFYRCAPFWSWND----------NLKEE---ELLRQITEMHQKGYGGFFMHSR---V   52
AGL50002.1*GH106putative_    10 G------------VWYRPAPFWSWND----------KLCEE---ELLRQIDEMYEKGYGGFFMHSR---V   51
AGC67072.1*GH106hypotheti    19 T------------SEYRCAPFWAWNK----------KLDWE---DMERHIEIFKEMGMGGFHIHSR---I   60
CAA72194.1_GH13_alpha-amy    35 SNSTNTDGTSSNLEEVKYPVVYEIFIRSFYDRDGNGVGDLN---GVSQKVDYLKELGVDAVWFMPF---N   98
Consensus_aa:                   ................ps...@.h.............s.p..................sh........
Consensus_ss:                                   eeeee                hh    hhhhhhhhhhh     eeee
```

```
Conservation:
3cih_chainA_p005             23 -----------------TER-----------------G--AFFPP-----------------------   31
WP_008765775.1_GH106_BT_0    83 IKEGPQ-YNGKAQQLT-PEW---WEMVRFSMEEADRLG--LKLGMHI-----------------------  122
5mqm_chainA_p001             54 IKEGPQ-YNGKAQQLT-PEW---WEMVRFSMEEADRLG--LKLGMHI-----------------------   93
BAC68538.1_GH78_SaRha78A_    43 ------------------------------------------YQVRVAS----------------------   49
3w5n_chainA_p002             41 ------------------------------------------YQVRVAS----------------------   47
AAO76120.1_GH78_putative_    64 ------------------------------------------YHILVAS----------------------   70
CCA90848.1_GH106_RHA-P__N    92 NLQTPQIVDHRLVYMT-PEW---KDAFRFAAHEADRLD--LELAIAA-----------------------  132
BAD12237.1_GH106_alpha-L-    77 NLSTPQIVPERLIYMT-EQW---KDAFRHAVREADARG--LEFAIAV-----------------------  117
CDF79921.1_GH78_alpha-L-r    50 ------------------------------------------YQIVVAS----------------------   56
WP_084817526.1_GH106_hypo    80 DLGFP---AGPVDYLS-EDW---LDLFHFSALEAKRIG--LELTFHN-----------------------  117
BAB62315.1_GH78_rhamnosid    60 GRG------PVRSW---PKE---QFYDSYDIGGQLRPGVRNTIAVLVLHFGVSNFYYLRGRGGLIAEIEA  117
2okx_chainA_p004             58 GRG------PVRSW---PKE---QFYDSYDIGGQLRPGVRNTIAVLVLHFGVSNFYYLRGRGGLIAEIEA  115
WP_018627535.1_GH106_dist   103 GLI--------TEYLG-DEW---LSLYKHAVQKAAALD--MHIWIYD-----------------------  135
ACT02314.1_GH106_dist._gl    52 ---------PHPDFLG-PKW---WADMDIIMDEARQRG--MKVWLLD-----------------------   83
AEX05711.1_GH78_KoRha__Kl    29 -------------ALSRQAHP-PIAVVEAHADAAALFG--W-----------------------------   53
4xhc_chainA_p003             19 -------------ALSRQAHP-PIAVVEAHADAAALFG--W-----------------------------   43
NP_813056.1_GH106_hypothe    70 GVQGND--ANDIQFLS-PRW---MEVLKHTQAEGKRTG--IEIDMNT-----------------------  108
CAB53341.1_GH78_Ram78A_al    40 ------------------------------------------AQVEISA----------------------   46
AAV43293.1_GH78_alfa-l-rh    40 ------------------------------------------KNISVGK----------------------   46
ACM23671.1*GH106Glycoside    52 GLV--------TEYLS-DEW---MALVKRCAEHARKLG--MLAWLYD-----------------------   84
ACM61646.1*GH106conserved    53 GLV--------TEYLS-EEW---LNLVKKCIEHAKKLN--MLAWLYD-----------------------   85
AGL50002.1*GH106putative_    52 GLV--------TEYLS-EEW---MRLVRSCAEHARKLG--MLAWLYD-----------------------   84
AGC67072.1*GH106hypotheti    61 GLN--------TPYLS-DEF---LDYVKKCNAKAKELG--MLTWLYD-----------------------   93
CAA72194.1_GH13_alpha-amy    99 EAVSYH-GYDITDYYNVEKDYGTMEDLENMIQVLHENG--IKVIMDL-----------------------  142
Consensus_aa:                   ..........................................h.h.h.s......................
Consensus_ss:                                         hhhhhhhhhhhhh      eeeee
```

```
Conservation:
3cih_chainA_p005              32  -----------FWKTD-----SHYVVVE------------------------------------------   43
WP_008765775.1_GH106_BT_0    123  --------CDGFAL--AGGPWMTPKESMQKIVWSDTIVDGGK----------------------------  154
5mqm_chainA_p001              94  --------CDGFAL--AGGPWMTPKESMQKIVWSDTIVDGGK----------------------------  125
BAC68538.1_GH78_SaRha78A_     50  --------SAAGLSH--------------PDVWDSGKVVSDDSVLVPYA--GPPLKPRTRYFWSVRVWDA   95
3w5n_chainA_p002              48  --------SAAGLSH--------------PDVWDSGKVVSDDSVLVPYA--GPPLKPRTRYFWSVRVWDA   93
AAO76120.1_GH78_putative_     71  --------SPELLA--QG----------KGDMWDSGKVETDASQWITYQ--GETLKRNAPYFWKVKVYTN  118
CCA90848.1_GH106_RHA-P__N    133  --------SPGWSE--TGGPWVKPQDGLKKLVWSETTLAGGQRFVGRLASPPGTTGPFQTLHPPVTIEEI  192
BAD12237.1_GH106_alpha-L-    118  --------SPGWSE--TGGPWVKPEDAMKKLVWAEADLRGGQRLKGALPAPPSITGPFQSAQFH---EAL  174
CDF79921.1_GH78_alpha-L-r     57  --------SPDLLPN-------------NPDLWDSNKQSSSQSVWINYE--GKPLVSRQKVFWQVKYWNQ  103
WP_084817526.1_GH106_hypo    118  --------TAGWSS--SGGPWISPEYAM------------------------------------------  135
BAB62315.1_GH78_rhamnosid    118  DGRTLAATDAAWRTERLGGQ--------------------------------------------------  137
2okx_chainA_p004             116  DGRTLAATDAAWRTERLGGQ--------------------------------------------------  135
WP_018627535.1_GH106_dist    136  --------ENSYPTGFAGGLV-------------------------------------------------  148
ACT02314.1_GH106_dist._gl     84  --------DDHFPTGHAAGK--------------------------------------------------   95
AEX05711.1_GH78_KoRha__Kl         ----------------------------------------------------------------------
4xhc_chainA_p003                  ----------------------------------------------------------------------
NP_813056.1_GH106_hypothe    109  --------GTGWPF---GGPEVSIE---------------------------------------------  122
CAB53341.1_GH78_Ram78A_al     47  --------DINF----------------ENIIFDSGKRTDID-SISYSP--QVELKPRTRYYWRVRVWGD   89
AAV43293.1_GH78_alfa-l-rh     47  --------VESE----------------QPIYFEPDELYENNAFKINM-----ELEPRTKYWVKIGVRND   87
ACM23671.1*GH106Glycoside     85  --------EDKWPSGFAGGI--------------------------------------------------   96
ACM61646.1*GH106conserved     86  --------EDKWPSGFAGGA--------------------------------------------------   97
AGL50002.1*GH106putative_     85  --------EDKWPSGFAGGI--------------------------------------------------   96
AGC67072.1*GH106hypotheti     94  --------EDKWPSGFGGGF--------------------------------------------------  105
CAA72194.1_GH13_alpha-amy    143  --------VINHTS--DEH---------------------------------------------------  151
Consensus_aa:                     ........s.h...........................................................
Consensus_ss:


Conservation:
3cih_chainA_p005                  ----------------------------------------------------------------------
WP_008765775.1_GH106_BT_0         ----------------------------------------------------------------------
5mqm_chainA_p001                  ----------------------------------------------------------------------
BAC68538.1_GH78_SaRha78A_     96  DGG----ASEWSAPSWWETGLMGASQWSAKWISAPAPLTEAPSLEGSSWIWFPEGEPANSAPAATRWFRR  161
3w5n_chainA_p002              94  DGG----ASEWSAPSWWETGLMGASQWSAKWISAPAPLTEAPSLEGSSWIWFPEGEPANSAPAATRWFRR  159
AAO76120.1_GH78_putative_    119  KGE-----SDWSSPAFWSMGLFNEADWQGQWIGLDRAAPGD-----------------------------  154
CCA90848.1_GH106_RHA-P__N    193  ISGVPAETGGVSYAGEVGVLAFPVPDIASLPVPRALDGAGNVLAGKALV---------------------  241
BAD12237.1_GH106_alpha-L-    175  PGGAEMQTLPAFYR-DARVLAYSLVP-KALPRPAVSEGDGSPVDAAPLL---------------------  221
CDF79921.1_GH78_alpha-L-r    104  DDK----ASNWSPVQNFELGLLNNSDWKAKWIGLPTKEEGVL----------------------------  141
WP_084817526.1_GH106_hypo         ----------------------------------------------------------------------
BAB62315.1_GH78_rhamnosid    138  ---------------------RSNSPRMACQQGFGEVIDARELAEDWALPAFDDGG--------------  172
2okx_chainA_p004             136  ---------------------RSNSPRMACQQGFGEVIDARELAEDWALPAFDDGG--------------  170
WP_018627535.1_GH106_dist         ----------------------------------------------------------------------
ACT02314.1_GH106_dist._gl         ----------------------------------------------------------------------
AEX05711.1_GH78_KoRha__Kl         ----------------------------------------------------------------------
4xhc_chainA_p003                  ----------------------------------------------------------------------
NP_813056.1_GH106_hypothe         ----------------------------------------------------------------------
CAB53341.1_GH78_Ram78A_al     90  DGS-----EAVSEAAWFETSKMDE-PWKAKWITPDF----------------------------------  119
AAV43293.1_GH78_alfa-l-rh     88  N-------EVTSSNTWFETGKMDE-KFYGKWITNKK----------------------------------  115
ACM23671.1*GH106Glycoside         ----------------------------------------------------------------------
ACM61646.1*GH106conserved         ----------------------------------------------------------------------
AGL50002.1*GH106putative_         ----------------------------------------------------------------------
AGC67072.1*GH106hypotheti         ----------------------------------------------------------------------
CAA72194.1_GH13_alpha-amy         ----------------------------------------------------------------------
Consensus_aa:                     ......................................................................
Consensus_ss:


Conservation:
3cih_chainA_p005                  ----------------------------------------------------------------------
WP_008765775.1_GH106_BT_0         ----------------------------------------------------------------------
5mqm_chainA_p001                  ----------------------------------------------------------------------
BAC68538.1_GH78_SaRha78A_    162  TVDLPDDITGATLAISADNVYAVSVDGAEVARTDLEADNEGWRRPAVIDVLDHVHSGNNTLAVSASNASV  231
3w5n_chainA_p002             160  TVDLPDDITGATLAISADNVYAVSVDGAEVARTDLEADNEGWRRPAVIDVLDHVHSGNNTLAVSASNASV  229
AAO76120.1_GH78_putative_         ----------------------------------------------------------------------
CCA90848.1_GH106_RHA-P__N         ----------------------------------------------------------------------
BAD12237.1_GH106_alpha-L-         ----------------------------------------------------------------------
CDF79921.1_GH78_alpha-L-r         ----------------------------------------------------------------------
WP_084817526.1_GH106_hypo         ----------------------------------------------------------------------
BAB62315.1_GH78_rhamnosid         ----------------------------------------------------------------------
2okx_chainA_p004                  ----------------------------------------------------------------------
WP_018627535.1_GH106_dist         ----------------------------------------------------------------------
ACT02314.1_GH106_dist._gl         ----------------------------------------------------------------------
AEX05711.1_GH78_KoRha__Kl         ----------------------------------------------------------------------
4xhc_chainA_p003                  ----------------------------------------------------------------------
NP_813056.1_GH106_hypothe         ----------------------------------------------------------------------
CAB53341.1_GH78_Ram78A_al         ----------------------------------------------------------------------
AAV43293.1_GH78_alfa-l-rh         ----------------------------------------------------------------------
ACM23671.1*GH106Glycoside         ----------------------------------------------------------------------
ACM61646.1*GH106conserved         ----------------------------------------------------------------------
AGL50002.1*GH106putative_         ----------------------------------------------------------------------
AGC67072.1*GH106hypotheti         ----------------------------------------------------------------------
CAA72194.1_GH13_alpha-amy         ----------------------------------------------------------------------
Consensus_aa:                     ......................................................................
Consensus_ss:
“GH106”

Conservation:
3cih_chainA_p005                  ----------------------------------------------------------------------
```

```
WP_008765775.1_GH106_BT_0          ----------------------------------------------------------------------
5mqm_chainA_p001                   ----------------------------------------------------------------------
BAC68538.1_GH78_SaRha78A_     232  GPAGWICVLVLTTASGEKKIFSDASWKSTDHEPADGWREPDFDDSGWPAAKVAAAWGAGPWGRVAPVASA  301
3w5n_chainA_p002              230  GPAGWICVLVLTTASGEKKIFSDASWKSTDHEPADGWREPDFDDSGWPAAKVAAAWGAGPWGRVAPVASA  299
AAO76120.1_GH78_putative_     155  -------------------------------------------------------------SETQWSRLA  163
CCA90848.1_GH106_RHA-P__N     242  ------------------------------------------------------DADIAGGVTLARVDGK  257
BAD12237.1_GH106_alpha-L-     222  ------------------------------------------------------DGDLETVARIGKGDAA  237
CDF79921.1_GH78_alpha-L-r     142  -------------------------------------------------------------GSQDNIIHR  150
WP_084817526.1_GH106_hypo          ----------------------------------------------------------------------
BAB62315.1_GH78_rhamnosid     173  ----------------------------------------------WAQARSIGPAGTAPWTSLVPRDIP  196
2okx_chainA_p004              171  ----------------------------------------------WAQARSIGPAGTAPWTSLVPRDIP  194
WP_018627535.1_GH106_dist     149  -------------------------------------------------------------PDQMPGSYN  157
ACT02314.1_GH106_dist._gl      96  ------------------------------------------------------------------VKQA   99
AEX05711.1_GH78_KoRha__Kl          ----------------------------------------------------------------------
4xhc_chainA_p003                   ----------------------------------------------------------------------
NP_813056.1_GH106_hypothe          ----------------------------------------------------------------------
CAB53341.1_GH78_Ram78A_al     120  ------------------------------------------------------------------DPSV  123
AAV43293.1_GH78_alfa-l-rh     116  -------------------------------------------------------------------DVE  118
ACM23671.1*GH106Glycoside      97  ------------------------------------------------------------------VPLE  100
ACM61646.1*GH106conserved      98  ------------------------------------------------------------------VAFK  101
AGL50002.1*GH106putative_      97  ------------------------------------------------------------------VPLE  100
AGC67072.1*GH106hypotheti     106  ------------------------------------------------------------------VTKD  109
CAA72194.1_GH13_alpha-amy          ----------------------------------------------------------------------
Consensus_aa:                      ......................................................................
Consensus_ss:


Conservation:
3cih_chainA_p005               44  ----FSKVLNLSE----PEEVFIAAE----------GTYNVKLDGKLQFGMPETLLLPAGKHSLNIKVWN   95
WP_008765775.1_GH106_BT_0     155  -----------------------------------------------IKGLHL--------------PQP  163
5mqm_chainA_p001              126  -----------------------------------------------IKGLHL--------------PQP  134
BAC68538.1_GH78_SaRha78A_     302  AN-QLRHEFRLPHKKVSRARLYATAL----------GLYEAHLNGR-RVGRDQ--------------LAP  345
3w5n_chainA_p002              300  AN-QLRHEFRLPHKKVSRARLYATAL----------GLYEAHLNGR-RVGRDQ--------------LAP  343
AAO76120.1_GH78_putative_     164  AR-YLRKEFALKK-EIKRATVHIAGM----------GLYELFINGQ-RIGDQV--------------LAP  206
CCA90848.1_GH106_RHA-P__N     258  AP-LLRLDYQRPV-TVRSATVFVPNVRIPFAGAAFAGTLESSQDGK------------------------  301
BAD12237.1_GH106_alpha-L-     238  RPGTMVLDYGKPV-TVRSASLFVPHARPPFGDPDYRPALEVEQDG-------------------------  281
CDF79921.1_GH78_alpha-L-r     151  PQ-YLRKVFELSN-DVANARLYITAK----------GVFDVAINGE-DVSDDV--------------MPP  193
WP_084817526.1_GH106_hypo          ----------------------------------------------------------------------
BAB62315.1_GH78_rhamnosid     197  F---LTEEKLYP---ASIQSLSRVKA----------PKYAAALDLR-------------------NQMVP  231
2okx_chainA_p004              195  F---LTEEKLYP---ASIQSLSRVKA----------PKYAAALDLR-------------------NQMVP  229
WP_018627535.1_GH106_dist     158  QG-QMLQ----------------LFR--------------------------------------------  166
ACT02314.1_GH106_dist._gl     100  PE-ELHRKFL--------AERYIDTVGPAN--GTSLLLDTLLMNG----------------------LRP  136
AEX05711.1_GH78_KoRha__Kl          ----------------------------------------------------------------------
4xhc_chainA_p003                   ----------------------------------------------------------------------
NP_813056.1_GH106_hypothe          ----------------------------------------------------------------------
CAB53341.1_GH78_Ram78A_al     124  HP-VVFTDFSIER-DVADARAYVCGL----------GLYEMSVNGE-KTGDEY--------------LAP  166
AAV43293.1_GH78_alfa-l-rh     119  NT-LFKKDFELANKQIKSARLYSTTL----------GVYEVDLNGV-KVGNEF--------------LAP  162
ACM23671.1*GH106Glycoside     101  SD--------------------------------------------------------------------  102
ACM61646.1*GH106conserved     102  NP--------------------------------------------------------------------  103
AGL50002.1*GH106putative_     101  KP--------------------------------------------------------------------  102
AGC67072.1*GH106hypotheti     110  HN--------------------------------------------------------------------  111
CAA72194.1_GH13_alpha-amy          ----------------------------------------------------------------------
Consensus_aa:                      ......................................................................
Consensus_ss:                                           e


Conservation:
3cih_chainA_p005               96  QATPPTIYVKGKTVNSDSSWRVTYEDKEWIDESGKASDTSATIYMDAGCWNFDGATQRPSQFSLMREPQQ  165
WP_008765775.1_GH106_BT_0     164  EAYEGFY------------------------------------EDISLF--ALPVKEEAADVMPAQITCA  195
5mqm_chainA_p001              135  EAYEGFY------------------------------------EDISLF--ALPVKEEAADVMPAQITCA  166
BAC68538.1_GH78_SaRha78A_     346  -GWTDYR------------------------------------KRVQYQ--TYDVT-------------S  363
3w5n_chainA_p002              344  -GWTDYR------------------------------------KRVQYQ--TYDVT-------------S  361
AAO76120.1_GH78_putative_     207  -APTDYR------------------------------------KTILYN--TYDVT-------------S  224
CCA90848.1_GH106_RHA-P__N     302  ----TWT------------------------------------PIKALE--LSNVP-------------T  316
BAD12237.1_GH106_alpha-L-     282  ----GWR------------------------------------RIGAFP--LTEVA-------------T  296
CDF79921.1_GH78_alpha-L-r     194  -GYTPYK------------------------------------KRIETI--TYDVT-------------D  211
WP_084817526.1_GH106_hypo     136  -------------------------------------------QTVVYS--EIIVK--------------  146
BAB62315.1_GH78_rhamnosid     232  -ESVNHA------------------------------------NPVSYC--GYVAT-------------I  249
2okx_chainA_p004              230  -ESVNHA------------------------------------NPVSYC--GYVAT-------------I  247
WP_018627535.1_GH106_dist          ----------------------------------------------------------------------
ACT02314.1_GH106_dist._gl     137  -TFSTLE-----------------------------------NAKGKNKLISVAAV-------------R  157
AEX05711.1_GH78_KoRha__Kl          ----------------------------------------------------------------------
4xhc_chainA_p003                   ----------------------------------------------------------------------
NP_813056.1_GH106_hypothe          ----------------------------------------------------------------------
CAB53341.1_GH78_Ram78A_al     167  -GLVAYD------------------------------------KWIPYQ--TYDIT-------------S  184
AAV43293.1_GH78_alfa-l-rh     163  -GFTNYD------------------------------------KIVQLQ--TYDVT-------------K  180
ACM23671.1*GH106Glycoside     103  ------E------------------------------------YRHRFL--VLLKK-------------E  115
ACM61646.1*GH106conserved     104  ------S------------------------------------YRHKFL--VLLKE-------------D  116
AGL50002.1*GH106putative_     103  ------E------------------------------------HRHKYL--TLLKK-------------D  115
AGC67072.1*GH106hypotheti     112  -------------------------------------------FRSRYL--LFSP---------------  121
CAA72194.1_GH13_alpha-amy          ----------------------------------------------------------------------
Consensus_aa:                      ...............................................h....h.h...............
Consensus_ss:                                                                     eee  eee


Conservation:
3cih_chainA_p005              166  PVAKT------------EQPEGGILYDFGKETFGFITLKNLSGKGKIDLYYGESPEEAKDKAYCETLDKL  223
WP_008765775.1_GH106_BT_0     196  NIATG------------NHIDIKKTVN---------------MDDAGV-IRS------------SYPCYI  225
5mqm_chainA_p001              167  NIATG------------NHIDIKKTVN---------------MDDAGV-IRS------------SYPCYI  196
BAC68538.1_GH78_SaRha78A_     364  SVRPG------------ANALAAYVAP---------------GWYAG--NVG------------MFGPH-  391
```

```
3w5n_chainA_p002           362  SVRPG------------ANALAAYVAP---------------GWYAG--NVG------------MFGPH-  389
AAO76120.1_GH78_putative_  225  QLQQ-------------ENAIGVTLGN---------------GRFYT--MRQ------------NYKPYK  252
CCA90848.1_GH106_RHA-P__N  317  TISFAPVEAAHFRLVLNPGQPDAALGS---------------PAPGVA-GND------------LFGAIA  358
BAD12237.1_GH106_alpha-L-  297  TISFAPVTGRRFRLVLAPNDAPRAPGLG-------------EGAPGAI-TMD-----------------V  335
CDF79921.1_GH78_alpha-L-r  212  LIESG------------QNTIGVEVAA---------------GWHSG--RLG------------WMKSYW  240
WP_084817526.1_GH106_hypo  147  ----------------GGKAIKKQLPQPE----------TKLNFYKD--IAV-----------------L  171
BAB62315.1_GH78_rhamnosid  250  LTLET------------SGVVTLGFPT---------------GVRGS--GVW------------VDGVLQ  278
2okx_chainA_p004           248  LTLET------------SGVVTLGFPT---------------GVRGS--GVW------------VDGVLQ  276
WP_018627535.1_GH106_dist       ----------------------------------------------------------------------
ACT02314.1_GH106_dist._gl  158  RDSSDG----------------------------------------------------------------  163
AEX05711.1_GH78_KoRha__Kl       ----------------------------------------------------------------------
4xhc_chainA_p003                ----------------------------------------------------------------------
NP_813056.1_GH106_hypothe       ----------------------------------------------------------------------
CAB53341.1_GH78_Ram78A_al  185  QLKKG------------INTAEFLLGN---------------GWYKG--RYG------------LNRKQP  213
AAV43293.1_GH78_alfa-l-rh  181  LVTKNS-----------NNELVFSVGD---------------GWYKG--NLG------------FDGGQT  210
ACM23671.1*GH106Glycoside  116  QIEP-------------GDEILKRIER-------------------------------------------  129
ACM61646.1*GH106conserved  117  QVEH-------------DDELLSSFVH-------------------------------------------  130
AGL50002.1*GH106putative_  116  QIKP-------------EDEILKKIER-------------------------------------------  129
AGC67072.1*GH106hypotheti  122  ----------------------FYHQS---------------GYYER--HIP--------------RENR  138
CAA72194.1_GH13_alpha-amy  152  ---------------------PWFKDA---------------VENTTS----------------------  163
Consensus_aa:                   ........................h.............................................
Consensus_ss:


Conservation:
3cih_chainA_p005           224  LLEPGQITDLAIRSTSPLHHSDNEY--TLENS-------------------------KAFRYVYITHEPE  266
WP_008765775.1_GH106_BT_0  226  --------------QYEYEQPFTCRNIEIILSGN-----------------------NYQAHR-LKVMAS  257
5mqm_chainA_p001           197  --------------QYEYEQPFTCRNIEIILSGN-----------------------NYQAHR-LKVMAS  228
BAC68538.1_GH78_SaRha78A_  392  ---------------QYGERPALLAQLEVEYADGTS--------------ERITSGPDWRAAS-GPIVSA  431
3w5n_chainA_p002           390  ---------------QYGERPALLAQLEVEYADGTS--------------ERITSGPDWRAAS-GPIVSA  429
AAO76120.1_GH78_putative_  253  --------------IPTFGYPKLRLNLIVEYADGSK--------------ETIATNTSWKLTTEGPVRSN  294
CCA90848.1_GH106_RHA-P__N  359  --------------SKRAGQPIMVGQFELHSDAL---------------------VDRYETKA-GFVMSR  392
BAD12237.1_GH106_alpha-L-  336  -------------FARPQSDTLAIGDFRLLAEAR---------------------IDRFEAKA-GFALVS  370
CDF79921.1_GH78_alpha-L-r  241  ---------------SDTESPKILCQLEVTMKDGSK--------------ASIISDDTWKATTQGPIRIS  281
WP_084817526.1_GH106_hypo  172  -------------AFPKPKQTMKID------------------------------DLDFKSLS-GRIRNH  197
BAB62315.1_GH78_rhamnosid  279  -------------TEWTGVQPERYYSLNLAAGEHLVLVDITSSDHGGSSHFAIDSEAAFTLRSPAGDNGV  335
2okx_chainA_p004           277  -------------TEWTGVQPERYYSLNLAAGEHLVLVDITSSDHGGSSHFAIDSEAAFTLRSPAGDNGV  333
WP_018627535.1_GH106_dist  167  -----------------------------------------------------------SDTVPGN----  173
ACT02314.1_GH106_dist._gl       ----------------------------------------------------------------------
AEX05711.1_GH78_KoRha__Kl       ----------------------------------------------------------------------
4xhc_chainA_p003                ----------------------------------------------------------------------
NP_813056.1_GH106_hypothe  123  ----------------DAASKAIFQTYDIEGGQ------------------EIVQDINVT----------  148
CAB53341.1_GH78_Ram78A_al  214  --------------FRYGNEFALICEIHITYQDGTA--------------DVIYTDTSWKARK-SKVIDS  254
AAV43293.1_GH78_alfa-l-rh  211  --------------NIYGDKKSILAELHVTYTDNSE--------------QVISTDSSWLTTE-GKIIKS  251
ACM23671.1*GH106Glycoside       ----------------------------------------------------------------------
ACM61646.1*GH106conserved       ----------------------------------------------------------------------
AGL50002.1*GH106putative_       ----------------------------------------------------------------------
AGC67072.1*GH106hypotheti  139  --------------LGIDGYLSLIAEYKVVLKNGYL----------------------------------  160
CAA72194.1_GH13_alpha-amy       ----------------------------------------------------------------------
Consensus_aa:                   ......................................................................
Consensus_ss:                                        eeeeeeee


Conservation:
3cih_chainA_p005           267  --------VQIGEVSM-----QYEY------------------------------------LPEEY----  283
WP_008765775.1_GH106_BT_0  258  --------DDGVNYRLVKQLVPARQ---------------------GWQNTDENSTHAIPATTARYFRFY  298
5mqm_chainA_p001           229  --------DDGVNYRLVKQLVPARQ---------------------GWQNTDENSTHAIPATTARYFRFY  269
BAC68538.1_GH78_SaRha78A_  432  ------DLLSGETYDAR----KETA---------------------GWTSPGFDDR------AWLAVRGA  464
3w5n_chainA_p002           430  ------DLLSGETYDAR----KETA---------------------GWTSPGFDDR------AWLAVRGA  462
AAO76120.1_GH78_putative_  295  ------NEYDGEEYDAR----KELG---------------------NWTQTDYDDT------KWMPAERV  327
CCA90848.1_GH106_RHA-P__N  393  ------DYYALV----------------------------------------------------------  398
BAD12237.1_GH106_alpha-L-  371  ------DYNA------------------------------------------------------------  374
CDF79921.1_GH78_alpha-L-r  282  ------EIYDGETYDAH----LEMP---------------------HWTTNSFDDK------NWKAVQAF  314
WP_084817526.1_GH106_hypo  198  ------LLPDTK----------------------------------------------------------  203
BAB62315.1_GH78_rhamnosid  336  PLATIGTFDQSEYIDHRPGRRMQTDHPDYRALPEAAPTAAALEAFASWVKPF-EPS------LYTEENVF  398
2okx_chainA_p004           334  PLATIGTFDQSEYIDHRPGRRMQTDHPDYRALPEAAPTAAALEAFASWVKPF-EPS------LYTEENVF  396
WP_018627535.1_GH106_dist       ----------------------------------------------------------------------
ACT02314.1_GH106_dist._gl       ----------------------------------------------------------------------
AEX05711.1_GH78_KoRha__Kl       ----------------------------------------------------------------------
4xhc_chainA_p003                ----------------------------------------------------------------------
NP_813056.1_GH106_hypothe  149  ----------------------------------------------DKKQQ-------------------  153
CAB53341.1_GH78_Ram78A_al  255  ------GIYDGEIYDDT----FCDD---------------------AVYPV----------------RIA  277
AAV43293.1_GH78_alfa-l-rh  252  ------SIYYGEDIDDT----KDIL---------------------DWSSV----------------VIL  274
ACM23671.1*GH106Glycoside       ----------------------------------------------------------------------
ACM61646.1*GH106conserved       ----------------------------------------------------------------------
AGL50002.1*GH106putative_       ----------------------------------------------------------------------
AGC67072.1*GH106hypotheti       ----------------------------------------------------------------------
CAA72194.1_GH13_alpha-amy       ----------------------------------------------------------------------
Consensus_aa:                   ......................................................................
Consensus_ss:


Conservation:                                                                       55
3cih_chainA_p005                ----------------------------------------------------------------------
WP_008765775.1_GH106_BT_0  299  WTPEGSEPGSEDMDAAKWKPNLKIKELRLHREARLDQWEGKAGLVWRVASSTKKEEIGEQDCYALSQIIN  368
5mqm_chainA_p001           270  WTPEGSEPGSEDMDAAKWKPNLKIKELRLHREARLDQWEGKAGLVWRVASSTKKEEIGEQDCYALSQIIN  339
BAC68538.1_GH78_SaRha78A_  465  DNDVPEQI------VAQVDGPVRIAKELPA------------RKVTEPK--------------PGVFVLD  502
3w5n_chainA_p002           463  DNDVPEQI------VAQVDGPVRIAKELPA------------RKVTEPK--------------PGVFVLD  500
AAO76120.1_GH78_putative_  328  SIPSGTL-------RAQMMPGMKVTETLKP------------VSIQKM---------------GDKYIMD  363
CCA90848.1_GH106_RHA-P__N  399  -------------------GPHDNVTGVDP-----------------------------------DSVID  414
```

```
BAD12237.1_GH106_alpha-L-  375  ----LAEK------------SAADLPAIDP-----------------------------------AKVVD  393
CDF79921.1_GH78_alpha-L-r  315  PVTSTIK-------LEPKRHTTVKSKIVLE------------SKEIILK--------------ADAAIFD  351
WP_084817526.1_GH106_hypo  204  --------------IIPSEA------VIQK-----------------------------------QEIIN  218
BAB62315.1_GH78_rhamnosid  399  GSNVWRTL------AERRAVPRSVLNAILPVPE------PGVLPVFEDG--------------DCELVID  442
2okx_chainA_p004           397  GSNVWRTL------AERRAVPRSVLNAILPVPE------PGVLPVFEDG--------------DCELVID  440
WP_018627535.1_GH106_dist  174  ---------------------------------------------------------------RASYFIC  180
ACT02314.1_GH106_dist._gl  164  --------------------------------------------------------------SLLNECID  171
AEX05711.1_GH78_KoRha__Kl   54  -----------------RAEPVSTLAAF--------------YQRELSS--------------GDSVIID   78
4xhc_chainA_p003            44  -----------------RAEPVSTLAAF--------------YQRELSS--------------GDSVIID   68
NP_813056.1_GH106_hypothe  154  -------------------------PYSVL------------SRVMAYD--------------ENGRCIN  172
CAB53341.1_GH78_Ram78A_al  278  DLDVNKL-------EPRRSPGIKIKERIKP------------AEIIRTP--------------EGETVID  314
AAV43293.1_GH78_alfa-l-rh  275  NKSTSIV-------RDRLSLPIMKKEVLKV------------KEIIHTP--------------KNEIVLD  311
ACM23671.1*GH106Glycoside       ----------------------------------------------------------------------
ACM61646.1*GH106conserved       ----------------------------------------------------------------------
AGL50002.1*GH106putative_       ----------------------------------------------------------------------
AGC67072.1*GH106hypotheti  161  ---------------------------------------------------------------SSYKRIE  167
CAA72194.1_GH13_alpha-amy       ----------------------------------------------------------------------
Consensus_aa:                   ...................................................................hls
Consensus_ss:                                                                                      eee

Conservation:                   55
3cih_chainA_p005                ----------------------------------------------------------------------
WP_008765775.1_GH106_BT_0  369  LTDPFKNSPSDNFKERTLTATLPK-------GKWKLLRMGHTATGHTN------------------ATAG  413
5mqm_chainA_p001           340  LTDPF-----------TLTATLPK-------GKWKLLRMGHTATGHTN------------------ATAG  373
BAC68538.1_GH78_SaRha78A_  503  LGQNM---------VGSVRLRVSGD-----AGTTVRLRHAEVLNPDGTIYT-------------ANLRSA  545
3w5n_chainA_p002           501  LGQNM---------VGSVRLRVSGD-----AGTTVRLRHAEVLNPDGTIYT-------------ANLRSA  543
AAO76120.1_GH78_putative_  364  TGQNL---------AGWVRFRIKGQ-----AGDSIRLHFAERLESDGEIFT-------------KNLRDA  406
CCA90848.1_GH106_RHA-P__N  415  LTDKLK-------ADGTLDWAAPKLP---AGQHWRVLRLGYSLLGTTN------------------HPAP  456
BAD12237.1_GH106_alpha-L-  394  LTDRLR-------PDGTLDWTAPA------GSDWRIVRMGYSLTGKTN------------------HPAT  432
CDF79921.1_GH78_alpha-L-r  352  LQQNM---------VGVPLLKVPMK-----MGDTLKIRFAEMLSPDGTFYT-------------DNYRSA  394
WP_084817526.1_GH106_hypo  219  LTAHLN-------DAGILEWKVPK-------GEWVILRLGHTPTGKKN------------------HPAP  256
BAB62315.1_GH78_rhamnosid  443  LGAER---------SGFIGFELEAP-----AGTIIDAYGVEYMREGYTQH----------------TYGL  482
2okx_chainA_p004           441  LGAER---------SGFIGFELEAP-----AGTIIDAYGVEYMREGYTQH----------------TYGL  480
WP_018627535.1_GH106_dist  181  LKKEG-----------------------------------------------------------------  185
ACT02314.1_GH106_dist._gl  172  LTDLIT--------DGILYWDIPE-------GYWRVFVISESKDGGSA----------------------  204
AEX05711.1_GH78_KoRha__Kl   79  FGSHY---------VGYLHFLCQSAGSPPDAPAHLQLTFGETLSEVCEPFSDYQGWLSSSWLQQQDLWLD  139
4xhc_chainA_p003            69  FGSHY---------VGYLHFLCQSAGSPPDAPAHLQLTFGETLSEVCEPFSDYQGWLSSSWLQQQDLWLD  129
NP_813056.1_GH106_hypothe  173  LTSHVR--------KDKLVWKAPAG-----KWKVIALYNS--KTRQKV------------------KRAA  209
CAB53341.1_GH78_Ram78A_al  315  MGQNM---------VGWLEFTNRAP-----KGAEIMLQFGEVLQDG-NFYR-------------DNLRTA  356
AAV43293.1_GH78_alfa-l-rh  312  FGQNH---------AGWPVFINRLA-----RGKKITLQMGEILQDG-NFYN-------------KNLRLA  353
ACM23671.1*GH106Glycoside  130  --------------GNEELFVV-----------KRTMKLGDPWFNGT-----------------------  151
ACM61646.1*GH106conserved  131  --------------RNTKYIVA-----------KRTMKLGDKWFNGS-----------------------  152
AGL50002.1*GH106putative_  130  --------------DGEEFYVV-----------KRVMKLGDPWFNGT-----------------------  151
AGC67072.1*GH106hypotheti  168  SGDNDS--------AEGET--------------WYAYRVISGDLPWF-----------------------  192
CAA72194.1_GH13_alpha-amy  164  --------------SPYWD--------------YYIMSLEDHSGQDHWHWK-------------------  186
Consensus_aa:                   hspp..........ss...h.h..............h.hsc.............................
Consensus_ss:                                    eeeee           eeeeeeee

Conservation:                                            5
3cih_chainA_p005           284  -----------------------------------------------------------RGNFR------  288
WP_008765775.1_GH106_BT_0  414  ----------------------GGKG---LECDKFN-----PKAV-------------------------  428
5mqm_chainA_p001           374  ----------------------GGKG---LECDKFN-----PKAV-------------------------  388
BAC68538.1_GH78_SaRha78A_  546  AATDTYTLKGQG--EETYEPRFTFHGFRYVEVTGFPG----KPSTTSVTGRVMHTSAPFTFEFE------  603
3w5n_chainA_p002           544  AATDTYTLKGQG--EETYEPRFTFHGFRYVEVTGFPG----KPSTTSVTGRVMHTSAPFTFEFE------  601
AAO76120.1_GH78_putative_  407  HCTDIYVVSGREPQGATWAPRFVYHGFRYVEISGYP-----DAKTEDFVVEVVEDEMDHTGSFS------  465
CCA90848.1_GH106_RHA-P__N  457  P---------------------EATG---LEVDKFD-----GEAV-------------------------  472
BAD12237.1_GH106_alpha-L-  433  P---------------------EATG---LEVDKYD-----PAAV-------------------------  448
CDF79921.1_GH78_alpha-L-r  395  QSTDYYIAAKEG--TIEWMPKFTFHGFRYVELSGFDAS--KTPSKNWVKGVVQYSNFNENGSFT------  454
WP_084817526.1_GH106_hypo  257  K---------------------GGHG---LEVDKMS----------------------------------  268
BAB62315.1_GH78_rhamnosid  483  DNTFRYICREGR---QSYVS-PVRRGFRYLFLTVRGNS--APVKLHEIYIRQSTYPVAEQGSFR------  540
2okx_chainA_p004           481  DNTFRYICREGR---QSYVS-PVRRGFRYLFLTVRGNS--APVKLHEIYIRQSTYPVAEQGSFR------  538
WP_018627535.1_GH106_dist  186  -DHFTEVTAG-----------ALGKGDYYLFR--------------------KLSYTNQAGMVGPPNYPL  223
ACT02314.1_GH106_dist._gl  205  -----------------------AQE---DYLNPLV-----RDSV-------------------------  218
AEX05711.1_GH78_KoRha__Kl  140  VLPAEIDLPR-------------RYCFRYLKVEVKAVSRKFRLQFTQIEVNAVTSASGACPAAT------  190
4xhc_chainA_p003           130  VLPAEIDLPR-------------RYCFRYLKVEVKAVSRKFRLQFTQIEVNAVTSASGACPAAT------  180
NP_813056.1_GH106_hypothe  210  P---------------------GGEG---YVMNHLS----------------------------------  221
CAB53341.1_GH78_Ram78A_al  357  KCEFHYISDGKV---KKVRPHFTFYGFRYVKLTKWEG----EVNPEDFTGLVLYSDLERTGNIT------  413
AAV43293.1_GH78_alfa-l-rh  354  RAAFTYISDGEE---KLIRPHFTYFGFRYVKISGVT-----DVNKDDFESWVLYSDLKQTGFIK------  409
ACM23671.1*GH106Glycoside  152  -----------------------------CYVDLLS----------------------------------  158
ACM61646.1*GH106conserved  153  -----------------------------CYVDLLS----------------------------------  159
AGL50002.1*GH106putative_  152  -----------------------------CYVDLLS----------------------------------  158
AGC67072.1*GH106hypotheti  193  ------------------------NNQ--AYVDTL-----------------------------------  201
CAA72194.1_GH13_alpha-amy  187  ------INSKG-------------QKV--WYFGLFGYNMPDLNHD-------------------------  210
Consensus_aa:                   ........................pt...hbhs.hs..................................
Consensus_ss:                                             e  eeee

Conservation:                                                           9 5                       85
3cih_chainA_p005           289  ---CNDEELNCIWEVGAY----TMHLTT--------REFFIDGIKR-----------------DRWVWSG  326
WP_008765775.1_GH106_BT_0  429  -----RKQFDNWFAQAFV----KTNPDV---ARRVLKYMHVDSWEC-----------------GSQNWSD  469
5mqm_chainA_p001           389  -----RKQFDNWFAQAFV----KTNPDV---ARRVLKYMHVDSWEC-----------------GSQNWSD  429
BAC68538.1_GH78_SaRha78A_  604  ---TNVPMLNKLHSNITW----GQRGNF--------LSVPTDTPAR----------------DERLGWTG  642
3w5n_chainA_p002           602  ---TNVPMLNKLHSNITW----GQRGNF--------LSVPTDTPAR----------------DERLGWTG  640
AAO76120.1_GH78_putative_  466  ---CSDKTLNQVVRNAFW----GIRSNY--------KGMPVDCPQR----------------NERQPWLG  504
CCA90848.1_GH106_RHA-P__N  473  -----REYLEHYIGMYKD----AAGPDM--VGKRGVRALLTDSIEV-----------------GEANWTP  514
BAD12237.1_GH106_alpha-L-  449  -----RRYLETYLGLYRD----AVGAEW--IGKKGIRALLTDSIEV-----------------GASNWTP  490
CDF79921.1_GH78_alpha-L-r  455  ---SSHEKLNQLQSNIVW----GLRGNF--------FDIPTDCPQR----------------DERMGWTG  493
WP_084817526.1_GH106_hypo  269  -----TKAVDVYWEGGIQ----PILNKLGDLVGTTVNNCLIDSYEV-----------------GTANWTA  312
```

```
BAB62315.1_GH78_rhamnosid  541  ---CSDALLNATWEISRH----TTRL--------CMEDTFVDCPSY-----------------EQVFWVG  578
2okx_chainA_p004           539  ---CSDALLNATWEISRH----TTRL--------CMEDTFVDCPSY-----------------EQVFWVG  576
WP_018627535.1_GH106_dist  224  VDLLVKGVTEKFIDITFSGYKKAFGDAF----GKTVPGTFSDEPSIPTHG------------SLTTRWTP  277
ACT02314.1_GH106_dist._gl  219  -----RILLDTVYEAVYK----RYKADF----GSTFAGFFSDEPGFYNDKTTFDFQSRPGKKGVPLPWSS  275
AEX05711.1_GH78_KoRha__Kl  191  ---TSDPQLRAIDNVAVL----TLQNCM--------QEVFEDGPKR----------------DRRL-WLG  228
4xhc_chainA_p003           181  ---TSDPQLRAIDNVAVL----TLQNCM--------QEVFEDGPKR----------------DRRL-WLG  218
NP_813056.1_GH106_hypothe  222  -----KTAVKNYLSRFNR----AFKSSK----TSYPHTFFNDSYEV-----------------YQADWTE  261
CAB53341.1_GH78_Ram78A_al  414  ---TDNSLVNRLFLNALW----SQKGNF--------LDVPTDCPQR----------------DERMGWTG  452
AAV43293.1_GH78_alfa-l-rh  410  ---TNNDKVNRLFKNVIW----GQKSNF--------MDVPTDCPQR----------------DERLGWTG  448
ACM23671.1*GH106Glycoside  159  -----KKVTEAFIRSTHEKYRKVCGEFF----GSSIPGIFTDEPTYLRVHY---------PEIPTLPWTD  210
ACM61646.1*GH106conserved  160  -----KEATLEFINLTHERYKSYCQDYF----GDAMPGIFTDEPTYLRVHY---------KDIATLPWTE  211
AGL50002.1*GH106putative_  159  -----RETTEAFLRSTHERYKKSCGDLF----RVSIPGIFTDEPTYLRVHH---------PKETTLPWTE  210
AGC67072.1*GH106hypotheti  202  ----NKKAVEKFIEVTHEKYFEILGGEF----SKSVPAVFTDEPQFVLKQNLPE---PDSRQEVGIPYTD  260
CAA72194.1_GH13_alpha-amy  211  ----SQKVREEVKKIVDF----WISK--------GVDGFRIDAAK-------------------------  239
Consensus_aa:                   .....p..hp.hhp.h.......h...h.........sh..D..p..................phsWhs
Consensus_ss:                        hhhhhhhhhhhhh    hhhh

Conservation:                                                              5
3cih_chainA_p005           327  DAIQSY------------LMNYYLFF-------------DSESVKRTIWLLRGKDPV-------------  358
WP_008765775.1_GH106_BT_0  470  TFAAEFRKRRGYDLMPYLPLLAGIPME------------SAERSEKILRDVRTTIG--------------  513
5mqm_chainA_p001           430  TFAAEFRKRRGYDLMPYLPLLAGIPME------------SAERSEKILRDVRTTIG--------------  473
BAC68538.1_GH78_SaRha78A_  643  DINVFA------------PTAAYTME-------------SARFLTKWLVDLRDAQTSDGAFTD---VAPA  684
3w5n_chainA_p002           641  DINVFA------------PTAAYTME-------------SARFLTKWLVDLRDAQTSDGAFTD---VAPA  682
AAO76120.1_GH78_putative_  505  DHAMGS------------WGESMFFD-------------NHAMYNKWARDIREAQREDGCIPD---VAPA  546
CCA90848.1_GH106_RHA-P__N  515  RMLEQFQRLRGYDARPWLPALTGTLVG------------TREQSDRFLYDYRRTLA--------------  558
BAD12237.1_GH106_alpha-L-  491  RMVEEFRARRGYDPVPFLPALTGAVVG------------SAARSDAFLHDFRQTLA--------------  534
CDF79921.1_GH78_alpha-L-r  494  DAQVFG------------PTSMFNAD-------------VYKFWASWMQSVRESQYDNGGIPF---VVPD  535
WP_084817526.1_GH106_hypo  313  GFDAEFETLRGYSLVSYLPTLAGYYVE------------SGEITERFLWDFRRTIG--------------  356
BAB62315.1_GH78_rhamnosid  579  DS------------RNEALVNYYVFG-------------ETEIVERCLNLVPGSADETPLYLD---QVPS  620
2okx_chainA_p004           577  DS------------RNEALVNYYVFG-------------ETEIVERCLNLVPGSADETPLYLD---QVPS  618
WP_018627535.1_GH106_dist  278  DLFAVFKEKWGYDLVPHLPSLFEETGDWRKIRHNYQETLLQLFIDRWSKPMQAFTQKNHLK-----WTGH  342
ACT02314.1_GH106_dist._gl  276  AMPGLLEQALGGDYRKKLYLLWQED--------------ESAQFNAVRYAYMNIVS--------------  317
AEX05711.1_GH78_KoRha__Kl  229  DLRLQA------------LVNDVTFA-------------RHDLVRRCLYLFAGHTREDGMVSANVFVQPD  273
4xhc_chainA_p003           219  DLRLQA------------LVNDVTFA-------------RHDLVRRCLYLFAGHTREDGMVSANVFVQPD  263
NP_813056.1_GH106_hypothe  262  DFLDQFARRRGYKLEEHFPEFLDEN--------------RPEISRRIVSDYRETIS--------------  303
CAB53341.1_GH78_Ram78A_al  453  DAQVFS------------GAAAFNMD-------------VFAFFGKYLYDLKQEQKARGGNVP--VVVPA  495
AAV43293.1_GH78_alfa-l-rh  449  DAEIFA------------PTASFNMN-------------TYEFYKKYAKDMLVEQEDNKGMLP--IIVPS  491
ACM23671.1*GH106Glycoside  211  RFPEEFLKRKGYDIREHFEELFFN----------------TKDYMKVRYDFFDVAT--------------  250
ACM61646.1*GH106conserved  212  KLPERFLQKKGYDIKEHFEELFFN----------------VGNYHKVRFDFFDIAL--------------  251
AGL50002.1*GH106putative_  211  RFPEEFLRRKGYDIRDHLEELFFN----------------VKDYMKVRYDFFDVAT--------------  250
AGC67072.1*GH106hypotheti  261  LFEESFRQRFGCSFLDSLPEIFWEL--------------EGGKFSRIRYWYHEHIA--------------  302
CAA72194.1_GH13_alpha-amy  240  ----------------HIYGWSWDDG--------------------------------------------  249
Consensus_aa:                   ph...h...............................p....p+h...h...................
Consensus_ss:                   hhhhhh         hhhhhhhh                  hhhhhhhhhhhhhhhh

Conservation:
3cih_chainA_p005           359  -TSHSNTIMDYTFYWFLSVYDYYMYSGDRHFVNQLYPRMQTMMDYVL--GRTNKNGMVEG----MSGDW-  420
WP_008765775.1_GH106_BT_0  514  ---------------------------E-----LVVDVFYQVLADCA--KEYDCQFSAEC---VAPTM--  544
5mqm_chainA_p001           474  ---------------------------E-----LVVDVFYQVLADCA--KEYDCQFSAEC---VAPTM--  504
BAC68538.1_GH78_SaRha78A_  685  VGNLGNGVAGWGDAGVTVPWALYQAYGDR----QVLADALPSVHAWL--RYLEKHSDG-----LLRPADG  743
3w5n_chainA_p002           683  VGNLGNGVAGWGDAGVTVPWALYQAYGDR----QVLADALPSVHAWL--RYLEKHSDG-----LLRPADG  741
AAO76120.1_GH78_putative_  547  YWNYYSDNVTWPATLPLVCDMLFTNYGDI----RPIEDNYPAIKKWI--SHIREYYMTKD---YIITKDK  607
CCA90848.1_GH106_RHA-P__N  559  ---------------------------D-----LLASEHYGTVADVA--HENDLKVYGEA---LEDHRPM  591
BAD12237.1_GH106_alpha-L-  535  ---------------------------D-----LLADAHYGTIAKVA--HEQGLIVYGEA---LENGRPV  567
CDF79921.1_GH78_alpha-L-r  536  VLHNGKVSSGWGDVCTIIPWKIYYRTGDV----GILEENYDMMKKWV--AHHQATSKD-----FISHMNS  594
WP_084817526.1_GH106_hypo  357  ---------------------------D-----LMAKNYYAHFRDLC--HKNGLKFSVEP---YWGP---  386
BAB62315.1_GH78_rhamnosid  621  AWSSV--IPNWTFFWILACREYAAHTGNE----AFAARIWPAVKHTL--THYLEHIDDSG--LLNMAGWN  680
2okx_chainA_p004           619  AWSSV--IPNWTFFWILACREYAAHTGNE----AFAARIWPAVKHTL--THYLEHIDDSG--LLNMAGWN  678
WP_018627535.1_GH106_dist  343  YWEHGWPDPGEGPDNM--AMYAWHDQPGI----DMLFNQFNES---------------------SPNAQ  384
ACT02314.1_GH106_dist._gl  318  ---------------------------K-----LYADNFCSQIGDWC--RARGVEYIGHV---LEDNNVH  350
AEX05711.1_GH78_KoRha__Kl  274  VIADDTFLFDYSLFFVDVLYNYLQSAEDM----ATARELWPTARRQV--ELALTRCDASGVVRDSDDWWV  337
4xhc_chainA_p003           264  VIADDTFLFDYSLFFVDVLYNYLQSAEDM----ATARELWPTARRQV--ELALTRCDASGVVRDSDDWWV  327
NP_813056.1_GH106_hypothe  304  ---------------------------D-----LLLENFTRQWTDWA--HKNGSITRNQA---HGSP---  333
CAB53341.1_GH78_Ram78A_al  496  HDVKQNGACGWGDAAVIIPWNMYLHYGDV----SILEQQYKSMKGWV--DYIKSKDDAAGGRRLWLNDFH  559
AAV43293.1_GH78_alfa-l-rh  492  LKQKSTGMAIWSDAATIIPWVTYRFFDDL----GVLKQNYSQMKNWV--DWITQNTKTKY---LWIGQMQ  552
ACM23671.1*GH106Glycoside  251  ---------------------------E-----LFIENFTIPYAKWC--EENGISMTGHY---MAEDTLR  283
ACM61646.1*GH106conserved  252  ---------------------------E-----MFIENFTIPYAKWC--EENGIMMTGHY---MAEDTMR  284
AGL50002.1*GH106putative_  251  ---------------------------S-----LFIENFTIPYAKWC--EENGIFMTGHY---MAEDTLR  283
AGC67072.1*GH106hypotheti  303  ---------------------------E-----LFASSFADTVGRWC--REHNLMLTGHM---MAESTLE  335
CAA72194.1_GH13_alpha-amy  250  ---------------------------I-----QESAEYFEWFRDYVLSKKPDAILVGEV---FSGN---  281
Consensus_aa:                   ...........................p......hh.p.h..h.phh..p.......sp....h..s...
Consensus_ss:                                 hhh  hhhhh          hhhhhhhhhhhhhh  hhh

Conservation:
3cih_chainA_p005           421  -------------------------------VFVDWADGYLD----------KKGE---LSFEQVLFCRS  446
WP_008765775.1_GH106_BT_0  545  -----------------VSDGLLHYQKVDLPMGEFWLNSPTH-----------------DKPN------D  574
5mqm_chainA_p001           505  -----------------VSDGLLHYQKVDLPMGEFWLNSPTH-----------------DKPN------D  534
BAC68538.1_GH78_SaRha78A_  744  -----------------YGDWLNVSD--------------------------ETPK---DVIATAYFAHS  767
3w5n_chainA_p002           742  -----------------YGDWLNVSD--------------------------ETPK---DVIATAYFAHS  765
AAO76120.1_GH78_putative_  608  -----------------YGDWCVPPESLEMIHS----QDPAR----------KTDG---ALIATAYYLKV  643
CCA90848.1_GH106_RHA-P__N  592  -----------------LGDDMAMRSHADIPMAALWTFNRDE----------GPRQ---TLIA------D  625
BAD12237.1_GH106_alpha-L-  568  -----------------LGDDLAMRAHADVPMAALWTYNRGS----------APRP---TLIG------D  601
CDF79921.1_GH78_alpha-L-r  595  -----------------FADWLQPYPEN---------GNNKG----------DTSH---SLIGTAFFAHS  625
WP_084817526.1_GH106_hypo  387  ------------------FDNMQVGATGDIVMCEFWSGGY-------------------PFFD------S  413
BAB62315.1_GH78_rhamnosid  681  -----------------LLDWAPIDQP--------------------------NEGI---VTHQNLFLVKA  705
2okx_chainA_p004           679  -----------------LLDWAPIDQP--------------------------NEGI---VTHQNLFLVKA  703
WP_018627535.1_GH106_dist  385  -----------------FGN------------------------------------------------IRS  390
```

```
ACT02314.1_GH106_dist._gl   351  ARLGPGPGHFFRSLWGQGMSGLDVVLWQIVPGFDELSFRNTS----------GEADSEFFHYG------L  404
AEX05711.1_GH78_KoRha__Kl   338  -----------------FIDWQA----------------------------SLNK---QAAAQGVLIYC  358
4xhc_chainA_p003            328  -----------------FIDWQA----------------------------SLNK---QAAAQGVLIYC  348
NP_813056.1_GH106_hypothe   334  ------------------ANLIDVYAAVDIPECEGFGLSQFHIKGLRQDSLTKKND---SDLS------M  376
CAB53341.1_GH78_Ram78A_al   560  -----------------YGDCVSLDVEDP--------FNRFG----------GTEH---AYLASAFYSYS  591
AAV43293.1_GH78_alfa-l-rh   553  -----------------LGDWLSLDNGA----------NPQG----------KTNE---DYIASIYYFVS  582
ACM23671.1*GH106Glycoside   284  GQVE------------WIGAAMPHYEYMQIPGVDKLARHL-------------------EQVI------T  316
ACM61646.1*GH106conserved   285  GQIE------------WIGAAMPHYEYMQLPGVDKLARHL-------------------EQVV------T  317
AGL50002.1*GH106putative_   284  GQVE------------WIGAAMPHYEYMQIPGIDKLARHL-------------------EQVV------T  316
AGC67072.1*GH106hypotheti   336  SQTR------------ALGEAMRSYRSFDLPGVDILANKY--------------------EYS------T  367
CAA72194.1_GH13_alpha-amy   282  ------------------TYDLSLYPIPV-----------------------------------------  292
Consensus_aa:                    .................h.s.h.h..................................hs......s
Consensus_ss:                                                                                      h

Conservation:                           9   5                          6
3cih_chainA_p005            447  LETMALCADLVGD----------KDGQQKYEKLASALKAKLEPTFWNN-----QKQAFVHNC--------  493
WP_008765775.1_GH106_BT_0   575  MLDAISGAHIYGKN--IIQAEGFTEVRGTWNEHPGILKALLDRNYALG-----INRLFFHVY--VHNPWL  635
5mqm_chainA_p001            535  MLDAISGAHIYGKN--IIQAEGFTEVRGTWNEHPGILKALLDRNYALG-----INRLFFHVY--VHNPWL  595
BAC68538.1_GH78_SaRha78A_   768  ADLAARMATELGK------------DAAPYTDLFTRIRKAFQTAYVAS-----DGKVKGD----------  810
3w5n_chainA_p002            766  ADLAARMATELGK------------DAAPYTDLFTRIRKAFQTAYVAS-----DGKVKGD----------  808
AAO76120.1_GH78_putative_   644  LQLMHRFASLQGL----------TADAKEWEDLEHKMKDAFNAHFLHI-----KEGTSLVPG-HTLYPDS  697
CCA90848.1_GH106_RHA-P__N   626  MKGAASVAHLYGQN--LVAAESMTASMAPWAFAPKDLKRFIDLEFVTG-----VNRPVIHTS--VHVPVD  686
BAD12237.1_GH106_alpha-L-   602  MKGAASVAHIYGQN--IVSAESMTAAFSPWAFAPADLKRVIDLEFVSG-----VNRPIVHTS--VHQPVD  662
CDF79921.1_GH78_alpha-L-r   626  AKLTAKTAEVLGK----------KEEQATYEALYKSVAKAFENAFFKN-----GKVKDVTA---------  671
WP_084817526.1_GH106_hypo   414  PKFVSSIAHLNGSS--IVGAESFTG-IGGWDEHPAELKSIGDRAWAEG-----ITRFIFHTY--VHQPWD  473
BAB62315.1_GH78_rhamnosid   706  LRDSRALAAAAGAT----------EEADAFAARADLLAETINAVLWDE-----EKRAYIDCI--HADGR-  757
2okx_chainA_p004            704  LRDSRALAAAAGAT----------EEADAFAARADLLAETINAVLWDE-----EKRAYIDCI--HADGR-  755
WP_018627535.1_GH106_dist   391  VKELSSVANQLGKE--RTLCETYG--GAGWDLTFKDMKRLGDWQAALG-----VNFMNQHLA--WMSMA-  448
ACT02314.1_GH106_dist._gl   405  AKLGVSLAHIDPKKQGRTMCEVYG--AYGWTEGLKLMKWLTDHMLVRG-----VNYFVPHAFTQKAFPEP  467
AEX05711.1_GH78_KoRha__Kl   359  LQRAIWLAERFEP-----------ELAVSYRQRLQQLKSAALDALWDP-----QQGFYVSGA--------  404
4xhc_chainA_p003            349  LQRAIWLAERFEP-----------ELAVSYRQRLQQLKSAALDALWDP-----QQGFYVSGA--------  394
NP_813056.1_GH106_hypothe   377  LKYASSAAHIAGKP--YTSTETFTWLTEHFRTSLSQCKPDMDLMFVSG-----INHMFFHGT--PYSPKE  437
CAB53341.1_GH78_Ram78A_al   592  AGIVSKAAKILNK----------KEDAEYYRKLSEEVKNAIRKEYFTP-----TGRLAVNT---------  637
AAV43293.1_GH78_alfa-l-rh   583  ASIVSKAARLLHY----------DMESDYYENLARNIKTNILNEFVTE-----KGRIAIDT---------  628
ACM23671.1*GH106Glycoside   317  IKQVSSVAEQLRKK--WVLCETFG--TTGQHVSFLHRKWIADWQAVLG-----ITYINPHLS--LYSMR-  374
ACM61646.1*GH106conserved   318  MKQVSSVAEQLGKK--GVLCETFG--TTGQHVSFLHRKWISDWQAVLG-----ITYINPHLS--LYSMR-  375
AGL50002.1*GH106putative_   317  IKQVSSAAEQLGKK--WVLCETFG--TTGQHVSFLHRKWIADWQAVLG-----VTYINPHLS--LYSMR-  374
AGC67072.1*GH106hypotheti   368  VKQAQSVARQFGKA--GVLSELYG--VTNWDFDFRGHKLQGDWQAAMG-----VSVRVPHLS--WLGMG-  425
CAA72194.1_GH13_alpha-amy   293  ----------------------------FNFALMYSIRNYPEGQDGMIENNWVEESFLFLE--NHDLH-  330
Consensus_aa:                    hp.h..hApbhs.................@p.....h+..hp..hh........p....p.........
Consensus_ss:                    hhh hhhhhh                hhhhh hhhhhhhhhhhhhhhh            eee

Conservation:                                                                          7
3cih_chainA_p005            494  --------------------------VDG--RQSDAVTR----YANMFSVFFDYLNADKQQAIKQSVLL  530
WP_008765775.1_GH106_BT_0   636  DRKP--GMTLDGIGLFFQRDQ-TWWNKGAK-AFCEYITR------CQSLL----QYGH-PVADIAV---F  687
5mqm_chainA_p001            596  DRKP--GMTLDGIGLFFQRDQ-TWWNKGAK-AFCEYITR------CQSLL----QYGH-PVADIAV---F  647
BAC68538.1_GH78_SaRha78A_   811  ------TQSAYVLTLSMNLVPDALRKAAAD-RLVALIEA------KDWHL----STG-------------  850
3w5n_chainA_p002            809  ------TQSAYVLTLSMNLVPDALRKAAAD-RLVALIEA------KDWHL----STG-------------  848
AAO76120.1_GH78_putative_   698  VFYGNNTVTANILPLAFGLVPKAHIKEVAKNAVTTIITT------NKGHI----STG-------------  744
CCA90848.1_GH106_RHA-P__N   687  DKKP--GLSLAIFGQYFNRQE--SWAEMAR-PWVDYIAR------SSLLL----QTGR-NVADVAY---F  737
BAD12237.1_GH106_alpha-L-   663  DKLP--GLSLAIFGQYFNRHD--SWAEMAR-PWVDYMAR------TGFLL----QQGR-DHADIAW---F  713
CDF79921.1_GH78_alpha-L-r   672  ------TQTSYLLALAFDLLSEENKENAKQ-QLLEKISE------ADNHL----RTG-------------  711
WP_084817526.1_GH106_hypo   474  -VAP--GLALSYHGTDFNRLN--TWWRQGK-AFMDYIAR------SQFML----QQGK-NVADVLV---F  523
BAB62315.1_GH78_rhamnosid   758  --------RSDVYSMQTQ---------VVA-YLCGVAQGEREAVIEGYLS----SPPP-AFVQIGS---P  801
2okx_chainA_p004            756  --------RSDVYSMQTQ---------VVA-YLCGVAQGEREAVIEGYLS----SPPP-AFVQIGS---P  799
WP_018627535.1_GH106_dist   449  ------GARKYDYPTTFSYQN--SWWPYYR-SLNEYFAR------LSYAL----SRGI-QKNDILI---L  495
ACT02314.1_GH106_dist._gl   468  DCPP---------HMYADGKN--PQYRYYK-HLNHYINR------MSHLI----SGGR-HVATAAV---M  511
AEX05711.1_GH78_KoRha__Kl   405  RRQV--SWASQIWLVLAEVGTPQQRREIMR-NLEKNP---------PAVAM----NTP-------------  446
4xhc_chainA_p003            395  RRQV--SWASQIWLVLAEVGTPQQRREIMR-NLEKNP---------PAVAM----NTP-------------  436
NP_813056.1_GH106_hypothe   438  AEWP--GWLFYA-SINMNPTN--SIWHDAP-SFFDYITR------CQSFL----QMGK-PDNDFLI---Y  487
CAB53341.1_GH78_Ram78A_al   638  -------QTAYVIALYMDLVPDEWKERVAF-ELRKKLKE------TKYHL----RTG-------------  676
AAV43293.1_GH78_alfa-l-rh   629  -------QTALVLALHFGLVHDYQKSQVVA-DLVKKVKD------DNKHL----QTG-------------  667
ACM23671.1*GH106Glycoside   375  ------GERKRDYPPNLFYQQ--PWWKNEK-RFSDYLAR------LNYLM----TQGK-REVKVLM---I  421
ACM61646.1*GH106conserved   376  ------GERKRDYPPNLFYQQ--PWWEDEK-FFADYLAR------ISYIA----GLGK-RDVDVLV---L  422
AGL50002.1*GH106putative_   375  ------GERKRDYPPNLFYQQ--PWWKNER-FLSDYFAR------LNHIV----TQGK-REVKVLM---I  421
AGC67072.1*GH106hypotheti   426  ------GESKRDYPAPVDYHS--PWYKKYH-IIEDHFSR------VNVCM----TRGV-PCVNIAV---I  472
CAA72194.1_GH13_alpha-amy   331  -------RFFSHLQEHYKKFS--ESDYEFI-KKRAALWY------FLIFT----LKGS-----PVI---Y  372
Consensus_aa:                    ......b....hs..hp..p.....p.....h.p.h..........hh....p.s.............
Consensus_ss:                                              hhh hhhhhhhh      hhhhh             eeee    e

Conservation:
3cih_chainA_p005            531  NDEILKITTPYMR--------------FYELEALCALGEQETVMKEMKAYWGGMLKAGATSFWEKYNPEE  586
WP_008765775.1_GH106_BT_0   688  TGEEMPRRS------------------ILPER-LVPSLPGIFGAERVESERIRLANEG------------  726
5mqm_chainA_p001            648  TGEEMPRRS------------------ILPER-LVPSLPGIFGAERVESERIRLANEG------------  686
BAC68538.1_GH78_SaRha78A_        ----------------------------------------------------------------------
3w5n_chainA_p002                 ----------------------------------------------------------------------
AAO76120.1_GH78_putative_        ----------------------------------------------------------------------
CCA90848.1_GH106_RHA-P__N   738  YGEEAPLTG------------------LYGDE-PVA----------------------------------  754
BAD12237.1_GH106_alpha-L-   714  HGEDMPVTA------------------LFEHG-EPA----------------------------------  730
CDF79921.1_GH78_alpha-L-r        ----------------------------------------------------------------------
WP_084817526.1_GH106_hypo   524  TGESSPNTA------------------FLLPE--------------------------------------  537
BAB62315.1_GH78_rhamnosid   802  FM--------------------------------------------------------------------  803
2okx_chainA_p004            800  FM--------------------------------------------------------------------  801
WP_018627535.1_GH106_dist   496  EPTSTAWMYHSRVKSNP---RFFETGTLFQSF-ITR----------------------------------  527
ACT02314.1_GH106_dist._gl   512  YHAEAEWSG---------------KAMYFHKP-AKV----------------------------------  531
AEX05711.1_GH78_KoRha__Kl        ----------------------------------------------------------------------
4xhc_chainA_p003                 ----------------------------------------------------------------------
```

```
NP_813056.1_GH106_hypothe   488  LPVYDMWDEQPGRLLLFSIHHMAKLAPKFIDA-IHR-----------------------------------   522
CAB53341.1_GH78_Ram78A_al        ----------------------------------------------------------------------
AAV43293.1_GH78_alfa-l-rh        ----------------------------------------------------------------------
ACM23671.1*GH106Glycoside   422  HPITSAWCVYSKFDDE-----IDKLNESFDRT-VKE-----------------------------------   451
ACM61646.1*GH106conserved   423  HPISSAWAEYSKFDDS-----VDKLDILLDKT-VKE-----------------------------------   452
AGL50002.1*GH106putative_   422  HPISSAWCVYSKFDDE-----IDKLNELFDTI-TKE-----------------------------------   451
AGC67072.1*GH106hypotheti   473  HPIESYWLLWGPDSQTK--QKRLKLQQEFESI-IDW-----------------------------------   505
CAA72194.1_GH13_alpha-amy   373  YGGEIGTRGF----------------KWHGP----------------------------------------   387
Consensus_aa:                    ......................................................................
Consensus_ss:


Conservation:
3cih_chainA_p005            587  SGTQHLAMYGRPYGKSLCHAWGASPIYLLGKYYLGVKPTKEGYKEF------------------------   632
WP_008765775.1_GH106_BT_0   727  ---QPLRVRPVGVTHSANMSDPEKWVNPLRGYAYDSFNKDALLRLA------------KAENG-RMTLPG   780
5mqm_chainA_p001            687  ---QPLRVRPVGVTHSANMSDPEKWVNPLRGYAYDSFNKDALLRLA------------KAENG-RMTLPG   740
BAC68538.1_GH78_SaRha78A_   851  ------------------------------------FLGTPRLLPV------------LTDTG-HT----   867
3w5n_chainA_p002            849  ------------------------------------FLGTPRLLPV------------LTDTG-HT----   865
AAO76120.1_GH78_putative_   745  ------------------------------------VIGTQWLMRE------------LSRRG-HT----   761
CCA90848.1_GH106_RHA-P__N   755  ---------------------------DAPVRYAYDYINFNALTEL------------LANDGEDLVAPS   785
BAD12237.1_GH106_alpha-L-   731  ---------------------------GLPRRHGYDFVNADILAKV------------LKVEGGELVAPG   761
CDF79921.1_GH78_alpha-L-r   712  ------------------------------------FLGTPLLSEV------------LDETG-EI----   728
WP_084817526.1_GH106_hypo   538  ---------------------------IKQLGYDYDLIGSNKLSDL------------FVKNG-KICTPV   567
BAB62315.1_GH78_rhamnosid   804  ---------------------------------------SFFYYEA------------LEKAG-RQ----   817
2okx_chainA_p004            802  ---------------------------------------SFFYYEA------------LEKAG-RQ----   815
WP_018627535.1_GH106_dist   528  ---------------------------LEKAQAEYDLGSENIIKDHG------------SVYRD-QFRI-G   557
ACT02314.1_GH106_dist._gl   532  ---------------------------LMQHQIDCDILPIDVLLESA------------SIKDN-RLVV-H   561
AEX05711.1_GH78_KoRha__Kl   447  ------------------------------------YLR-HHYIAA------------LLQCG-LR----   462
4xhc_chainA_p003            437  ------------------------------------YLR-HHYIAA------------LLQCG-LR----   452
NP_813056.1_GH106_hypothe   523  ---------------------------INNSGYDGDYISDNFIRST------------RFKDG-QLITSG   552
CAB53341.1_GH78_Ram78A_al   677  ------------------------------------FLGTPYLCRV------------LSEYG-SN----   693
AAV43293.1_GH78_alfa-l-rh   668  ------------------------------------FVGTPFLLSV------------LSNNN-QH----   684
ACM23671.1*GH106Glycoside   452  ---------------------------LVSNKIDFHFGDETILSKHG------------RVSGE-KLVV-G   481
ACM61646.1*GH106conserved   453  ---------------------------LIANKIDFHFGDEIILSKYA------------QVENC-KIKV-G   482
AGL50002.1*GH106putative_   452  ---------------------------LVANKIDFHFGDEMILSKHG------------RVKEA-KLRV-G   481
AGC67072.1*GH106hypotheti   506  ---------------------------LLFSNLDFDFIAESMVKELY------------RGSGNR-HTQF-G   536
CAA72194.1_GH13_alpha-amy   388  --------------------------------VYDEPVREPMQWYASGTGEGQTFWTKEVYKNA-GITF-G   424
Consensus_aa:                    ....................................h.sp.hh.ph..............p.s.ph....
Consensus_ss:                                                         hhhhhh               hh


Conservation:                                                                 5                   6
3cih_chainA_p005            633  ---AVSPVLGGLKWMEGTVPTPNGDIHVYMDNKTIKVKATEGKGYLTIQSRRQPKANMGTVEKVSEG---   696
WP_008765775.1_GH106_BT_0   781  GASYKVLVLPLP---------RPMNPDPA------ALSPEVKQKIN------------ELKEAGILIPSL   823
5mqm_chainA_p001            741  GASYKVLVLPLP---------RPMNPDPA------ALSPEVKQKIN------------ELKEAGILIPSL   783
BAC68538.1_GH78_SaRha78A_   868  ----------------------------------------DVAYRLLHQRTFPSWG---YPIDKGSTTMWE   895
3w5n_chainA_p002            866  ----------------------------------------DVAYRLLHQRTFPSWG---YPIDKGSTTMWE   893
AAO76120.1_GH78_putative_   762  ----------------------------------------DVAYLLATNDTYPSWG---YMAAQGATTIWE   789
CCA90848.1_GH106_RHA-P__N   786  GARYKTIYLGGS----------SSHMT-----------LAALRKLA------------ALVVGGATVVGK   822
BAD12237.1_GH106_alpha-L-   762  GARYRALYLGGS----------SRVMT-----------LPTLRRIA------------QLAEQGATVIGT   798
CDF79921.1_GH78_alpha-L-r   729  ----------------------------------------DLMYKLLFNETYPSWF---YSINQGATTIWE   756
WP_084817526.1_GH106_hypo   568  GGQYDVLMLPES----------DWIK------------PETLHKIE------------DLVKDGAKVIGS   603
BAB62315.1_GH78_rhamnosid   818  ----------------------------------------TLMLDDIRRNYG-------QMLRYDATTCWE   841
2okx_chainA_p004            816  ----------------------------------------TLMLDDIRRNYG-------QMLRYDATTCWE   839
WP_018627535.1_GH106_dist   558  NASYKTVVIPPG---------MENVN-------------KETYLLLR-----------QFVANGGKLVLF   594
ACT02314.1_GH106_dist._gl   562  QEDYKCLIVPYA----------EALP------------AAIIRSLV------------QYAEQGLEIFFV   597
AEX05711.1_GH78_KoRha__Kl   463  ----------------------------------------DEAIAQIKAYWG-------AMVDYGADTFWE   486
4xhc_chainA_p003            453  ----------------------------------------DEAIAQIKAYWG-------AMVDYGADTFWE   476
NP_813056.1_GH106_hypothe   553  GTGYKALVVPAA----------HLMP------------NDVLAHLL------------KLAQQGATIVFL   588
CAB53341.1_GH78_Ram78A_al   694  ----------------------------------------DIAYRLLTNDTYPGWL---YPVTMGATTIWE   721
AAV43293.1_GH78_alfa-l-rh   685  ----------------------------------------HLAMDIFMQEDCPSWL---YEVNMGATTIWE   712
ACM23671.1*GH106Glycoside   482  NCEYDIVLVPPL----------LNLR------------RSTVDLLN------------EFSKNGGKILVL   517
ACM61646.1*GH106conserved   483  SYSYKVVVLPPL----------TNLR------------KTTLKLLQ------------NFVNTGGKIVAL   518
AGL50002.1*GH106putative_   482  EYEYEVVVLPPL----------LNLK------------SSTVELLN------------SLAENGGKVFVL   517
AGC67072.1*GH106hypotheti   537  DMKYEVLIVPEL----------ITIR------------SETVKMLI------------NHLENGGKVIIL   572
CAA72194.1_GH13_alpha-amy   425  NADVDGCIYDDP--------YDGFSVEEQES--DPKSLLNFIRFIL------------NFRKDHDAILNG   472
Consensus_aa:                    ........................................phh..h..............hhp.stphh..
Consensus_ss:                       eeeee                                hhhhhhh                hhhh     eeee


Conservation:
3cih_chainA_p005                 ----------------------------------------------------------------------
WP_008765775.1_GH106_BT_0   824  PYK---------------EDDFSS--------------YGLERDLIVPE--------------------   843
5mqm_chainA_p001            784  PYK---------------EDDFSS--------------YGLERDLIVPE--------------------   803
BAC68538.1_GH78_SaRha78A_   896  RWDS-IQPDGGFQT-PEMNSFNHY--------------AYGSVGEWMYAN-------------------   929
3w5n_chainA_p002            894  RWDS-IQPDGGFQT-PEMNSFNHY--------------AYGSVGEWMYAN-------------------   927
AAO76120.1_GH78_putative_   790  LWNG-DTANP------GMNSGNHV-------------MLLGDLLPWCFNN-------------------   819
CCA90848.1_GH106_RHA-P__N   823  APIA-TPSNTSA------QEGDLT--------------EWSSLVARLWPGSGDARVG----KGRVIASQD   867
BAD12237.1_GH106_alpha-L-   799  APER-----SPA------LQDDPT--------------AFRALVARLWNGAPVTPVG----QGRVIAETD   839
CDF79921.1_GH78_alpha-L-r   757  RWNS-YSKAEGFNP-MKMNSLNHY--------------AYGAIGEWMYER-------------------   790
WP_084817526.1_GH106_hypo   604  KPKK-------------SPSLEHYST---------CDAEVKRLSDFLWGKGLV-------------KEIS   638
BAB62315.1_GH78_rhamnosid   842  MYPN-FAENRSNPD-MLTRSHCH---------------AWSAAPGYFLG--------------------   873
2okx_chainA_p004            840  MYPN-FAENRSNPD-MLTRSHCH---------------AWSAAPGYFLG--------------------   871
WP_018627535.1_GH106_dist   595  NPLS-RIDGAAMTP-GERFSDDR---------------RGVAVFTELDDTVI-----------------   629
ACT02314.1_GH106_dist._gl   598  EELPSRTSEGL-------------------------------DAADELFTLANHPKV-------KRVALDI   630
AEX05711.1_GH78_KoRha__Kl   487  IFDP-AHPDFSPYGSKLINSYCH---------------AWSCTPAWFIRQ-------------------   520
4xhc_chainA_p003            477  IFDP-AHPDFSPYGSKLINSYCH---------------AWSCTPAWFIRQ-------------------   510
NP_813056.1_GH106_hypothe   589  ENYP-TDV----------PGYGQLEQ---------KRKTYQQTLQKLPSVSFSETTVTPVGKGKIITGTD   638
CAB53341.1_GH78_Ram78A_al   722  RWNS-MLPDGKVSD-TGMNSFNHY--------------SYGSIVEWIYRN-------------------   755
AAV43293.1_GH78_alfa-l-rh   713  RWNS-VLPDGKMNP-EGMNSLNHY--------------SFGAVMMWMYQC-------------------   746
```

```
ACM23671.1*GH106Glycoside   518  KD-------GNFFP-ERVEGRKEKPEFLK---RARVFEDLQGLIDSLRD---------------------   555
ACM61646.1*GH106conserved   519  KDFM----FSRFEL-CMIDGSKCDIPLKE---KFKNASDLNELVNILKE---------------------   559
AGL50002.1*GH106putative_   518  KDFR----YGRFFP-ERVEGKKGRIEFLK---KARVFETLEDLIEELKP---------------------   558
AGC67072.1*GH106hypotheti   573  GNLP-KII----------DGDSDRKFDIECLGECVINNSKYDLLDALEPFR-------------------   612
CAA72194.1_GH13_alpha-amy        ----------------------------------------------------------------------
Consensus_aa:                    ...s...............t..p...................h..bh.......................
Consensus_ss:                                                          hhhhhhhhh

Conservation:                                                    5            5 5
3cih_chainA_p005                 ----------------------------------------------------------------------
WP_008765775.1_GH106_BT_0   844  --------------------------NIAWTHRQ--GEQGD-IYFIANQLE-----ETRTFTASMRIDGR   879
5mqm_chainA_p001            804  --------------------------NIAWTHRQ--GEQGD-IYFIANQLE-----ETRTFTASMRIDGR   839
BAC68538.1_GH78_SaRha78A_   930  ---------------------------IAGIAPG--RAGYR-QVVIRPRPGG----EVTSARATFASLH-   964
3w5n_chainA_p002            928  ---------------------------IAGIAPG--RAGYR-QVVIRPRPGG----EVTSARATFASLH-   962
AAO76120.1_GH78_putative_   820  ---------------------------LAGIRADRWKSGYK-HIVFQPAFEI---QELSNVDASYMSIY-   857
CCA90848.1_GH106_RHA-P__N   868  IESALQAMDVAPDFTFTGADA---GVKIPFVHRR--DGKGE-IYYLVNQQE-----AAQSIEAHFRVTGK   926
BAD12237.1_GH106_alpha-L-   840  VEKALAGIGIGPDFSFAGAGP---DADLRFLHRK--LADGD-LYFIRNGLF-----RPEKIEARFRVTGR   898
CDF79921.1_GH78_alpha-L-r   791  ---------------------------ITGIAPL--QAGYK-IISIAPIPKA----PLTSASATLNTPY-   825
WP_084817526.1_GH106_hypo   639  IVDFLKGNNLLADFKIESDD----VSDISFIHRK--TDEAD-IYFIANARK-----ESREIKVRFRVSNK   696
BAB62315.1_GH78_rhamnosid   874  -------------------------SSILGVKRG--ADGWR-TVDIAPQPC-----DLTWAEGVVPLPQ-   909
2okx_chainA_p004            872  -------------------------SSILGVKRG--ADGWR-TVDIAPQPC-----DLTWAEGVVPLPQ-   907
WP_018627535.1_GH106_dist   630  -----DRIFRSPGFSIHMSTPPPEGGSLLHHRRI--IGDGQ-LLFLSNAN------LTQPAAGYVDVNGN   685
ACT02314.1_GH106_dist._gl   631  LAQELTASGYYEI-RADQY-----QPYLRNYHYQ--HEGLDVWMFFNEHPY-----QTLDTKLELPVSIQ   687
AEX05711.1_GH78_KoRha__Kl   521  ---------------------------YGL----------------------------------------   523
4xhc_chainA_p003            511  ---------------------------YGL----------------------------------------   513
NP_813056.1_GH106_hypothe   639  YARTLASCNIPQEEMKT-------KFGLQAIRRV--NDSGH-HYFISSLQD-----KGVNDWVTLGTKAK   693
CAB53341.1_GH78_Ram78A_al   756  ---------------------------AAGIQPVEDAPGFR-RFRLKPQPHY----LLKSLDAEFLSPA-   792
AAV43293.1_GH78_alfa-l-rh   747  ---------------------------VVGLNQF--DAGFK-EIYFAPKFDC----RLKDIYSEFDSTY-   781
ACM23671.1*GH106Glycoside   556  ----VAAVEV----IDKKTGK--NAKSVIAQKRL--LENGSYIIFLANTD------INREVHCQLNLKEE   607
ACM61646.1*GH106conserved   560  ----EVSNYIEV--IDKKTGQ--NAKKIIFQNRK--LNDGNRIIFLANTG------LQREAEITIKILTD   613
AGL50002.1*GH106putative_   559  ----FFSVDV----LDTKTKE--NAKAVIAQKRV--LEDGSYLLFLANTD------IDREVHCHLELKEK   610
AGC67072.1*GH106hypotheti   613  ----------QIDIVNRDSSR---NENYLYQLRD--EAECK-WLFIAPGKPVEALKLPEEKVLTVKVRGS   666
CAA72194.1_GH13_alpha-amy   473  ----------DQTIFRD-------WKNLIAFYRE--SSNEK-LLVVLNPDP-----VWQNSFTFEENMT-   516
Consensus_aa:                    ...........................l..h.........c.hhhl.s..........pph.h.h.....
Consensus_ss:                                               eeeeeee        eeeee            eeeeeee

Conservation:
3cih_chainA_p005                 ----------------------------------------------------------------------
WP_008765775.1_GH106_BT_0   880  K--PECWNPVTGEINAD---IPYEQKS---HRTEITLTLAPNESVFIVYPAEED-------DKETSEKE-   933
5mqm_chainA_p001            840  K--PECWNPVTGEINAD---IPYEQKS---HRTEITLTLAPNESVFIVYPATG-----------------   884
BAC68538.1_GH78_SaRha78A_   965  ------------GPVST----RWQQRS---GGFVLTCSVPPNTTAEVWIPADHP-------DRVQHTH--  1006
3w5n_chainA_p002            963  ------------GPVST----RWQQRS---GGFVLTCSVPPNTTAEVWIPADHP-------DRVQHTH--  1004
AAO76120.1_GH78_putative_   858  ------------GKITS----RWKKTP---MHLEWDIELPANTTGEVHLPDGRK-------EKIGSGK--   899
CCA90848.1_GH106_RHA-P__N   927  Q--PELWHPETGKSEPI----SYRISG---GETVVPLHLDGDEAVFVVFRKAAA-------RDRVTLARQ   980
BAD12237.1_GH106_alpha-L-   899  Q--PELWRATDGAVQPL----SYRTEG---SQTVVSLDVGAEDAFFILFRKPAD-------VPALTIAQP   952
CDF79921.1_GH78_alpha-L-r   826  ------------GEVAS----SWEIKN---ETLFLEVVVPPNTTAEIEIPTDNS-------ESLKVDN--   867
WP_084817526.1_GH106_hypo   697  Q--PEIWQAESGTIKKP--AVWQNHAD---GTTSLPLQLGMEEAVFVVFKNASK-------EKSQLVSA-   751
BAB62315.1_GH78_rhamnosid   910  -----------GGHIAV----SWEFVS--AGKLKLRIEAPEDIE--------------------------   936
2okx_chainA_p004            908  -----------GGHIAV----SWEFVS--AGKLKLRIEAPEDIE--------------------------   934
WP_018627535.1_GH106_dist   686  KF-ILMMDLSTGTILNY----PQTARQ---QGTRFSFDIPPAGSALFFIADHRQ-------KAGGTYRQ-   739
ACT02314.1_GH106_dist._gl   688  GD-VYAYDAYLNRLSKV---KAIGKGG---ELKTVQLVLSPYESVVLLHGKDLE-------TCTA-LPSY   742
AEX05711.1_GH78_KoRha__Kl        ----------------------------------------------------------------------
4xhc_chainA_p003                 ----------------------------------------------------------------------
NP_813056.1_GH106_hypothe   694  A--AALFNPMTGECGEA----KVRQAG---EQTQVYLQLKSGESVILQTYQQPL-------QAARPWKY-   746
CAB53341.1_GH78_Ram78A_al   793  ------------EKIIS----RWNINE--NGSVSFYFRIPFNTTAELVLPDTEV-------QDWK-----   832
AAV43293.1_GH78_alfa-l-rh   782  ------------GKIKV----EYHLETNEKHLIRMNLVIPFGVKMKVKLPRSAK-------YLIN-----   823
ACM23671.1*GH106Glycoside   608  KRHIYAIDIFDFAVVEF----SER-----------EFTMHPASSLLIWVTDEEIPSQRRSVISTGVTLK-   661
ACM61646.1*GH106conserved   614  KN-VFVADLVDFGVFKIPTLRRENGCA------VIDATMYPASSLCLLVSSKELSQN-TKNVISGVVFD-   674
AGL50002.1*GH106putative_   611  RKHTYAIDLFNFKLVEL----KEN-----------EFVMFPASSVCIWVTDEEVPAEDEKVVSTGVLLE-   664
AGC67072.1*GH106hypotheti   667  YE-VWRYDTFTGEKQKI----QARGKD---GWTYFEQTAYEHDSFLYRLLKTGTNDTTEKSVSSGVGRQV   728
CAA72194.1_GH13_alpha-amy   517  -----MILEVDFENFIW----NESNVS---FSAGESFTVDPMKAYIFKK---------------------   553
Consensus_aa:                    ......................bp..s.......h.hph...pshbh.h.....................
Consensus_ss:                                 eeee    eeee      eeeeeeee    eeeeee

Conservation:
3cih_chainA_p005                 ----------------------------------------------------------------------
WP_008765775.1_GH106_BT_0   934  -RKEKKDSVKEA-SETGLEAT-------------------------------------------------   952
5mqm_chainA_p001            885  -----------------LEAT-------------------------------------------------   888
BAC68538.1_GH78_SaRha78A_  1007  -GTFVR-----------AEDG-------------------------------------------------  1015
3w5n_chainA_p002           1005  -GTFVR-----------AEDG-------------------------------------------------  1013
AAO76120.1_GH78_putative_   900  -YHFSV-----------DIPTRNAAIISDEFLYEKASFPECHGATIVELKNGDLVASFFGGTKERNPDCC   957
CCA90848.1_GH106_RHA-P__N   981  GERAVA-----------TLDG-------------------------------------------------   990
BAD12237.1_GH106_alpha-L-   953  DMRPVA-----------TLSA-------------------------------------------------   962
CDF79921.1_GH78_alpha-L-r   868  -ENFTN----------------------------------------------------------------   872
WP_084817526.1_GH106_hypo   752  -KMELENPKSEPLSNLQIIKAEYGTFLQEGLVDITDKVAAEVKDNQLHIQASRAFCDCDPAMGYIKE-FR   819
BAB62315.1_GH78_rhamnosid        ----------------------------------------------------------------------
2okx_chainA_p004                 ----------------------------------------------------------------------
WP_018627535.1_GH106_dist   740  -LHTEEEL----------KGSAITVQRPAANTLMIDFCDLQIAGSEKKDLHVRDATQLVFKENGFSRDPW   798
ACT02314.1_GH106_dist._gl   743  AVEAVF-----------ELTG-------------------------------------------------   752
AEX05711.1_GH78_KoRha__Kl        ----------------------------------------------------------------------
4xhc_chainA_p003                 ----------------------------------------------------------------------
NP_813056.1_GH106_hypothe   747  -IQEQPFS--------LSLDH-------------------------------------------------   758
CAB53341.1_GH78_Ram78A_al        ----------------------------------------------------------------------
AAV43293.1_GH78_alfa-l-rh   824  -----------------GKEK-------------------------------------------------   827
ACM23671.1*GH106Glycoside   662  -REFELEM----------AFSEFETEMNGFNVLPVDRVDMFEA------------DGKVFR-DEYVSKIW   707
ACM61646.1*GH106conserved   675  -NSFEYKT----------GSCEFDIALKNYNTLILDRIKYEV-------------DGKVIFEDCYCAQVW   720
AGL50002.1*GH106putative_   665  -KEFDFET----------ALNDFEVKMNSFNVLPVDRVEYFEA------------GGRVFR-NEFVSKIW   710
```

```
AGC67072.1*GH106hypotheti  729   NKGVKYI---------KYLNGPWEYRLSEKNVLLLDMARYSLDNGEISEREEVLRIDDKIRTKLGYPLRT  789
CAA72194.1_GH13_alpha-amy        ----------------------------------------------------------------------
Consensus_aa:                    ......................................................................
Consensus_ss:
```

```
Conservation:
3cih_chainA_p005                 ----------------------------------------------------------------------
WP_008765775.1_GH106_BT_0        ----------------------------------------------------------------------
5mqm_chainA_p001                 ----------------------------------------------------------------------
BAC68538.1_GH78_SaRha78A_        ----------------------------------------------------------------------
3w5n_chainA_p002                 ----------------------------------------------------------------------
AAO76120.1_GH78_putative_  958   IWVCRKPKGAKEWTAPKLAADGVFSLKDSQAALAGIDSTCTPVVDAKGKLT--ARRKACWNPVLFQIPGG 1025
CCA90848.1_GH106_RHA-P__N        ----------------------------------------------------------------------
BAD12237.1_GH106_alpha-L-        ----------------------------------------------------------------------
CDF79921.1_GH78_alpha-L-r        ----------------------------------------------------------------------
WP_084817526.1_GH106_hypo  820   MEYQIGEDIKTISAQEKEYVNINAGDKKLTVLKAVFGKFKPETKGVPKHYP--VHDVTEKIKQEIASGNL  887
BAB62315.1_GH78_rhamnosid        ----------------------------------------------------------------------
2okx_chainA_p004                 ----------------------------------------------------------------------
WP_018627535.1_GH106_dist  799   DHQMQFRTDFVQRDTFSRGSGFTVSYHFSIGDRVDAKNFRAVIEQGHLWND--IRVNGHPLTPGNSWWLD  866
ACT02314.1_GH106_dist._gl        ----------------------------------------------------------------------
AEX05711.1_GH78_KoRha__Kl        ----------------------------------------------------------------------
4xhc_chainA_p003                 ----------------------------------------------------------------------
NP_813056.1_GH106_hypothe        ----------------------------------------------------------------------
CAB53341.1_GH78_Ram78A_al        ----------------------------------------------------------------------
AAV43293.1_GH78_alfa-l-rh        ----------------------------------------------------------------------
ACM23671.1*GH106Glycoside  708   YEFYR----------LPEGTPFGVVYSFEIREK--PEKLFLVVECAENLDR--ITVNGHEAVFKKKGCTF  763
ACM61646.1*GH106conserved  721   HKHFYN---------LPEGTPFKATYHFELEKV--PSKLFVAIECAENLDM--ILVNNQPVKFERKSESF  777
AGL50002.1*GH106putative_  711   YEFYR----------LPDGTPFRVEYSFEVRKK--PQKLFLVVECAENLDR--ITVNGREVRYERKSCIF  766
AGC67072.1*GH106hypotheti  790   DSFPQPWLS---PAEA---------------ECPHTVKLYFEIESETDVHEAELAFEGKGVTAEWNNVAV  841
CAA72194.1_GH13_alpha-amy        ----------------------------------------------------------------------
Consensus_aa:                    ......................................................................
Consensus_ss:
```

```
Conservation:
3cih_chainA_p005                 ----------------------------------------------------------------------
WP_008765775.1_GH106_BT_0        ----------------------------------------------------------------------
5mqm_chainA_p001                 ----------------------------------------------------------------------
BAC68538.1_GH78_SaRha78A_        ----------------------------------------------------------------------
3w5n_chainA_p002                 ----------------------------------------------------------------------
AAO76120.1_GH78_putative_ 1026   DLILFYKIGLNVGDWTGWLVRSKDGGKTWGKREALPEGFLGPIKNKPEYINGR-------IICPSSREGK 1088
CCA90848.1_GH106_RHA-P__N        ----------------------------------------------------------------------
BAD12237.1_GH106_alpha-L-        ----------------------------------------------------------------------
CDF79921.1_GH78_alpha-L-r        ----------------------------------------------------------------------
WP_084817526.1_GH106_hypo  888   VIPVNNQLIGGKTPEGDNTTIKITFTTDGEEQTLFVPKGRPLNLSKDRSKPEIVLNDGETQWITPYPGTL  957
BAB62315.1_GH78_rhamnosid        ----------------------------------------------------------------------
2okx_chainA_p004                 ----------------------------------------------------------------------
WP_018627535.1_GH106_dist  867   RSFT------------------------------VLNAGPYLKEGNNTLS------------LTIDPMSI  894
ACT02314.1_GH106_dist._gl        ----------------------------------------------------------------------
AEX05711.1_GH78_KoRha__Kl        ----------------------------------------------------------------------
4xhc_chainA_p003                 ----------------------------------------------------------------------
NP_813056.1_GH106_hypothe        ----------------------------------------------------------------------
CAB53341.1_GH78_Ram78A_al        ----------------------------------------------------------------------
AAV43293.1_GH78_alfa-l-rh        ----------------------------------------------------------------------
ACM23671.1*GH106Glycoside  764   TEEECFLDVNFG----------------------KMDITELVQVGKNIVV------------LEGKKSNN  799
ACM61646.1*GH106conserved  778   SPDQNFLDVNIG----------------------KIDITSFVKEGKNEIV------------LSGRKSNN  813
AGL50002.1*GH106putative_  767   NEEQNFLDVNFG----------------------KMEITDLVREGKNTVV------------LEGRKENN  802
AGC67072.1*GH106hypotheti  842   TDRADGFFIDKCF--------------------NVMKLPG-IKKGVNQLV------------LTVPFGEK  878
CAA72194.1_GH13_alpha-amy        ----------------------------------------------------------------------
Consensus_aa:                    ......................................................................
Consensus_ss:
```

```
Conservation:
3cih_chainA_p005                 ----------------------------------------------------------------------
WP_008765775.1_GH106_BT_0  953   -------------------------EYTVTFTANGKTI------------------------QRQELFDW  973
5mqm_chainA_p001           889   -------------------------EYTVTFTANGKTI------------------------QRQELFDW  909
BAC68538.1_GH78_SaRha78A_        ----------------------------------------------------------------------
3w5n_chainA_p002                 ----------------------------------------------------------------------
AAO76120.1_GH78_putative_ 1089   ------------------------GGWRIHFEYSDDKGKTW---------------------KTTESVPA 1113
CCA90848.1_GH106_RHA-P__N  991   -------------------------AWQVAFQADRGAPASI---------------------ELARLEPL 1014
BAD12237.1_GH106_alpha-L-  963   -------------------------PWKVTFQQGRGAPASL---------------------TMKTLAPL  986
CDF79921.1_GH78_alpha-L-r        ----------------------------------------------------------------------
WP_084817526.1_GH106_hypo  958   SYKNLSGKVMATTVKSVPQPIMLAGTWDVEFPSDLVTINKV---------------------RFDELKSW 1006
BAB62315.1_GH78_rhamnosid        ----------------------------------------------------------------------
2okx_chainA_p004                 ----------------------------------------------------------------------
WP_018627535.1_GH106_dist  895   YAE--------------IGPVFLLGDYNLESAAKGWKI------------------------VPPKPLTT  926
ACT02314.1_GH106_dist._gl  753   -------------------------EWSVSIAEAVHYPSFKQ------------------WGQLAALSNM  779
AEX05711.1_GH78_KoRha__Kl        ----------------------------------------------------------------------
4xhc_chainA_p003                 ----------------------------------------------------------------------
NP_813056.1_GH106_hypothe  759   -------------------------GWKLHFAESEPKIKGT--------------------FDIDRPCSW  783
CAB53341.1_GH78_Ram78A_al  833   --------------------------EFMSLNV-------------------------------------  839
AAV43293.1_GH78_alfa-l-rh  828   -------------------------IGIVKLEY-------------------------------------  835
ACM23671.1*GH106Glycoside  800   --------------------ITGPGCHERVKNPENHRPTEVETIYLVGKFSLVNVDETKYVIDAPKTPDH  849
ACM61646.1*GH106conserved  814   --------------------ITAPGCHERVKDPKNHRPTEVEAIYLVGSFSLICVDETRFILTEPKKPCH  863
AGL50002.1*GH106putative_  803   --------------------ITGPGCHTRVKDPENHRPTEVETIYLVGDFSLVNVDETRYVIDAPKIPDH  852
AGC67072.1*GH106hypotheti  879   TD---------------LEWCFITGNFGTCVKGDKVIIT-----------------------NPPERLFF  910
CAA72194.1_GH13_alpha-amy        ----------------------------------------------------------------------
Consensus_aa:                    ......................................................................
```

```
Consensus_ss:


Conservation:
3cih_chainA_p005                ----------------------------------------------------------------------
WP_008765775.1_GH106_BT_0  974  SKEEDEQ------IRYYSGT--AVYK-----------TTFRWKSKVKE-------------DQQVYLNLG 1011
5mqm_chainA_p001           910  SKEEDEQ------IRYYSGT--AVYK-----------TTFRWKSKVKE-------------DQQVYLNLG  947
BAC68538.1_GH78_SaRha78A_       ----------------------------------------------------------------------
3w5n_chainA_p002                ----------------------------------------------------------------------
AAO76120.1_GH78_putative_ 1114  ELSVPTQNRKKGGINVDDQEAGEAIQGEGAQPILAIQPSILKHK-----------------DGRLQVLCR 1166
CCA90848.1_GH106_RHA-P__N 1015  DKSADPG------VKYFSGI--ATYS-----------RNFRVTGKYGE-------------GRSLWLDLG 1052
BAD12237.1_GH106_alpha-L-  987  NESADPG------VRYFSGI--ATYT-----------TSFALPRGVKP-------------GAPLSIDLG 1024
CDF79921.1_GH78_alpha-L-r       ----------------------------------------------------------------------
WP_084817526.1_GH106_hypo 1007  SAVENEG------IKYFSGT--ASYH-----------KTFQVSKKLLK------------SNNKLELDLG 1045
BAB62315.1_GH78_rhamnosid       ----------------------------------------------------------------------
2okx_chainA_p004                ----------------------------------------------------------------------
WP_018627535.1_GH106_dist  927  GSWRKQG------LPMYGHA--ILYK-----------KDYTLDAP----------------GNSYVLRLD  961
ACT02314.1_GH106_dist._gl  780  S--RPDY------LPRFSGT--FRYE-----------TEFEWSES----------------SEQAILELG  812
AEX05711.1_GH78_KoRha__Kl       ----------------------------------------------------------------------
4xhc_chainA_p003                ----------------------------------------------------------------------
NP_813056.1_GH106_hypothe  784  TGIDHPA------AKINMGS--GVYS-----------LDIELPALQ---------------ADDWVLDLG  819
CAB53341.1_GH78_Ram78A_al  840  ----------------------VNIL-----------ILTCLKDLTR-----------------------  853
AAV43293.1_GH78_alfa-l-rh  836  ----------------------GKYD-----------ISYIPTKSYLNYYDLNSKLVDILDNNDLVKRID  872
ACM23671.1*GH106Glycoside  850  KDITRSG------YPFYVGS--FTLR-----------KTFEMKKDP---------------KRRYFLKLN  885
ACM61646.1*GH106conserved  864  FDITKDG------YPFYVGS--VSLK-----------SSFEITKES---------------SKRVYIKLN  899
AGL50002.1*GH106putative_  853  RDITRDG------YPFYVGS--FTLK-----------KIFECKKDP---------------GKRYFLKLN  888
AGC67072.1*GH106hypotheti  911  GDVTRQG------LSFYGGN--ITYI-----------VNIDVPE------------------GEMTVEIP  943
CAA72194.1_GH13_alpha-amy       ----------------------------------------------------------------------
Consensus_aa:                   ......................................................................
Consensus_ss:                                         eeee           eeeee                     eeeee


Conservation:
3cih_chainA_p005                ----------------------------------------------------------------------
WP_008765775.1_GH106_BT_0 1012  KV-----------CDLATVRVNGIDCGTIWTA-----------PYRADITA----------------ALK 1043
5mqm_chainA_p001           948  KV-----------CDLATVRVNGIDCGTIWTA-----------PYRADITA----------------ALK  979
BAC68538.1_GH78_SaRha78A_       ----------------------------------------------------------------------
3w5n_chainA_p002                ----------------------------------------------------------------------
AAO76120.1_GH78_putative_ 1167  TR----------NAKIATSWSSDN--GETWSKVTLSNVPNNNSGTDAVTMSDGRHILIYNHFSTLPGTPK 1224
CCA90848.1_GH106_RHA-P__N 1053  RV-----------GDLAQVSVNGVDVGTAWHA-----------PYRLDIGK----------------AVR 1084
BAD12237.1_GH106_alpha-L- 1025  AI-----------GDLAEVRVNGQLAGASWFA-----------PHRLDIGK----------------WVK 1056
CDF79921.1_GH78_alpha-L-r  873  ----------------------------------------------GKNLK----------------LIK  880
WP_084817526.1_GH106_hypo 1046  SV-----------AVIAEVILNGKPVGTLWKA-----------PFRLDVTN----------------DVK 1077
BAB62315.1_GH78_rhamnosid  937  ----------------VNVTLPEGIEGEVTQV-----------KYMS-----------------------  956
2okx_chainA_p004           935  ----------------VNVTLPEGIEGEVTQV-----------KYMS-----------------------  954
WP_018627535.1_GH106_dist  962  SL----------KGSLAAVRVNGKNAGIVYAD-----------PYLLDVSK----------------LLK  994
ACT02314.1_GH106_dist._gl  813  EV-----------YETAEVWLNGEQAGVRICP-----------PYRMEISG----------------LLK  844
AEX05711.1_GH78_KoRha__Kl       ----------------------------------------------------------------------
4xhc_chainA_p003                ----------------------------------------------------------------------
NP_813056.1_GH106_hypothe  820  DV-----------RESARVRINGQEAGCAWAV-----------PYQLRVGH----------------LLK  851
CAB53341.1_GH78_Ram78A_al  854  ----------------------------------------------KCTE-----------------QIL  860
AAV43293.1_GH78_alfa-l-rh  873  QIDEKILQKVKRMGNTRSIFINKK------------------------IDE----------------LLD  902
ACM23671.1*GH106Glycoside  886  SV----------RAASVEVFLNEKFVGSLFWE-----------PFMVEITD----------------NLH  918
ACM61646.1*GH106conserved  900  SV----------SAAVAKVFVNGKKTCTLFSQ-----------PFLADITE----------------YVN  932
AGL50002.1*GH106putative_  889  GV----------EAASVEVILNGKFLGVLFWR-----------PFMIDITD----------------ALR  921
AGC67072.1*GH106hypotheti  944  QY----------RGALVEVALDNAKKGDIIFS-----------PYRLNLG-----------------YVS  975
CAA72194.1_GH13_alpha-amy       ----------------------------------------------------------------------
Consensus_aa:                   ......................................................................
Consensus_ss:                                  eeeeee                         ee                   h


Conservation:
3cih_chainA_p005           697  ------------------VWRLWIDSPEE-----------------------------------------  707
WP_008765775.1_GH106_BT_0 1044  KGV--------------NELEIEVTNTWANALKGADEGKAPFDG-----------------------IWT 1076
5mqm_chainA_p001           980  KGV--------------NELEIEVTNTWANALKGADEGKAPFDG-----------------------IWT 1012
BAC68538.1_GH78_SaRha78A_ 1016  ------------------CAVFEVGSG------------------------------------------- 1024
3w5n_chainA_p002          1014  ------------------CAVFEVGSG------------------------------------------- 1022
AAO76120.1_GH78_putative_ 1225  GPR--------------TPLCIAISEDGINWKPIL----------------------------------T 1246
CCA90848.1_GH106_RHA-P__N 1085  KGQ--------------NTLEIRVANTWVNRLIGDQQEGAQKI------------------------TWT 1116
BAD12237.1_GH106_alpha-L- 1057  PGS--------------NRLEVKVANLWVNRLIGDQQSGAEKT------------------------TFT 1088
CDF79921.1_GH78_alpha-L-r  881  NEK--------------RKIKILAQPG-------------------------------------------  893
WP_084817526.1_GH106_hypo 1078  TGE--------------NKLEVKVTNLWPNRLIGDEKLPLDFERKGPKIKSVPDWLLNNTKRPSERTTFP 1133
BAB62315.1_GH78_rhamnosid       ----------------------------------------------------------------------
2okx_chainA_p004                ----------------------------------------------------------------------
WP_018627535.1_GH106_dist  995  KGK--------------NRIEVTVVGSLRNLMGPHYKSYRP--------------------------GYT 1024
ACT02314.1_GH106_dist._gl  845  KGI--------------NKLVIEVTNTLAKDQRDFL----------------------------------  866
AEX05711.1_GH78_KoRha__Kl       ----------------------------------------------------------------------
4xhc_chainA_p003                ----------------------------------------------------------------------
NP_813056.1_GH106_hypothe  852  SGK--------------NHIEIEVTNLPANRISELDRQGVQWRKFKEI----------------NIVDLN  891
CAB53341.1_GH78_Ram78A_al  861  QCG--------------KFMKMRGQKKL------------------------------------------  874
AAV43293.1_GH78_alfa-l-rh  903  FEEISQEEKNQLVDILHKTIFIKLNLF-------------------------------------------  929
ACM23671.1*GH106Glycoside  919  DGK--------------NELKLVLTNTLFNLIEANHKADVLEETFRR------------------PWSFI  956
ACM61646.1*GH106conserved  933  DGK--------------NELEIVLTNTLFNLIEANHKADVFDELYRR------------------PQSFI  970
AGL50002.1*GH106putative_  922  NGK--------------NELQLVLTNTLFNLIEANHKADVLEETFRR------------------PKSFI  959
AGC67072.1*GH106hypotheti  976  QGE--------------HTIEITLYGNRFNMFGQLHNCNRVEKYYGP-------------------NTWR 1012
CAA72194.1_GH13_alpha-amy       ----------------------------------------------------------------------
Consensus_aa:                   ...................l.l.lss............................................
Consensus_ss:                                     eeeeee
```

```
Conservation:
3cih_chainA_p005           708  ----------------------------------RIVTYRL------------  714
WP_008765775.1_GH106_BT_0 1077  NAKYR-RAENTLLPAG-----LLG----------PLNFDVANKNK-------- 1105
5mqm_chainA_p001          1013  NAKYR-RAENTLLPAG-----LLG----------PLNFDVAN----------- 1038
BAC68538.1_GH78_SaRha78A_ 1025  ---------------------SHR----------FTV---------------- 1030
3w5n_chainA_p002          1023  ---------------------SHR----------FTVK--------------- 1029
AAO76120.1_GH78_putative_ 1247  LEDSP---ISQYSYPSIIQGKDGKLHAIYTWRRQRIKYTEIDLSKFK------ 1290
CCA90848.1_GH106_RHA-P__N 1117  AMPTY-RADAPLRPSG-----LIG----------PVRLIEETTGGH------- 1146
BAD12237.1_GH106_alpha-L- 1089  AAPTY-RPDAPLRPSG-----LIG----------PVTLIAE-TGDR------- 1117
CDF79921.1_GH78_alpha-L-r  894  ---------------------TYE----------FQAKYSL------------  903
WP_084817526.1_GH106_hypo 1134  AWKHW-DKEDELLSSG-----LLG----------PVKINVLVEKSL------- 1163
BAB62315.1_GH78_rhamnosid       -----------------------------------------------------
2okx_chainA_p004                -----------------------------------------------------
WP_018627535.1_GH106_dist 1025  GPDSW---SGITGYPA-----GHA----------YILFDYGLNDIRLFRNEKD 1059
ACT02314.1_GH106_dist._gl  867  ------SSFAQQEPSG-----LIG----------PVTVKKITEPAGRPIRT--  896
AEX05711.1_GH78_KoRha__Kl       -----------------------------------------------------
4xhc_chainA_p003                -----------------------------------------------------
NP_813056.1_GH106_hypothe  892  YRPAN-YGHWAPMPSG-----LNS----------EVRLIPVDYLSFKTH----  924
CAB53341.1_GH78_Ram78A_al       -----------------------------------------------------
AAV43293.1_GH78_alfa-l-rh       -----------------------------------------------------
ACM23671.1*GH106Glycoside  957  DFEHH-TDRYILLPFG-----LEN----------AAVLSSFSP----------  983
ACM61646.1*GH106conserved  971  DFENF-TSRYMILPFG-----LGS----------YSILTSNV-----------  996
AGL50002.1*GH106putative_  960  DFEHH-TDRYILLPFG-----LEN----------VAVLSSSSR----------  986
AGC67072.1*GH106hypotheti 1013  TKGNAFAYEYQLREFG-----IIA----------APLIYIE------------ 1038
CAA72194.1_GH13_alpha-amy       -----------------------------------------------------
Consensus_aa:                   .....................................................
Consensus_ss:                                                     eeeee
```

Figure S1. PromalS3D alignment for generation of the neigbour-joining tree (Fig.1).
Protein sequences (proteins in supplementary **Table S3**), and PDB structures from one GH106 enzyme and four GH78 rhamnosidases, as well as one outgroup have been aligned:
3cih_chainA_p005 (GH78 AAO76108.1, BT_1001, *Bacteroides thetaiotaomicron* VPI-5482), **WP_008765775.1** and **5mqm_chainA_p001** (GH106 BT_0986, *Bacteroides thetaiotaomicron* VPI-5482, **BAC68538.1** and **3w5n_chainA_p002** (GH78 SaRha78A, *Streptomyces avermitilis* MA-4680 = NBRC 14893), **AAO76120.1** (GH78 BT1013, *Bacteroides thetaiotaomicron* VPI-5482), CCA90848.1 (GH106 RHA-P PP1Y_Mpl10172, *Novosphingobium* sp. PP1Y), **BAD12237.1** (GH106 RhaM, *Sphingomonas paucimobilis* FP2001 / JCM 10661), **CDF79921.1** (GH78 BN863_22090, *Formosa agariphila* KMM 3901), **WP_084817526.1** (GH106 CDF79916.1, BN863_22040, *Formosa agariphila* KMM 3901), **BAB62315.1** and **2okx_chainA_p004** (GH78 RhaB, *Bacillus* sp. GL1), **WP_018627535.1** (GH106 (_dist) B160DRAFT_04058, *Niabella aurantiaca*), **ACT02314.1** (GH106 (_dist) Pjdr2_3683, *Paenibacillus* sp. JDR-2), **AEX05711.1** and **4xhc_chainA_p003** (GH78 KoRha, *Klebsiella michiganensis* KCTC 1686), **NP_813056.1** (GH106 BT_4145, *Bacteroides thetaiotaomicron* VPI-5482), **CAB53341.1** (GH78 RamA, *Thermoclostridium stercorarium* NCIB 11754), **AAV43293.1** (Gh78 RamA, *Lactobacillus acidophilus* NCFM), following proteins from this study are indicated with *GH106: **ACM23671.1** (GH106 CTN_1495, *Thermotoga neapolitana* DSM 4359, homolog to Tn_Ram106B of *Tt. neapolitana* Z2706-MC24), **ACM61646.1** (GH106 Cb_Ram106B, Athe_2581, *Caldicellulosiruptor bescii* DSM 6725), **AGL50002.1** (GH106 Tm_Ram106B, Tmari_1078, *Thermotoga maritima* MSB8), **AGC67072.1** (GH106 Ts_Ram106B, Cst_c00400, *Thermoclostridium stercorarium* subsp. *stercorarium* DSM 8532), **CAA72194.1** (GH13_36 AmyA, *Thermotoga maritima* MSB8)

```
Conservation:                                             57     7                     55          5 96
AAO76093.1 BT_0986 B. the   400  MGHTA-----TGHTNATA-GGGKGLECDKFNPKAVRKQFDNWFAQAFVKTNP-DVARRVLKYMHVDS--W   460
5mqm_chainA_p001 BT_0986    360  MGHTA-----TGHTNATA-GGGKGLECDKFNPKAVRKQFDNWFAQAFVKTNP-DVARRVLKYMHVDS--W   420
CCA90848.1 RHA-P Novosphi  443  LGYSL-----LGTTNHPAPPEATGLEVDKFDGEAVREYLEHYIGMYKDAAGPDMVGKRGVRALLTDS--I   505
BAD12237.1 RhaM Sphingomo  419  MGYSL-----TGKTNHPATPEATGLEVDKYDPAAVRRYLETYLGLYRDAVGAEWIGKKGIRALLTDS--I   481
CDF79916.1 BN863_22040 Fo  243  LGHTP-----TGKKNHPAPKGGHGLEVDKMSTKAVDVYWEGGIQPILNKLGD-LVGTT-VNNCLIDS--Y   303
WP_018627535.1 B160DRAFT_  206  KLSYTNQAGMVGPPNY--------PLVDLLVKGVTEKFIDITFSGYKKAFGD-AFGKT-VPGTFSDEPSI   265
ACT02314.1 Pjdr2_3683 Pae  195  ISESK-----DGGSAAQ------EDYLNPLVRDSVRILLDTVYEAVYKRYKA-DFGST-FAGFFSDE--P   249
AAO079250.1 BT_4145 B.the  196  LYNSK-----TRQKVKRAAPGGEGYVMNHLSKTAVKNYLSRFNRAFKSSKTS-Y-----PHTFFNDS--Y   252
AGC67072.1 Ts_Ram106B The  184  VISGD-----LPWFNNQ-------AYVDTLNKKAVEKFIEVTHEKYFEILGG-EFSKS-VPAVFTDEPQF   239
ACM23671.1 CTN1495 Tn_Ram  143  LGDPW-----FNGT----------CYVDLLSKKVTEAFIRSTHEKYRKVCGE-FFGSS-IPGIFTDEPTY   195
ACM61646.1 Cb_Ram106B Cal  144  LGDKW-----FNGS----------CYVDLLSKEATLEFINLTHERYKSYCQD-YFGDA-MPGIFTDEPTY   196
AGL50002.1 Tm_Ram106B The  143  LGDPW-----FNGT----------CYVDLLSRETTEAFLRSTHERYKKSCGD-LFRVS-IPGIFTDEPTY   195
Consensus_aa:                    h.ps......hs............bhs.hs.phhc.hhc.hh..hbp.hss..htpp.h.shhsDp..h
Consensus_ss:                    ee                      eeee   hhhhhhhhhhhhhhhhhhhhh hhhhh

Conservation:                                         87  5   8     965     76 6           555 55
AAO76093.1 BT_0986 B. the   461  ECG-----------------SQNWSDTFAAEFRKRRGYDLMPYLPLLAGIPMESAERSEKILRDVRTTIG   513
5mqm_chainA_p001 BT_0986    421  ECG-----------------SQNWSDTFAAEFRKRRGYDLMPYLPLLAGIPMESAERSEKILRDVRTTIG   473
CCA90848.1 RHA-P Novosphi  506  EVG-----------------EANWTPRMLEQFQRLRGYDARPWLPALTGTLVGTREQSDRFLYDYRRTLA   558
BAD12237.1 RhaM Sphingomo  482  EVG-----------------ASNWTPRMVEEFRARRGYDPVPFLPALTGAVVGSAARSDAFLHDFRQTLA   534
CDF79916.1 BN863_22040 Fo  304  EVG-----------------TANWTAGFDAEFETLRGYSLVSYLPTLAGYYVESGEITERFLWDFRRTIG   356
WP_018627535.1 B160DRAFT_  266  PTHG--------------SLTTRWTPDLFAVFKEKWGYDLVPHLPSLFEETGD----WRKIRHNYQETLL   317
ACT02314.1 Pjdr2_3683 Pae  250  GFYNDKTTFDFQSRPGKKGVPLPWSSAMPGLLEQALGGDYRKKLYLLWQEDE--SAQFNAVRYAYMNIVS   317
AAO079250.1 BT_4145 B.the  253  EVY-----------------QADWTEDFLDQFARRRGYKLEEHFPEFLDENR--PEISRRIVSDYRETIS   303
AGC67072.1 Ts_Ram106B The  240  VLKQNLP-----EPDSRQEVGIPYTDLFEESFRQRFGCSFLDSLPEIFWELEG--GKFSRIRYWYHEHIA   302
ACM23671.1 CTN1495 Tn_Ram  196  LRVH-----------YPEIPTLPWTDRFPEEFLKRKGYDIREHFEELFFNTK----DYMKVRYDFFDVAT   250
ACM61646.1 Cb_Ram106B Cal  197  LRVH-----------YKDIATLPWTEKLPERFLQKKGYDIKEHFEELFFNVG----NYHKVRFDFFDIAL   251
AGL50002.1 Tm_Ram106B The  196  LRVH-----------HPKETTLPWTERFPEEFLRRKGYDIRDHLEELFFNVK----DYMKVRYDFFDVAT   250
Consensus_aa:                    ....................shsWo..h...F.p..Ghsh...h..lh..........p+h..s@.phhs
Consensus_ss:                    e                              hhhhhhhh     hhhhhhhhh         hhhhhhhhhhhhh

Conservation:                     8            5 5      55                          5      6 85 5 5
AAO76093.1 BT_0986 B. the   514  ELVVDVFYQVLADCAKEYDCQFSAECVAPT-------------------MVSDGLLHYQKVDLPMGEFWL   564
5mqm_chainA_p001 BT_0986    474  ELVVDVFYQVLADCAKEYDCQFSAECVAPT-------------------MVSDGLLHYQKVDLPMGEFWL   524
CCA90848.1 RHA-P Novosphi  559  DLLASEHYGTVADVAHENDLKVYGEALEDHR-----------------PMLGDDMAMRSHADIPMAALWT   611
BAD12237.1 RhaM Sphingomo  535  DLLADAHYGTIAKVAHEQGLIVYGEALENGR-----------------PVLGDDLAMRAHADVPMAALWT   587
CDF79916.1 BN863_22040 Fo  357  DLMAKNYYAHFRDLCHKNGLKFSVEPYWG---------------------PFDNMQVGATGDIVMCEFWS   405
WP_018627535.1 B160DRAFT_  318  QLFIDRWSKPMQAFTQKNHLKWTGHYWEHGWPD--------------PGEGPDNMAMYAWHDQPGIDMLF   373
ACT02314.1 Pjdr2_3683 Pae  318  KLYADNFCSQIGDWCRARGVEYIGHVLEDNNVHARLGPGPGHFFRSLWGQGMSGLDVVLWQIVPGFDELS   387
AAO079250.1 BT_4145 B.the  304  DLLLENFTRQWTDWAHKNGSITRNQAHGS---------------------PANLIDVYAAVDIPECEGFG   352
AGC67072.1 Ts_Ram106B The  303  ELFASSFADTVGRWCREHNLMLTGHMMAESTLESQ------------TRALGEAMRSYRSFDLPGVDILA   360
ACM23671.1 CTN1495 Tn_Ram  251  ELFIENFTIPYAKWCEENGISMTGHYMAEDTLRGQ------------VEWIGAAMPHYEYMQIPGVDKLA   308
ACM61646.1 Cb_Ram106B Cal  252  EMFIENFTIPYAKWCEENGIMMTGHYMAEDTMRGQ------------IEWIGAAMPHYEYMQLPGVDKLA   309
AGL50002.1 Tm_Ram106B The  251  SLFIENFTIPYAKWCEENGIFMTGHYMAEDTLRGQ------------VEWIGAAMPHYEYMQIPGIDKLA   308
Consensus_aa:                    cLhhcs@h..h.chtccpshbh.schh..s......................ssh.h....plP.h-bh.
Consensus_ss:                    hhhhhhhhhhhhhhhhhh  eeeee                           hhhhhhh

Conservation:                                               8   9 9 5 66     5 9 56      6   5   9   9   7
AAO76093.1 BT_0986 B. the   565  NSP--------------THDKPNDMLDAISGAHIYGKN--IIQAEGFTEVRGTWNEHPGILKALLDRNYA   618
5mqm_chainA_p001 BT_0986    525  NSP--------------THDKPNDMLDAISGAHIYGKN--IIQAEGFTEVRGTWNEHPGILKALLDRNYA   578
CCA90848.1 RHA-P Novosphi  612  FNRD--------EGP--RQTLIADMKGAASVAHLYGQN--LVAAESMTASMAPWAFAPKDLKRFIDLEFV   669
BAD12237.1 RhaM Sphingomo  588  YNRG--------SAP--RPTLIGDMKGAASVAHIYGQN--IVSAESMTAAFSPWAFAPADLKRVIDLEFV   645
CDF79916.1 BN863_22040 Fo  406  GG----------------YPFFDSPKFVSSIAHLNGSS--IVGAESFTGIG-GWDEHPAELKSIGDRAWA   456
WP_018627535.1 B160DRAFT_  374  NQFN-------ESSPNAQFGNIRSVKELSSVANQLGKE--RTLCETYGGA--GWDLTFKDMKRLGDWQAA   432
ACT02314.1 Pjdr2_3683 Pae  388  FRNT-------SGEADSEFFHYGLAKLGVSLAHIDPKKQGRTMCEVYGAY--GWTEGLKLMKWLTDHMLV   448
AAO079250.1 BT_4145 B.the  353  LSQFHIKGLRQDSLTKKNDSDLSMLKYASSAAHIAGKP--YTSTETFTWLTEHFRTSLSQCKPDMDLMFV   420
AGC67072.1 Ts_Ram106B The  361  NK-----------------YEYSTVKQAQSVARQFGKA--GVLSELYGVT--NWDFDFRGHKLQGDWQAA   409
ACM23671.1 CTN1495 Tn_Ram  309  RH----------------LEQVITIKQVSSVAEQLRKK--WVLCETFGTT--GQHVSFLHRKWIADWQAV   358
ACM61646.1 Cb_Ram106B Cal  310  RH----------------LEQVVTMKQVSSVAEQLGKK--GVLCETFGTT--GQHVSFLHRKWISDWQAV   359
AGL50002.1 Tm_Ram106B The  309  RH----------------LEQVVTIKQVSSAAEQLGKK--WVLCETFGTT--GQHVSFLHRKWIADWQAV   358
Consensus_aa:                    .p...................hs.hK.h.ShAcb.Gp....h.tEs@s.h..sbp......K.b.D..hh
Consensus_ss:                                          hhh   hhhh     ehhhhhhhhh         hhhhhhhhhhhhh
```

Figure S2. Structural alignment for rhamnosidases of CAZy family GH106 (as of May 2019). The alignment was made using default settings of PromalS3D, with sequences of characterized GH106: AAO76093.1 (BT_0986 from *Bacteroides thetaiotaomicron* VPI-5482) and its structure derived from PDB (5mqm_chainA_p001), CCA90848.1 (RHA-P from *Novosphingobium* sp. PP1Y), BAD12237.1 (RhaM from *Sphingomonas paucimobilis* FP2001/JCM 10661), CDF79916.1 (BN863_22040 from *Formosa agariphila* KMM 3901), two distant GH106 enzymes, now also assigned to GH106: WP_018627535.1 (B160DRAFT_04058 from *Niabella aurantiaca* and ACT02314.1 (Pjdr2_3683 from *Paenibacillus* sp. JDR-2), AAO079250.1 (BT_4145 from *Bacteroides thetaiotaomicron* VPI-5482), and the four rhamnosidases of this study: AGC67072.1 (Ts_Ram106B, Cst_c00400 from *Thermoclostridium stercorarium* subsp. *stercorarium* DSM 8532), ACM23671.1 (GH106 CTN_1495, *Thermotoga neapolitana* DSM 4359, homolog to Tn_Ram106B of *T. neapolitana* Z2706-MC24), ACM61646.1 (Cb_Ram106B, Athe_2581 from *Caldicellulosiruptor bescii* DSM 6725) and AGL50002.1 (Tm_Ram106B, Tmari_1078 from *Thermotoga maritima* MSB8). Only blocks containing at least one amino acid contributing to the active center of BT0986 (AAO76093.1, WP_008765775.1; PDB: 5MQM) are shown. Background colors show possible general acid (green E461), residues possibly binding calcium (yellow D458, S459, E538, E561) and possible general base (magenta E593) which were derived from the crystal structure of BT_0986 and suggested by Ndeh et al. (2017). Indicated positions refer to the full-length sequence (AAO76093.1).

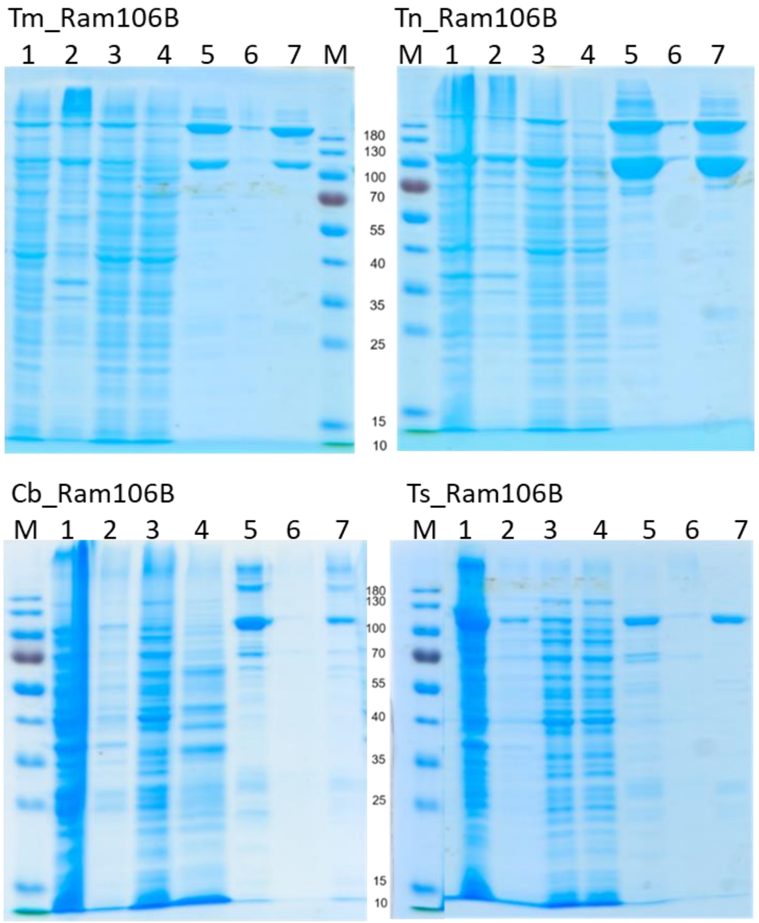

Figure S3. SDS-PAGE of protein purification. 1. Cell extract, 2. Resuspended pellet after sonification, 3. Supernatant after sonification, 4. Flowthrough IMAC, 5. Pooled elution fractions IMAC, 6. Pellet after heat treatment, 7. Supernatant after heat treatment

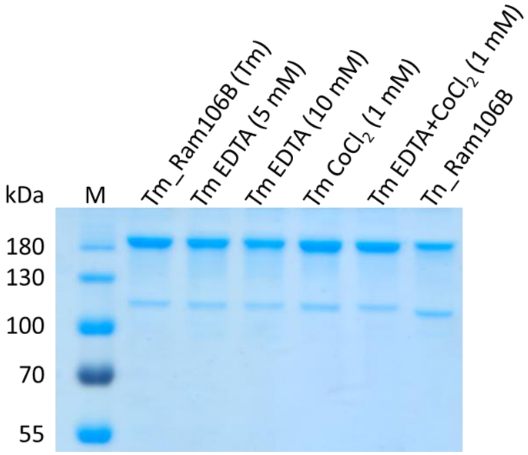

Figure S4. Influence of EDTA on oligomerisation state. SDS-PAGE of samples containing 1.7 µM enzyme, Tm_Ram106B (Tm, monomer: 116.6 kDa) or Tn_Ram106B (monomer: 115.8 kDa) with EDTA and or CoCl2 where indicated with final concentrations as shown in brackets

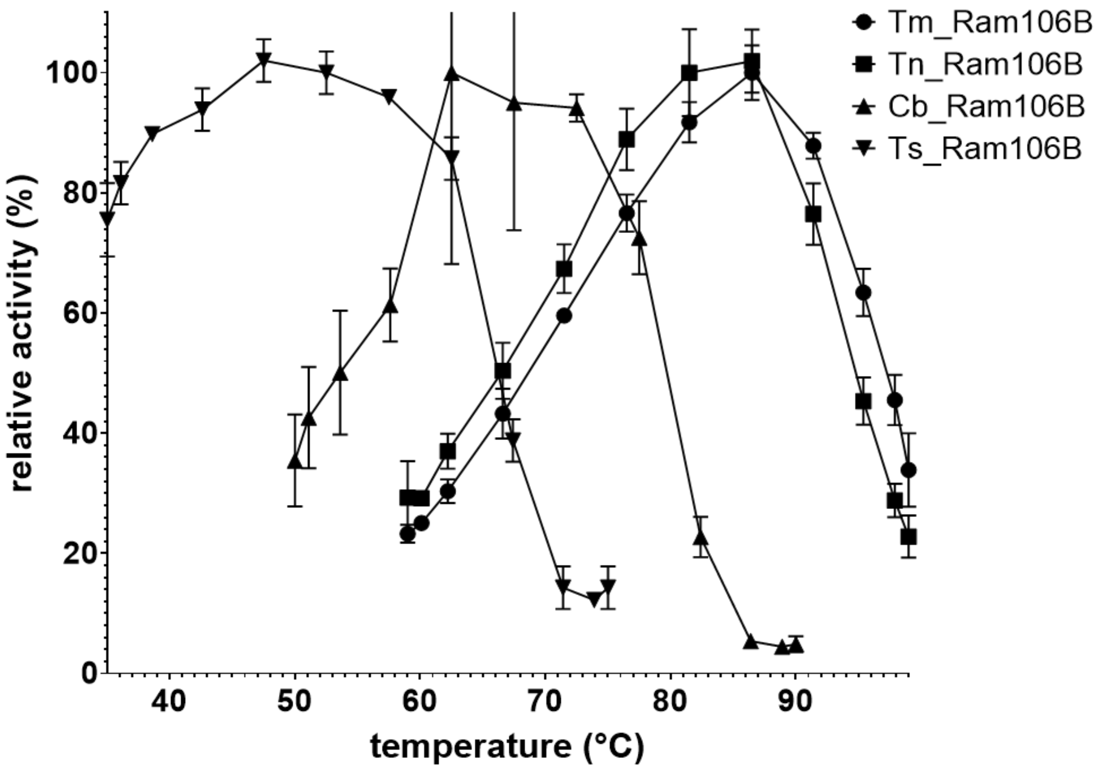

Figure S5. Effect of temperature on rhamnosidases from *T. maritima*, *T. neapolitana*, *C. bescii* and *Tc. stercorarium*. Relative activities to the temperature optima were calculated from pNP standard reactions (1 mM pNPR, 100 mM MOPS pH 7, 10 min) with 1 mM $CoCl_2$ and 10 nM Tm_Ram106B, 10 nM Tn_Ram106B, 50 nM Cb_Ram106B, or 50 nM Ts_Ram106B. Error bars represent standard deviation of triplicates.

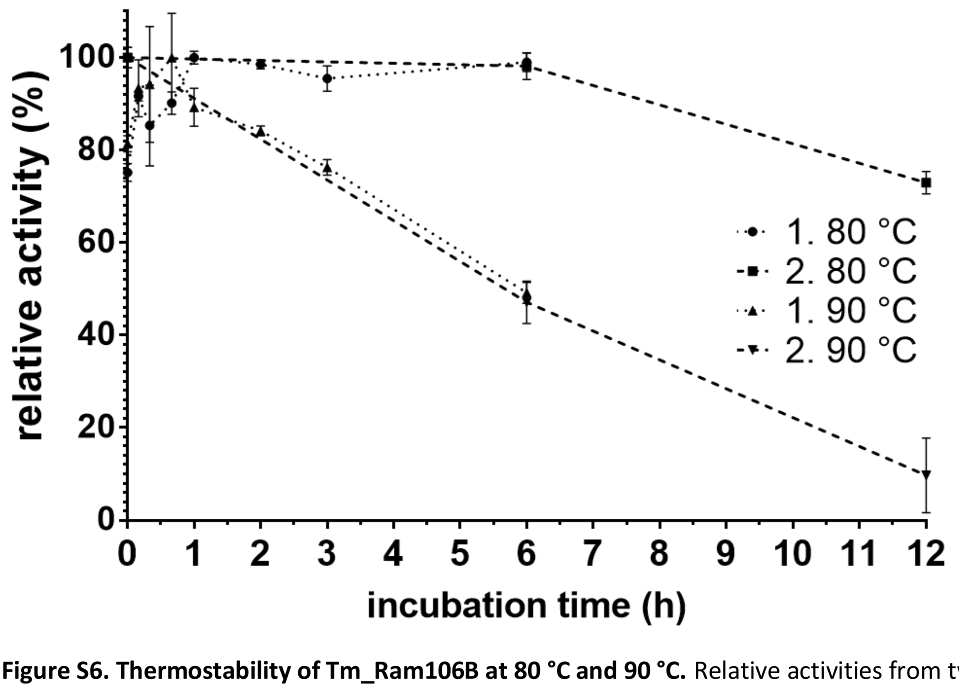

Figure S6. Thermostability of Tm_Ram106B at 80 °C and 90 °C. Relative activities from two independent measurements (1. Dotted line: 0–6 h; 2. Dashed line: 0, 6, 12 h), Highest measured activity at 80 °C was set to 100 % for each time course. Enzyme was preincubated for indicated times at 80 °C or 90 °C. Standard pNPR reactions (1 mM pNPR, 1 mM $CoCl_2$ 100 mM MOPS pH 7 RT, 80 °C, 10 min) were performed with 50 nM enzyme. Error bars represent standard deviations of triplicates.

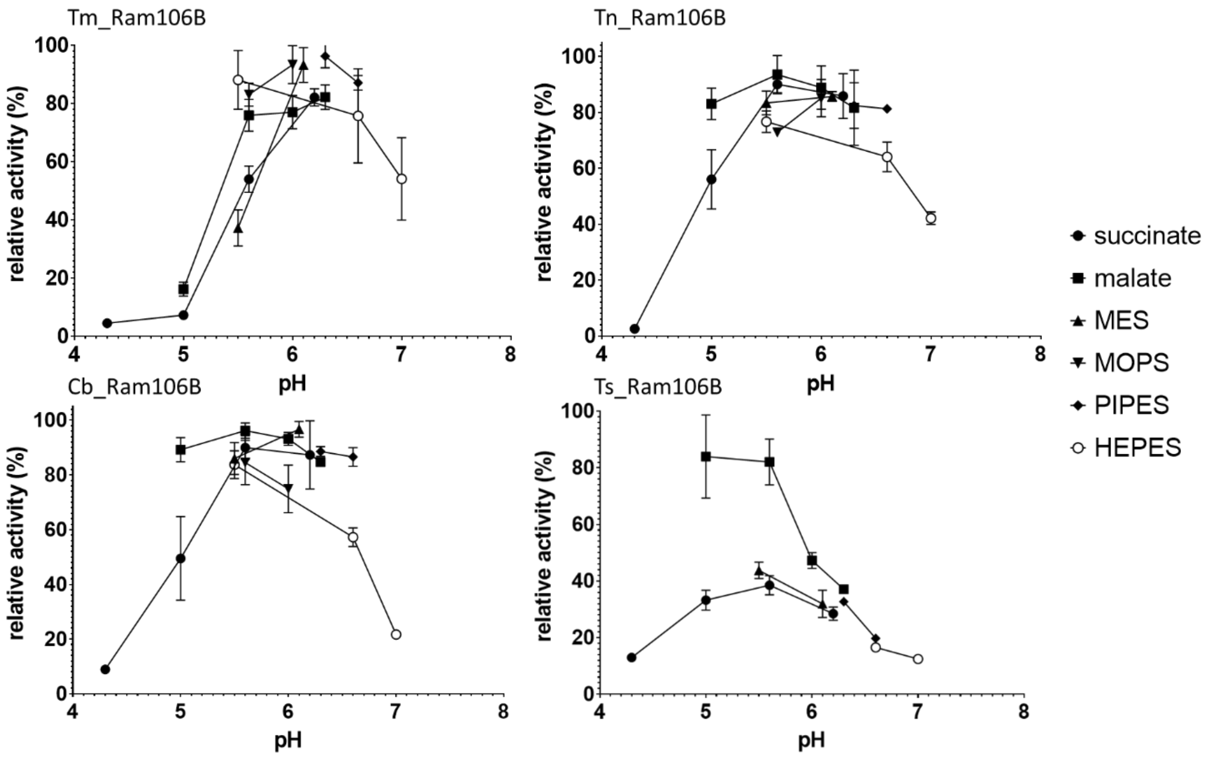

Figure S7. Effect of pH on rhamnosidases from *T. maritima*, *T. neapolitana*, *C. bescii* and *Tc. stercorarium*. Relative activities, to the highest measured activity of each enzyme, were calculated from pNP standard reactions as described in materials and methods (1 mM pNPR, 1 mM CoCl2, 10 min, 80 °C for Tm_Ram106B and Tn_Ram106B, 70 °C for Cb_Ram106B, or 55 °C for Ts_Ram106B) with enzyme concentrations of 50 nM. Standard buffer has been substituted by: 25 mM succinate, 50 mM malate, 50 mM MES, 100 mM MOPS, 25 mM PIPES or 100 mM HEPES with different pH. Error bars represent standard deviation of triplicates.

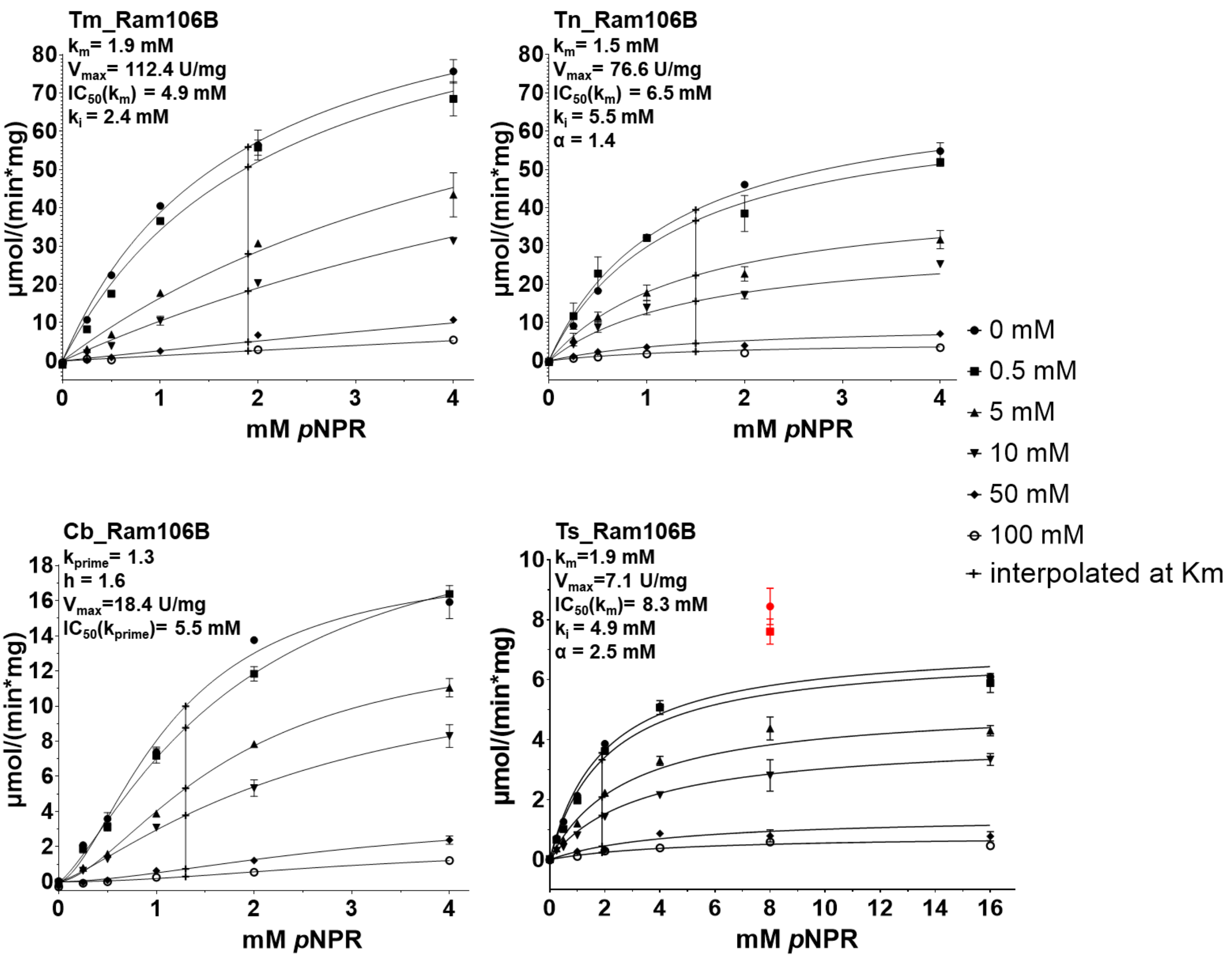

Figure S8. Determination of kinetic and inhibition parameters of rhamnosidases Tm_Ram106B, Tn_Ram106B, Cb_Ram106B and TsRam106B degrading *p*NPR. Standard reactions (1 mM pNPR, 1 mM $CoCl_2$, 100 mM MOPS pH 7, 10 min) were performed with enzyme concentrations, reaction temperatures and amendments, if necessary, as indicated in brackets: Tm_Ram106B (2.5 nM, 80 °C), Tn_Ram106B (10 nM, 80 °C), Cb_Ram106B (5 nM, 70 °C), Ts_Ram106B (50 nM; 55 °C; 50 mM malate pH 4.6 RT). Error bars represent standard deviation of duplicates. Data points not included in the analysis are marked in red. K_m, the equivalent K_{prime}, and V_{max} were computed using Graphpad Prism 7, as described in material and methods. IC_{50} values were determined by using interpolated data points at the respective Km (connected by vertical line)
